# A new cancer progression model: From synthetic tumors to real data and back

**DOI:** 10.1371/journal.pcbi.1013991

**Published:** 2026-06-24

**Authors:** Daniela Volpatto, Sandro Gepiro Contaldo, Simone Pernice, Marco Beccuti, Francesca Cordero, Roberta Sirovich

**Affiliations:** 1 Department of Computer Science, University of Torino, Turin, Italy; 2 Department of Mathematics G. Peano, University of Torino, Turin, Italy; 3 Laboratorio InfoLife, Consorzio Interuniversitario Nazionale per l’Informatica (CINI), Rome, Italy; University of Connecticut School of Medicine, UNITED STATES OF AMERICA

## Abstract

Intratumor heterogeneity (ITH) arises from the combined effects of genetic alterations, clonal interactions, and environmental constraints, and plays a central role in therapeutic resistance and disease progression. While ITH has been extensively documented in empirical tumor data, the scientific debate regarding the biological mechanisms underlying this heterogeneity remains complex, highlighting the need for cancer evolution models that are sufficiently flexible and sophisticated to reproduce the observed behaviors and to give insights on the unobserved ones. Here, we present a stochastic modelling framework for tumor evolution that integrates genotypic inheritance with phenotype driven functional traits and resource mediated competition. Mutational events are associated with functional capabilities such as altered proliferation, increased mutation rates, limit evasion potential or enhanced control over shared resources, allowing multiple genotypes to converge on similar phenotypes. The model explicitly tracks subclonal lineages while incorporating environmental constraints that modulate growth and competition. The framework is defined through a mathematically rigorous construction and is accompanied by an efficient simulation algorithm. To facilitate exploration and reproducibility, we provide an open-source graphical user interface that allows users to configure model parameters, run simulations, and inspect clonal genealogies and population dynamics without requiring direct interaction with the underlying code. Using this model, we illustrate how ecological feedbacks can shape clonal dynamics over time, supporting an interpretation in which early tumor growth is dominated by stochastic expansion, while later evolution increasingly reflects selection for traits that alleviate environmental constraints. Rather than constituting a new evolutionary paradigm, this behaviour demonstrates how well-documented biological patterns can emerge naturally from a unified stochastic and ecological description. Overall, our approach offers a flexible and extensible platform for investigating how chance, functional traits, and environmental interactions jointly govern tumor heterogeneity.

## Author summary

Not all cancerous cells are created equal: inside the same tumor, different populations of cells exist at the same time, fighting for the same resources and influencing the way the disease evolves and reacts to treatments. These groups of cells have different behaviour and abilities thanks to different genetic mutations, which might give them an advantage or bring their population to disappearance. We have built a mathematical model that mimics the evolution of a tumor over time, simulating a competition between its different populations of cells. Our simulated experiments show that tumors evolve in two distinct phases: at first, cells that grow and divide more quickly have an advantage. Once the space and nutrients are limited, cells that can survive with fewer resources have an advantage and can potentially take over the race. We use these simulations to argue that the evolution of a tumor doesn’t depend on the shape of the space it expands in, but rather on the availability of nutrients.

## Introduction

Cancer is a profoundly heterogeneous disease, characterized by the coexistence of multiple subpopulations of cells within a single tumor, each potentially carrying distinct genetic, epigenetic, and phenotypic features. This phenomenon, known as *intratumor heterogeneity*, poses significant challenges for diagnosis, prognosis, and treatment [1–4]. Understanding the dynamics and structure of the cancer heterogeneity is crucial to improving patient outcomes. In particular, intratumor heterogeneity (ITH) drives therapeutic resistance, makes it difficult to detect cancer driving mechanisms and often underlies relapse after initially successful treatment [5–9].

Traditional approaches to cancer research, largely grounded in molecular biology and genomics, have illuminated many aspects of tumor heterogeneity enabling detailed characterization of tumor cell populations, their genetic mutations, metabolic phenotypes, and microenvironmental features. However, these experimental methods often struggle in capturing the dynamics of tumor evolution, especially the temporal dimension that traces how subclonal populations emerge, expand, and compete over time [8,10,11]. Reconstructing the evolutionary history of a tumor remains one of the key challenges in this field indeed. In human patients, serial biopsies across disease progression are rarely feasible due to ethical and practical constraints; as a result, most evolutionary inferences must be drawn from single timepoint data, leveraging the fact that the genetic diversity within a tumor encodes a molecular archive of past mutational events.

This challenge has given rise to the interdisciplinary field of *tumor evolution*, which draws on concepts from evolutionary biology, ecology, and population genetics to study how tumor cells adapt to selective pressures over time [1,5,12]. Within this framework, mathematical modeling has emerged as a powerful complementary approach [13,14]. Mathematical models allow researchers to go beyond observational data and test hypotheses about the underlying processes that shape heterogeneity. They enable simulations of evolutionary scenarios and predictions of clonal behaviour under different selective pressures by formalizing assumptions and linking them to measurable outcomes.

According to [11], theories regarding evolutionary dynamics of tumors proposed in the past decade can be conceptualized through four main models: *Linear Evolution*, according to which new driver mutations sequentially outcompete previous clones, resulting in a dominant clonal population [15,16]; *Branching Evolution* where multiple subclones arise from a common ancestor and evolve in parallel, creating a highly branched phylogenetic tree (this model is supported by numerous genomic studies and explains the persistent coexistence of multiple subclones within tumors) [17–22]; *Neutral evolution* posits that many mutations accumulate without strong selective pressure, resulting in a mixture of subclones with similar fitness and a characteristic allele frequency spectra [23,24]; in *Punctuated evolution*, tumors undergo a bursts of rapid genomic change, often associated with catastrophic events such as chromosomal rearrangements, at a very early stage of the disease. After this punctuated event, one or a few dominant clones stably expand to form the tumor mass [25]. Each model has different implications for diagnosis, prognosis, and treatment. Importantly, evidence suggests that they are not mutually exclusive; different regions of the same tumor, or different classes of mutations, may follow distinct evolutionary trajectories and the very same tumor might undergo modes in different stages.

Mathematical and computational models have been central to formalizing these concepts [25–27]. Mathematical models of tumor evolution broadly fall into two classes: well-mixed formulations and spatially structured formulations, each of which can be implemented through deterministic or stochastic approaches [13,14,28]. Deterministic models (typically ODE, PDE, or hybrid systems) offer biological detail and can capture growth–consumption dynamics, microenvironmental interactions, and spatial diffusion [29–33]. Their main limitation is that they describe average trajectories, which makes them suboptimal for representing intratumor heterogeneity, a phenomenon fundamentally driven by rare, stochastic events [28]. Agent-based models can also capture spatial heterogeneity at single-cell or component resolution, while accounting for stochasticity. However, they are often computationally intensive and allow limited analysis of mathematical properties, being mainly supported by simulations [28]. This leads back to the classical but powerful tools of stochastic processes for population evolution. Two main instruments have been exploited within this area: branching processes, which generate exponential growth by construction, and Moran (or modified Moran) processes, which impose a fixed population size. Attempts to introduce density dependence exist [34], but they remain restricted to a few population types or biologically narrow scenarios. Furthermore, in previously developed models, selective advantage is typically encoded only as a proliferative boost, despite the fact that fitness in tumors depends heavily on resource availability, tolerance to deprivation, and competitive or cooperative interactions among clones [13,16,34–37]. Evolutionary game-theoretic frameworks also have explored interaction-driven selection, though they tend to focus on equilibrium coexistence rather than full evolutionary trajectories [13,14]. Phenotype-driven models have been proposed, but they typically target specific mechanisms and capture only a narrow range of phenotypic classes, while ignoring genomic structure [13,38].

We propose here a new model for tumor evolution that integrates resource constraints, cellular interactions, and phenotypic rules, without losing the ability to represent genotypes and subclonal genealogies, by adopting a broad definition of clones and functional mutational events. Recent spatial stochastic models [39–42] move in a similar direction, but often rely on the assumption that spatial segregation is the primary driver of evolutionary mode. We argue that equivalent patterns can arise from interaction-mediated heterogeneity, even in the absence of sharp spatial segregation. Our goal is to provide a flexible, event-based stochastic framework that unifies evolutionary and ecological processes within a single architecture. It supports diverse functional events beyond driver and passenger schemas, incorporates carrying capacity to reproduce both early and late growth regimes, explicitly tracks subclonal genealogies, and is modular, making it straightforward to integrate emerging biological insights. In doing so, we bring together, to the best of our knowledge, aspects that have previously been studied only in isolation within a single coherent framework.

## Materials and methods

### The cancer progression model

Assuming that a tumor originates from a single cell that, as a result of one or more mutational events (genetic or epigenetic), gains the ability to proliferate uncontrollably, bypassing cellular growth regulation mechanisms and invading surrounding tissue, we model the evolution of its subclones as a stochastic process built over a rooted ordered tree. Over time, mutational events can accumulate, creating a heterogeneous set of cellular populations, each characterized by specific genetic traits that influence their ability to survive, replicate, resist therapies and interact with the surrounding environment.

Informally, the model we are going to describe can be thought of as a cell duplicating and dying at certain birth and death rates (*a* and *b*); sometimes, at a certain rate μ, the perfect duplication could fail, giving rise to a new subclone carrying an additional mutational event. In this framework, mutational events can encompass any genetic structural variation or epigenetic alteration. The only requirement is that these events are irreversible: once a cell acquires a mutation, it will retain it permanently and pass it on to all its descendant cells. A population (or clone) consists of cells that share a unique, defining genotype, which is stored with a unique identifier.

We aim to describe the size of each population of cells as it changes over time, hence we will build a process $X_{u}(t)$, where *u* will indicate the identifier of the population. Since only the original population starts its course at time 0, for all others, we will have a random time of appearance $\sigma_{u}$. Furthermore, we will need a structure to record the genetic relationships between populations, which will be constructed by exploiting *rooted ordered trees*, a mathematical structure that encodes the phylogenetic tree of a whole population.

To each population will be associated a *phenotype*. By phenotype, we refer to the set of functional traits that define a population with a specific genotype. A given phenotype may arise from different genotypes, as different mutations can lead to similar functional outcomes. The phenotype of a population will determine the rules governing its birth-and-death dynamics, the likelihood of new mutations arising, and the cells’ abilities to acquire additional resources and bypass the physiological constraints of a healthy organism.

**Fig 1:**
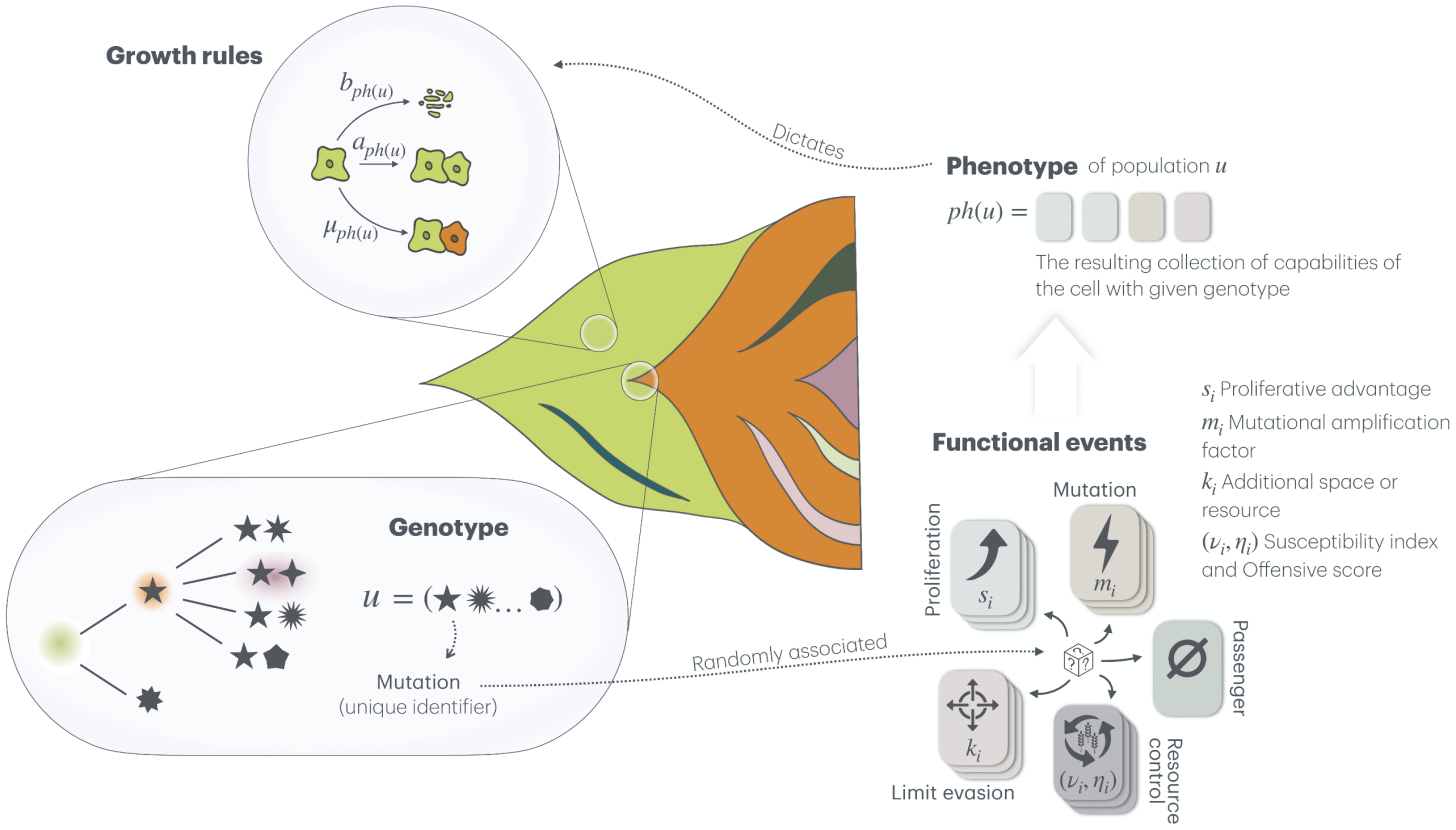
Visual representation of the cancer progression model.

The overall tumor model will result from the combination of these elements working at different levels. On one hand, we model the genetic makeup of cell populations that descend from a single ancestor and are organized within a tree of inheritance and parental relationships, which drive the evolutionary trajectory of the tumor. On the other hand, the model incorporates the mapping of each genotype to its observable biological effects-the phenotype-which contributes to tumor progression by influencing the final cellular composition. Note that the model is for a well-mixed population of cells, hence it is not including a spatial component that accounts for segregation or separation of different cellular populations. A graphical representation of the model is shown in Fig 1.

In the next Sections we will formally introduce each of these elements.

**Genotype and Rooted Ordered trees** The phylogenetic evolution of a tumor is described as a rooted tree τ. The generic element $u=(u_{1},\ldots ,u_{n})$ of the tree contains a collection of mutations and can be interpreted as a genotype, i.e., a *mutational profile*. We define |*u*|: = *n* the generation of *u*, $p(u)=(u_{1},\ldots ,u_{n-1})$ the parent of *u* and we will denote as $u{|}_{j}:=(u_{1},\ldots ,u_{j})$ the restriction up to generation *j* of the element *u*. A new genotype *v* branches from *u* at the (*n* + 1)-th generation when an additional mutational event arises, with parent equal to *u*, i.e., $p(v)=v{|}_{n}=u$. We will denote as $X_{u}(t)$ the number of cells, with genotype *u*, at time *t*.

**The phenotype as the genotype-associated functional capabilities of a cell.** The mutational events may or may not induce changes in the functional traits of the cell. We will refer to the collection of eventual alterations of the biological processes of a cell with a given genotype *u* as the *phenotype*, meaning with that the observable effect that the mutational events have on it. We are following the framework developed by D. Hanahan and R. A. Weinberg, first outlined in “The Hallmarks of Cancer” [43] and later expanded in [44] and [45], that organizes the diverse capabilities and enabling characteristics acquired during the multistep development of human tumors. They claim:

“We foresee cancer research developing into a logical science, where the complexities of the disease, described in the laboratory and clinic, will become understandable in terms of a small number of underlying principles. Some of these principles are even now in the midst of being codified. [...] We suggest that most if not all cancers have acquired the same set of functional capabilities during their development, albeit through various mechanistic strategies.”

These hallmark capabilities - each supported by specific mutations - describe how cancer cells diverge from normal cellular functions to support unchecked growth and adaptability within the body. We developed the phenotype part of our model using the conceptual framework provided by Hanahan et al. [43–45] with the aim of including recognised functional characteristics of human tumours and mapping them into a clear mathematical description. We propose that, from a mathematical modeling perspective, the functional capabilities can be further merged into five primary mechanisms that affect the evolutionary processes of the cells:

1. **Deregulation of the proliferation program** (dividing faster/dying slower). We map into this class all the functional capabilities that have an effect on the replication process of the cells: we may therefore include here the acquired abilities in sustaining the proliferative signaling, in evading growth suppressors, in deregulating the cellular metabolism, in resisting cells death, in enabling the replicative immortality and in avoiding the immune destruction. The simplified functional effect is a boost in the growth of the cell by either diminishing the expected time required before a cell encounters duplication -a progression through the cell cycle- and by increasing the replicative potential -immortality-, or enlarging lifespan -circumvention of the apoptotic program-. The homeostasis of cell number and the maintenance of normal tissue architecture and function is lost and a surplus in the number of births compared to the number of deaths is observed.
2. **Mutation burden augmentation** (mutating more often). Whenever the DNA is duplicated, there is a possibility of running into an error: this can be measured in terms of number of errors per cell division divided the number of base-pairs in order to obtain a standard mutation rate. There is evidence that the acquisition of the hallmarks of cancer is made possible by several enabling characteristics, among which the most prominent is the development of genomic instability that increases the mutation rate on tumor cells, as the succession of the alterations in the genomes of neoplastic cells results in the acquisition of function-altering mutations which enable the development of different capabilities.
3. **Limit evasion** (potential for expansion over defined physiological limits). Tumors are located within a body, hence they are subject to the physical constraints and to the limitation of the available resources. The infrastructure of the tissues in which cancer develops are built to bear a given number of cells. Tumor cells acquire the ability to invade nearby tissue and to disseminate, hence to escape the physiological size limits.
4. **Resource control**. The invasion process is supported by angiogenesis, which is reactivated and maintained to allow the formation of new blood vessels that help to sustain and expand neoplastic growth, and by the ability of adjusting the energy metabolism in order to fuel cell growth and division. As the capacity of the system is limited and the number of cells capable of living in such conditions is bounded, there is a natural “competition” for survival between different cells. The state of equilibrium where each cell has the same possibility to get access to the resources it need for living is lost and the ability to gain an advantage is acquired: the cell might need less nutrients for living by reprogramming the energy metabolism, it can actively harm the neighbours by subtracting nutrients, or it can become capable of exploiting resources that have been recruited by others. Yet, a combination between these “powers” might be advantageous, for instance if two cells both help the other and find resource in them, a mutualistic relationship could be created. All these events are grouped in this functional effect: those that tune how the resources are split among the cells.

We will consider another (non-)functional mechanism for our purposes of modelling:

1. **Null effect.** It is associated to mutations that do not lead to any observable or functional change in the cell, effectively behaving as neutral mutations.

Different mutations can induce the same functional effect, but possibly with different intensities: for instance, a mutation might lead to the mutation rate being doubled, while another could decuple it and both will be mutations that lie in class 2. Hence a functional capability is defined by two objects: the class of the primary mechanism and its intensity. For this reason we define the *functional event list F* as the set


$$\begin{array}{c} F=\{f_{1}=(F_{1},\theta_{1}),\ldots ,f_{I}=(F_{I},\theta_{I})\}, \end{array}$$ (1)

where *I* is the number of functional capabilities included, and $F_{i}$ corresponds to the class of primary mechanism (one among the 5 we described above) induced by the mutational event, and $\theta_{i}$ indicates the set of parameters, i.e., the intensity. Assuming that each mutation causes an alteration included in *F*, we define a function that associates each genotype $u=(u_{1},\ldots ,u_{n})$ to its *phenotype*:


$$\begin{array}{c} \text{𝓅𝒽}:\;\tau ⟶𝒫(F)\\ \;u⟼f=\{f_{i_{1}},\ldots ,f_{i_{k}}\},\;i_{j}\in \{1,\cdots ,I\} \end{array}$$ (2)

with


$$\begin{array}{c} \text{𝓅𝒽}(\emptyset ):=\;\text{phenotype of the initiating population}\\ \text{𝓅𝒽}(u):=\;\left\{ \begin{array}{c} \left\{\text{𝓅𝒽}(p(u)),f_{i_{j}}\}\text{ if }f_{i_{j}}\notin \text{𝓅𝒽}(p(h)\right)\\ \text{𝓅𝒽}(p(u))\text{ if }f_{i_{j}}\in \text{𝓅𝒽}(p(h)) \end{array}\right. \end{array}$$ (3)

The differentiation among the two cases (1) the newly acquired mutation induces a functional capability already gained through a former mutational event experienced by the parental population -$f_{i_{j}}\in \text{𝓅𝒽}(p(h))$- and (2) the newly acquired mutation induces a new functional capability -$f_{i_{j}}\notin \text{𝓅𝒽}(p(h))$-, is necessary to represent the functional redundancy of the gene network. Some mutations may target different regions of the DNA that encode subunits of the same protein complex. If the complex is already non-functional due to one mutation, a second mutation in another subunit of the same complex does not worsen the defect. Even mutations occurring in different genes that perform similar or overlapping roles within a pathway have a similar behavior: if either gene is mutated, the pathway might be disrupted or overactivated, leading to the same functional effect, while when both genes are mutated, there’s no additional impact because the pathway was already maximally altered by the first mutation. To model additive epistasis, it is enough to duplicate the functional event adding an element to *F*: as said before, the list of functional events *F* is superabundant with respect to the primary characteristics we have described and the reason of that is not only to consider effects of different intensities, but also to better characterize those mechanism that sum up.

The recursive definition of the *phenotype* function given in Eq. (3) sets by default the effect of different mutations as cumulative. In our model, we have chosen to support only additive interactions and neutral epistasis due to the need to balance model complexity with usability as incorporating non-additive forms of epistasis (such as positive, negative, or reciprocal sign epistasis) introduces a significant increase in the number of parameters. This choice is not a limitation of the model’s conceptual framework but rather a deliberate simplification aimed at enhancing its practical applicability. However, our framework remains flexible and extendable: incorporating non-additive interactions is entirely feasible should the need arise or when sufficient knowledge is available to justify the additional complexity.

**The population evolution model** For each genotype u∈τ we associate $\sigma_{u}\geq 0$, the birth time of *u*, and $\left(X_{u}(t),t\geq \sigma_{u}\right)$ the number of cells, with genotype *u*, at time *t*, the clone. We make the following choices. The process that determines the size of the cell population $\left(X_{u}(t),t\geq \sigma_{u}\right)$ is modeled as a *size-dependent birth and death process*. The *t*heory of size-dependent branching processes, which extends classical branching models by allowing reproduction and survival rates to depend on population size, has its origins in the mid-20th century and counts among its ranks distinguished mathematicians such as Klebaner and Jagers, see [46–48]. Despite the name, size-dependent branching processes do not satisfy the basic branching property, that corresponds to “Each individual evolves into a branching process independent from and identically distributed to all the others.” Indeed, the law governing a size-dependent branching process will depend on the size of the population, which makes it impossible for the evolution of an individual within many, to evolve following the same law as the individual generating the process alone. Recalling the idea that the functional mechanisms of a clone are completely defined by its *phenotype*, the birth and death rates of each population, $a_{\text{𝓅𝒽}(u)}$ and $b_{\text{𝓅𝒽}(u)}$, will be phenotype-associated (as well as dependent on the population size), i.e., the rates will depend both on the size of the clone and on the functional effect of the cumulated mutations. We can therefore define the process describing the number of cells sharing mutational profile *u* as:


$$\begin{array}{c} X_{u}(t-\sigma_{u})\sim SDBD(a_{\text{𝓅𝒽}(u)},b_{\text{𝓅𝒽}(u)}),\;t\geq \sigma_{u}\;X_{u}(t)=0,\;t<\sigma_{u}\;X_{u}(\sigma_{u})=1, \end{array}$$ (4)

where *SDBD* stands for Size-Dependent Birth and Death process. The extension before appearance of population *u* is straightforward as any population will be of zero individuals until its appearance, and then it will start with a single cell, the daughter of a cell with mutational profile *p*(*u*) having acquired an additional mutation. Population *u* arises from cells belonging to *p*(*u*), as the result of a duplication with a new mutational event cumulated in one of the daughter cells. The point process that describes the appearance of the genotype *u* is modeled as a *Doubly Stochastic* point process or *Cox* point process, see [49], with random measure


$$\begin{array}{c} \xi (0,t]=\int_{0}^{t}\mu_{\text{𝓅𝒽}(p(u))}X_{p(u)}(s)ds, \end{array}$$ (5)

where $\mu_{\text{𝓅𝒽}(p(u))}$ is the mutation rate associated to the phenotype of the parent of *u*. Hence, conditional on ξ, the point process is a Poisson process with parameter measure ξ. It follows that


$$\begin{array}{c} \mathbb{P}(\sigma_{u}\leq t|\xi )=1-\exp\left(-\mu_{\text{𝓅𝒽}(p(u))}\int_{0}^{t}X_{p(u)}(s,\omega )ds\right). \end{array}$$ (6)

We will set $\sigma_{\emptyset }=0$ with probability 1.

The set of genotypes appeared within time t, $N(t)=\{u:\sigma_{u}\leq t\}$, is almost surely a locally finite rooted tree for any *t*, see S1 Appendix for the proof. Notice tha*t* N(s)⊆N(t) for s≤t.

The stochastic process describing the tumor clonal evolution is


$$\begin{array}{c} X(t)=(X_{u}(t){)}_{u\in N(t)}, \end{array}$$ (7)

The process *X*(*t*) is a Markov process, see S1 Appendix for the proof, with generator


$$\begin{array}{c} 𝒜f(\tau ,𝐱)=\sum_{u\in \tau }[a_{\text{𝓅𝒽}(u)}(\tau ,𝐱)f(\tau ,𝐱+{𝐞}_{u})+b_{\text{𝓅𝒽}(u)}(\tau ,𝐱)f(\tau ,𝐱-{𝐞}_{u})+\\ +\mu_{\text{𝓅𝒽}(u)}(\tau ,𝐱)f(\tau \cup \{u(A_{u}(\tau )+1)\},(𝐱,1))-(a_{\text{𝓅𝒽}(u)}(\tau ,𝐱)+b_{\text{𝓅𝒽}(u)}(\tau ,𝐱)+\mu_{\text{𝓅𝒽}(u)}(\tau ,𝐱))f(\tau ,𝐱)]x_{u} \end{array}$$ (8)

The size dependence of the process is encoded within the parameters $a_{\text{𝓅𝒽}(u)}(\tau ,𝐱),b_{\text{𝓅𝒽}(u)}(\tau ,𝐱)$ and $\mu_{\text{𝓅𝒽}(u)}(\tau ,𝐱)$ that depend both on time τ and on the state of the process **x**. Let us now illustrate the choices made for those parameters and the strict connection to the phenotype.

**The phenotype as the model parameters manager** At a high level, the stochastic process defined by the generator in Eq. (8) relies on three fundamental rates: those governing growth, $a_{\text{𝓅𝒽}(u)}(\tau ,𝐱)$ and $b_{\text{𝓅𝒽}(u)}(\tau ,𝐱)$, and the rate describing mutagenesis, $\mu_{\text{𝓅𝒽}(u)}(\tau ,𝐱)$. However, our framework establishes a deep structural link between these terms through the phenotype and the capabilities acquired by the associated clone.

In order to account for overall resource limitations and for different capabilities to control such resources, we rely on the competitive Lotka Volterra model for any number of species. A detailed analysis of this kind of models is presented in S1 Appendix. In particular we decide to represent the global growth rate of the clone, which is given by $a_{\text{𝓅𝒽}(u)}(\tau ,𝐱)+\mu_{\text{𝓅𝒽}(u)}(\tau ,𝐱)-b_{\text{𝓅𝒽}(u)}(\tau ,𝐱)$, as described in Eq. (S1.3) in S1 Appendix. As a result we have


$$\begin{array}{c} a_{\text{𝓅𝒽}(u)}(\tau ,𝐱)+\mu_{\text{𝓅𝒽}(u)}(\tau ,𝐱)-b_{\text{𝓅𝒽}(u)}(\tau ,𝐱)=\lambda_{\text{𝓅𝒽}(u)}\left(1-\frac{x_{u}+\sum_{\begin{array}{c} v\in \tau ,\\ v\text{≠}u \end{array}}\alpha_{\text{𝓅𝒽}(u)\text{𝓅𝒽}(v)}x_{v}}{k_{\text{𝓅𝒽}(u)}}\right), \end{array}$$ (9)

where $\lambda_{\text{𝓅𝒽}(u)}$, $\alpha_{\text{𝓅𝒽}(u)\text{𝓅𝒽}(v)}$, and $k_{\text{𝓅𝒽}(u)}$ are parameters depending solely on the phenotype of population *u* (and not on the population size) which represent respectively a global maximal growth rate λ, the carrying capacity *k* (as the maximum number of cells the system can sustain) and the interaction terms α’s. The coefficients $\alpha_{\text{𝓅𝒽}(u)\text{𝓅𝒽}(v)}$ dictates the nature of the interplay between the clones with phenotypes 𝓅𝒽(u) and 𝓅𝒽(v).

To refine this system, we decompose the mutational rate $\mu_{\text{𝓅𝒽}(u)}(\tau ,𝐱)$ into two components: the expected number of mutations per cell division, $m_{\text{𝓅𝒽}(u)}$, and the frequency of divisions per unit of time. Notably, this frequency is exactly the birth rate $a_{\text{𝓅𝒽}(u)}(\tau ,𝐱)$, leading to the definition:


$$\begin{array}{c} \mu_{\text{𝓅𝒽}(u)}(\tau ,𝐱):=m_{\text{𝓅𝒽}(u)}\cdot a_{\text{𝓅𝒽}(u)}(\tau ,𝐱). \end{array}$$

Furthermore, we set the death rate $b_{\text{𝓅𝒽}(u)}$ to the constant value equal to 1, which is not only convenient, but also it gives a natural interpretation of the simulation’s time unit: if the death rate is equal to one, then the time unit is the expected life time of a cell of the tissue under analysis, see [35]. The system of equations governing our parameters becomes:


$$\begin{array}{c} \left\{ \begin{array}{c} b_{\text{𝓅𝒽}(u)}(\tau ,𝐱)=1\\ a_{\text{𝓅𝒽}(u)}(\tau ,𝐱)+\mu_{\text{𝓅𝒽}(u)}(\tau ,𝐱)-b_{\text{𝓅𝒽}(u)}(\tau ,𝐱)=\lambda_{\text{𝓅𝒽}(u)}\left(1-\frac{x_{u}+\sum_{v\text{≠}u}\alpha_{\text{𝓅𝒽}(u)\text{𝓅𝒽}(v)}x_{v}}{k_{\text{𝓅𝒽}(u)}}\right)\\ \mu_{\text{𝓅𝒽}(u)}(\tau ,𝐱)=m_{\text{𝓅𝒽}(u)}\cdot a_{\text{𝓅𝒽}(u)}(\tau ,𝐱) \end{array}\right. \end{array}$$

By solving this system, we can express the birth and mutation rates explicitly:


$$\begin{array}{c} \left\{ \begin{array}{c} b_{\text{𝓅𝒽}(u)}(\tau ,𝐱)=1\\ a_{\text{𝓅𝒽}(u)}(\tau ,𝐱)=\frac{1}{m_{\text{𝓅𝒽}(u)}+1}\left[\lambda_{\text{𝓅𝒽}(u)}\left(1-\frac{x_{u}+\sum_{v\in \tau ,v\text{≠}u}\alpha_{\text{𝓅𝒽}(u)\text{𝓅𝒽}(v)}x_{v}}{k_{\text{𝓅𝒽}(u)}}\right)+1\right]\\ \mu_{\text{𝓅𝒽}(u)}(\tau ,𝐱)=\frac{m_{\text{𝓅𝒽}(u)}}{m_{\text{𝓅𝒽}(u)}+1}\left[\lambda_{\text{𝓅𝒽}(u)}\left(1-\frac{x_{u}+\sum_{v\in \tau ,v\text{≠}u}\alpha_{\text{𝓅𝒽}(u)\text{𝓅𝒽}(v)}x_{v}}{k_{\text{𝓅𝒽}(u)}}\right)+1\right] \end{array}\right. \end{array}$$ (10)

This reduction leaves us with four actual parameters that drive the evolution of the tumor and that are independent from the state of the process: the proliferative advantage $\lambda_{\text{𝓅𝒽}(u)}$, the carrying capacity $k_{\text{𝓅𝒽}(u)}$, the interaction coefficients $\alpha_{\text{𝓅𝒽}(u)\text{𝓅𝒽}(v)}$, and the mutational parameter $m_{\text{𝓅𝒽}(u)}$. As previously underlined, these rules depend solely on the phenotype. However, individual functional events do not affect all parameters equally. We now detail how specific categories of functional effects map onto these four parameters.

*Proliferative Advantage*. A functional event of type 1-Deregulation of the proliferation program is characterized by a single parameter $s_{i}$, denoting the *proliferative advantage* conferred by that specific mutation. In accordance with the principle of clonal evolution, these effects are assumed to be summative as they accumulate. Consequently, by isolating the growth-related events from the phenotype,


$$\begin{array}{c} F_{growth}:=\{f_{i}\in F:F_{i}=1\}, \end{array}$$

we define the total proliferative advantage as:


$$\begin{array}{c} \lambda_{\text{𝓅𝒽}(u)}:=\sum_{f_{i}\in F_{growth}\cap \text{𝓅𝒽}(u)}s_{i}. \end{array}$$ (11)

Crucially, if a cell has not acquired any mutations altering cell cycle regulation, its growth rate remains null. This accurately reflects the natural state of healthy tissue, where cell birth and death are in a perfect, homeostatic balance.

*Mutation Rate*. While healthy cells have a balanced growth rate, they do not have a null mutation rate; DNA replication is inherently prone to occasional errors. We therefore define a baseline mutational parameter $m_{base}$. Empirical data suggest the somatic mutation rate in normal human cells is approximately $1\cdot 10^{-9}$ to $5\cdot 10^{-9}$ per base pair per division [50,51]. Functional events of type 2-Mutation burden augmentation serve to amplify this baseline. For each such event $f_{i}$ in


$$\begin{array}{c} F_{mut}:=\{f_{i}\in F:F_{i}=2\}, \end{array}$$

we define a *mutational amplificator factor* $m_{i}$. The cumulative mutational parameter for the phenotype is then:


$$\begin{array}{c} m_{\text{𝓅𝒽}(u)}:=\left[\prod_{f_{i}\in F_{mut}\cap \text{𝓅𝒽}(u)}m_{i}\right]\cdot m_{base}\cdot N_{bp}, \end{array}$$ (12)

where $N_{bp}$ represents the number of base pairs under analysis. This term is highly adaptable; it can represent the entire genome or be scaled to match the coverage of a specific sequencing panel when comparing synthetic data to clinical results.

*Ecological Interaction*. Because tumor clones share the same physical microenvironment, they naturally compete for resources, establishing a baseline interaction of $\alpha_{\text{𝓅𝒽}(u)\text{𝓅𝒽}(v)}=\alpha_{\text{𝓅𝒽}(v)\text{𝓅𝒽}(u)}=1$. The parameter $\alpha_{\text{𝓅𝒽}(u)\text{𝓅𝒽}(v)}$ represents the “ecological weight” that population *v* exerts on the growth limitation of population *u*. Managing independent interaction parameters for every possible pair of populations is computationally and biologically impractical. Instead, we derive these values from the “ecological power” of a population’s mutations. Each type 4-Resource control functional effect $f_{i}$ in


$$\begin{array}{c} F_{res}:=\{f_{i}\in F:F_{i}=4\} \end{array}$$

is characterized by two values: a *susceptibility index* $\nu_{i}$ and an *offensive score* $\eta_{i}$:

$\nu_{i}$ (susceptibility index) measures how strongly a population is affected by interactions with another: it can be interpreted as a relative weight to be applied to the elements of the other population to get the effect on the reference population. Hence, if positive but smaller than one, it indicates that the elements of the other population subtract resources shared with the reference population only partially, while if larger than one, it accounts for a substantial removal of resources. If negative, it describes a beneficial interaction in which the other population supplies resources necessary for the focal population.

$\eta_{i}$ (offensive score) quantifies the reference population’s impact on the other: it can be interpreted as a relative weight to be applied to the elements of the reference population to get the effect on the other population. Hence, if positive but smaller than one, it indicates that the elements of the reference population subtract resources shared with the other population only partially, while if larger than one, it accounts for a substantial removal of resources. If negative, it describes a beneficial interaction in which the reference population supplies resources necessary for the other population.

From these indices, we derive the interaction coefficient $\alpha_{\text{𝓅𝒽}(u)\text{𝓅𝒽}(v)}$ as:


$$\begin{array}{c} \alpha_{\text{𝓅𝒽}(u)\text{𝓅𝒽}(v)}:=\sum_{f_{i}\in F_{res}\cap \text{𝓅𝒽}(u)⧵\text{𝓅𝒽}(v)}(\nu_{i}-1)+\sum_{f_{i}\in F_{res}\cap \text{𝓅𝒽}(v)⧵\text{𝓅𝒽}(u)}(\eta_{i}-1)+1. \end{array}$$ (13)

This additive formula treats 1 as the neutral element of competition. It balances the “offensive score” of mutations unique to population *v* against the “susceptibility index” of mutations unique to *u*. If $X_{u}$ has no vulnerability alterations ($\nu_{i}=1$), the interaction depends entirely on the offensive score of $X_{v}$, and vice versa, ensuring the model remains consistent with common-sense ecological constraints.

*Carrying capacity*. Finally, the carrying capacity $k_{\text{𝓅𝒽}(u)}$ is governed by functional events of type 3-Limit evasion. These mutations allow a clone to expand beyond the initial constraints of the environment, whether by physically finding more space, gathering resources more aggressively, or utilizing them more efficiently. For each such event $f_{i}$ in


$$\begin{array}{c} F_{lim}:=\{f_{i}\in F:F_{i}=3\}, \end{array}$$

we define a parameter $k_{i}$ representing the *additional space* provided. Starting from a baseline capacity $k_{base}$ for wild-type clones, the carrying capacity is:


$$\begin{array}{c} k_{\text{𝓅𝒽}(u)}:=k_{base}+\sum_{f_{i}\in F_{lim}\cap \text{𝓅𝒽}(u)}k_{i}. \end{array}$$ (14)

It is important to distinguish between type 4-Resource control and type 3-Limit evasion functional effects. While both help a tumor overcome growth limitations related to space constraints and resources exhaustion, they operate through different mechanisms. The type 4 functional effects contribute in regulating how existing resources are internally allocated among clones through competition or cooperation. In contrast, type 3 functional events induce an expansion mechanism, allowing specific clones to access previously unavailable nutrients or niches, thereby providing an exclusive advantage that alters the tumor’s global resource dynamics.

### The simulation algorithm

At each time, the process $(X_{u}(t){)}_{u\in N(t)}$ is a multi-dimensional random variable with dimension equal to the number of populations with at least one alive cell, |*N*(*t*)|. The evolution of each established population follows a size-dependen*t* branching process that can give birth to the first cell of a new emerging population with a new genotype that includes a newly acquired mutational event. The simulation algorithm is a discrete-time approximation of the process $(X_{u}(t){)}_{u\in N(t)}$ which updates all of its components: the abundances at the next time point $X_{u}(t+\Delta )$ for all u∈N(t), the set of established populations at time *t*, and the abundances of the emerging populations, with birth time $\sigma_{u}$ between *t* and t+Δ. Please note that both the latter upda*t*es are needed to correctly refresh the process *X*(*t*), see Eq. (7). The update of the abundances of the established populations at time *t* is computed before the update of the emerging populations as the result of the former is necessary for the calculation of the latter.

A graphical representation of one single simulation step is reported in Fig A in S2 Appendix. Moreover, a study for the choice of the largest timestep Δ that controls the approximation error is given, see S2 Appendix. The pseudocode for the full algorithm is included in S2 Appendix. In the following, we are describing the main simulation steps.

**Established populations update from time *t* to time** t+Δ. Once the birth parameters have been adjusted using Eq. (10) by substituting the population sizes with the values calculated at the previous time step, $X_{u}(t)$ for any genotype *u* emerged by time *t*, u∈N(t), the evolution of population sizes is simulated freezing the birth and death parame*t*ers, as if they were constant for the time interval Δ. The distribution of the number of individuals at any time t+Δ is well known for classical birth and death processes. In particular, we have that, by temporal homogeneity and setting $P_{ij}(\Delta )=\mathbb{P}(X_{u}(\Delta )=j|X_{u}(0)=i)$, the following distribution can be derived, see [52]:


$$\begin{array}{c} \left\{ \begin{array}{c} P_{10}(\Delta )=\alpha \\ P_{1n}(\Delta )=(1-\alpha )(1-\beta {)}^{n-1}\beta , \end{array}\right. \end{array}$$ (15)

where


$$\begin{array}{c} \alpha :=\frac{b_{ph(u)}-b_{ph(u)}\cdot e^{-\lambda \Delta }}{a_{ph(u)}-b_{ph(u)}\cdot e^{-\lambda \Delta }},\;\beta :=\frac{a_{ph(u)}-a_{ph(u)}\cdot e^{-\lambda \Delta }}{a_{ph(u)}-b_{ph(u)}\cdot e^{-\lambda \Delta }}. \end{array}$$ (16)

The Embedding theorem, see [53], states that for any choice of discretization step Δ, a birth and death process evaluated at time Δ is a Galton-Watson process with offspring distribution defined by $P_{1n}(\Delta )$ given in Eq. (15). This implies that for simulating $X_{u}(t+\Delta )$, we can employ multinomial sampling with frequencies of occurrences $P_{1n}(\Delta )$ for n=0,…,M truncated for sufficiently large (negligible probability) *M*. Since each individual reproduces independently from the others we can consider the evolution of $X_{u}(t)$ cells as the replication of $X_{u}(t)$ trials of the same experiment whose distribution is given in Eq. (15); the simulation will thus be founded upon:

1. Sample a vector **v** from a multinomial distribution with parameters $X_{u}(t)$ and $\left(P_{10}(\Delta ),P_{11}(\Delta ),\ldots ,P_{1M}(\Delta )\right)$.
2. Update $X_{u}(t+\Delta )=𝐯\cdot (0,1,2,\ldots ,M)$.

The simulation error here is driven by the freezing of the birth and death parameters at the beginning of the time step *t*. Taking inspiration from the well-known τ-leaping simulation algorithm [54], it is possible to consider a limitation of the time step Δ chosen in such a way that the expected state change is small, thus it does not excessively affect the parameters. See S2 Appendix for a complete study of the simulation error.

**Birth of new populations.** As reported in Eq. (6), the number of daughter populations with parent *u*, born by time *t*, conditional on ξ given in Eq. (5), follows a Poisson distribution. This means that the probability of having *k* daughter populations from *u*, in the time interval [t,t+Δ) is given by


$$\begin{array}{c} P_{k}(t)=\frac{{\left(\mu_{ph(u)}\int_{t}^{t+\Delta }X_{u}(s)ds\right)}^{k}}{k!}e^{-\mu_{ph(u)}\int_{t}^{t+\Delta }X_{u}(s)ds}. \end{array}$$ (17)

To sample from the correct distribution, it would be required to know $X_{u}(s)$ for any s∈[t,t+Δ]. Such information is not available, as we simulate the abundances only at the discrete instants *t* and t+Δ. We proceed by approximating the integral with a simple trapezoidal rule. Hence for each population *u*, the number of daugh*t*ers with a new mutation (i.e., the number of new populations) are sampled from a Poisson distribution with parameter $\Delta \cdot [X_{u}(t)+X_{u}(t+\Delta )]/2$. Each time a new population appears, the new characterizing mutation is named with a unique identifier. The genotype of the new population is then derived concatenating the mother’s genotype with the new unique mutation identifier *v* = *uj*. To each mutational event, a functional capability is associated by sampling a functional event from the functional event list, see Eq. (1), according to an occurrence distribution $𝐫=(r_{1},\ldots ,r_{I})$. that represents the probability that a random mutational event will result in a given phenotypic effect. For each new population the phenotype is then calculated following Eq. (3).

A user interface developed in Next.js allows running the simulator through a configurable environment, where users can set model parameters and execute single simulations. The interface, available together with the source code on GitHub (https://github.com/qBioTurin/Insite_Interface), provides access to the simulation engine through an interactive and user-friendly workflow, returning the full detailed output of each run, including all cell-level information and derived summaries. To ensure full reproducibility and facilitate direct access to the underlying computational framework, we also provide a separate GitHub repository, Insite (https://github.com/qBioTurin/Insite), containing the complete simulation and analysis R package upon which the interface is built, together with example scripts and utilities for reproducing the experiments presented in both the main manuscript and the Supplementary Materials. Run times for a set of experiments are reported in Fig L in S3 Appendix.

### Methods for downstream analysis of simulated tumors

The simulator tracks tumor evolution at single clone resolution, recording for each cell its genotype and phenotype. This information allows for the reconstruction of the complete temporal dynamics and clonal architecture of the synthetic tumor. Several quantitative representations can then be derived at different levels of aggregation.

**Fig 2:**
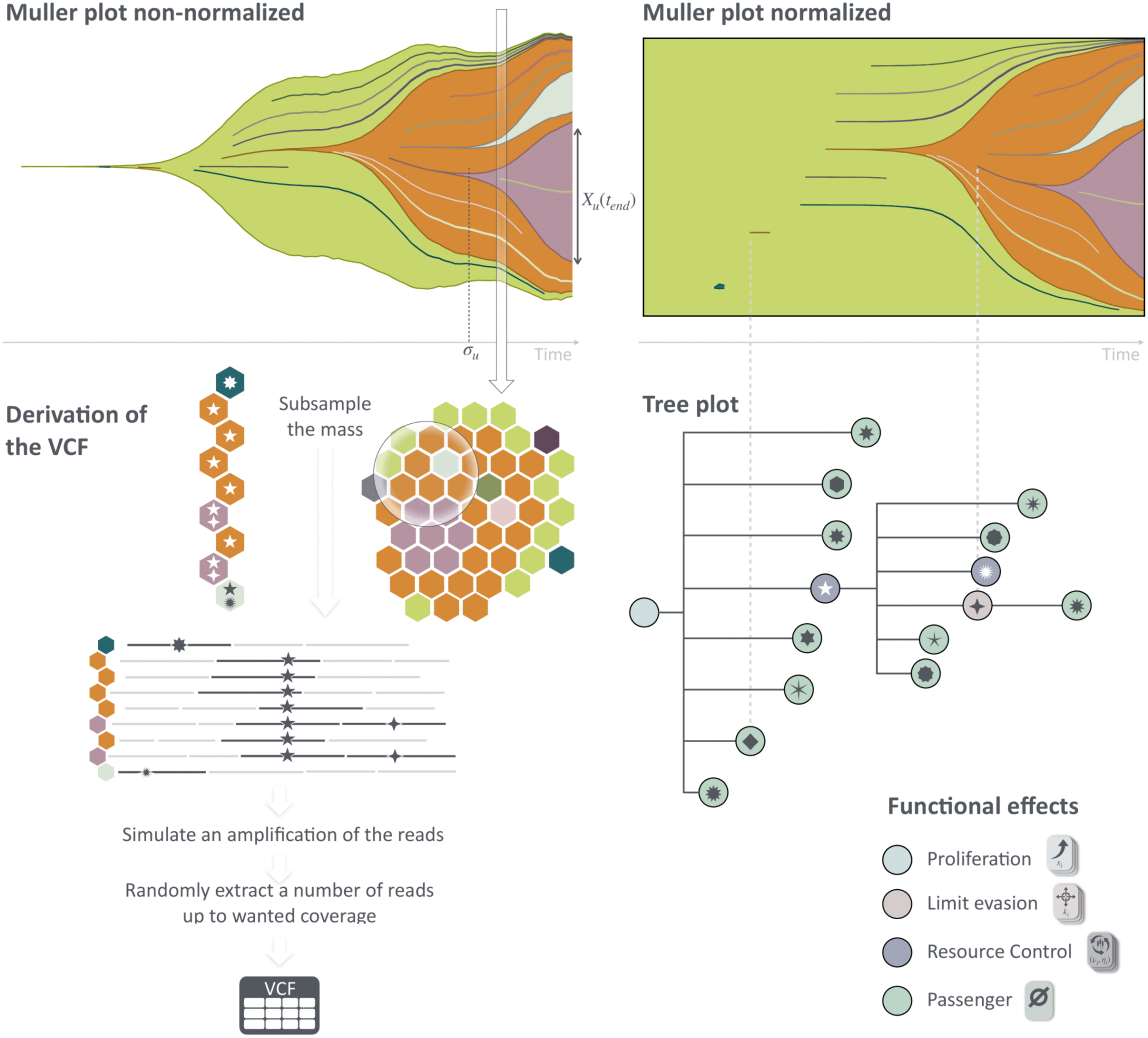
Visualization and re-elaboration of simulated tumors. Muller plots (top row) provide a comprehensive view of clonal dynamics. Tree plots (bottom-right) summarize the evolutionary history, with a color code indicating the functional effects associated to the point mutations. A tumor sample is sequenced (bottom-left), variant calling is performed and a synthetic VCF file is produced.

At the most detailed level, clonal dynamics can be visualized through Muller plots or through evolutionary trees, which depict the temporal expansion of subclones and the mutational relationships among them. Moreover, the collection of accumulated mutations can be organized by mimicking a bulk sequencing and a variant calling procedure. The final output of the synthetic sequencing and variant calling is represented as a VCF (Variant Call Format) file. Each mutation is assigned to distinct reads that are randomly amplified and downsampled to reach a target coverage, optionally drawn from empirical distributions. See Fig 2 for a comprehensive view of the simulation outputs. The Muller plot, tree plot, and synthetic VCF files are shown in the user interface as well.

**Fig 3:**
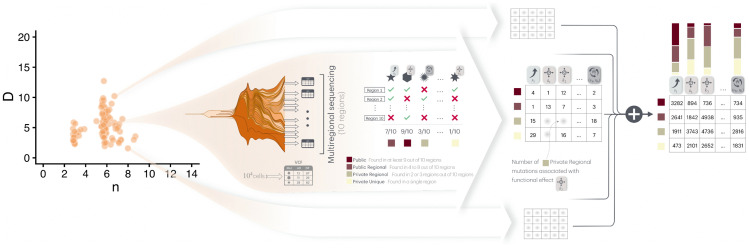
Procedure of multiregional sequencing used for the classification of mutations based on the spatial pattern of occurrence. For each synthetic tumor, we extract the synthetic VCF, over 10 non-overlapping regions. Single mutations are classified as *Public*, *Private Unique*, *Public Regional* and *Private Regional* depending on the frequency of occurrence. The two-way contingency table of the joint distribution of the type of functional event associated to the mutation and the Public/Private spatial pattern is derived. The contingency tables from the single tumors are summed up to a single contingency table, which shows how different types of functional events (functional capabilities) are associated to distinct spatial patterns. The overall contingency table can be plotted as grouped barplot as well.

We developed a multiregional sequencing of the synthetic tumors as well, see Fig 3. For each synthetic tumor, we repeated (10 times) the procedure described in Fig 2 to extract a synthetic VCF, over non-overlapping regions made of 10^4^ cells. Relying on the individual mutation IDs, mutations detected in all (or all but one) sequenced regions were classified as *Public*; mutations detected in a single sequenced region as *Private Unique*; and mutations shared by some but not all sequenced regions as *Regional*, further subdivided into *Public Regional* (present in 4−8 regions) and *Private Regional* (present in 2−3 regions). At this point, from each synthetic tumor, we have obtained a list of point mutations with associated functional event and classification into Public/Private spatial pattern. We group the point mutations by functional event and we get the two-way contingency table of the joint distribution of the functional event and the Public/Private spatial pattern. The contingency tables from the single tumors were finally summed up to a single contingency table, which shows how different functional events (functional capabilities) are associated to distinct spatial patterns.

At a more abstract level, the evolutionary outcome of each simulation can be summarized by two quantitative indices proposed in [41]: *clonal diversity* $D(t)={\left[\sum_{u\in N(t)}{\left(\frac{x_{u}}{\sum_{v\in N(t)}x_{v}}\right)}^{2}\right]}^{-1}$, reflecting the average number of clones of the same size, and *clonal nesting* $n(t)=\sum_{u\in N(t)}|u|\cdot \frac{x_{u}}{\sum_{v\in N(t)}x_{v}}$, representing the average depth of the clonal tree, which corresponds to the average number of mutations per cell. While Muller plots, phylogenies, and VCFs provide detailed insights into individual simulations, the (*n*,*D*) metrics offer a compact quantitative summary suitable for large-scale comparisons and model validation against empirical data by summarizing the information into a point of a bidimensional plane. Furthermore, these metrics are robust to the addition or removal of rare clones as discussed by the authors, and well established in the literature regarding mathematical modelling for the validation, see [42].

We leveraged both the detailed clonal reconstructions and their (*n*,*D*) summaries to investigate which evolutionary paradigms naturally emerge from different combinations of functional effects and to validate the model against real data.

## Results

### Exploration of evolutionary paradigms

A systematic exploration of the model’s parameter space has first been conducted by isolating one type of functional event at a time, allowing the role of each functional mechanism to be disentangled across a broad range of configurations, see S3 Appendix. That analysis revealed few robust findings: 1. a clear distinction between the behavior observed during the tumor’s initial expansive phase, when resources and space are still available, and the later stable phase, when those resources and space have been depleted; 2. the possibility that passenger mutations (type 0-Null effect functional events) give rise to clones that reach detectable sizes when they arise early in the expansive phase; 3. the fact that proliferative deregulation (type 1-Deregulation of the proliferation program functional events) give rise to non-neutral dynamics only during this early phase, showing larger survival probability and expansion potential. However, if the type 1 functional event is acquired later in the stable phase, where resources and space are no more available, the clone is not able to increase its size and become detectable. Taken separately, these observations do not yet establish to which extent each different mechanism is responsible for the emergence of sustained intratumor heterogeneity.

**Fig 4:**
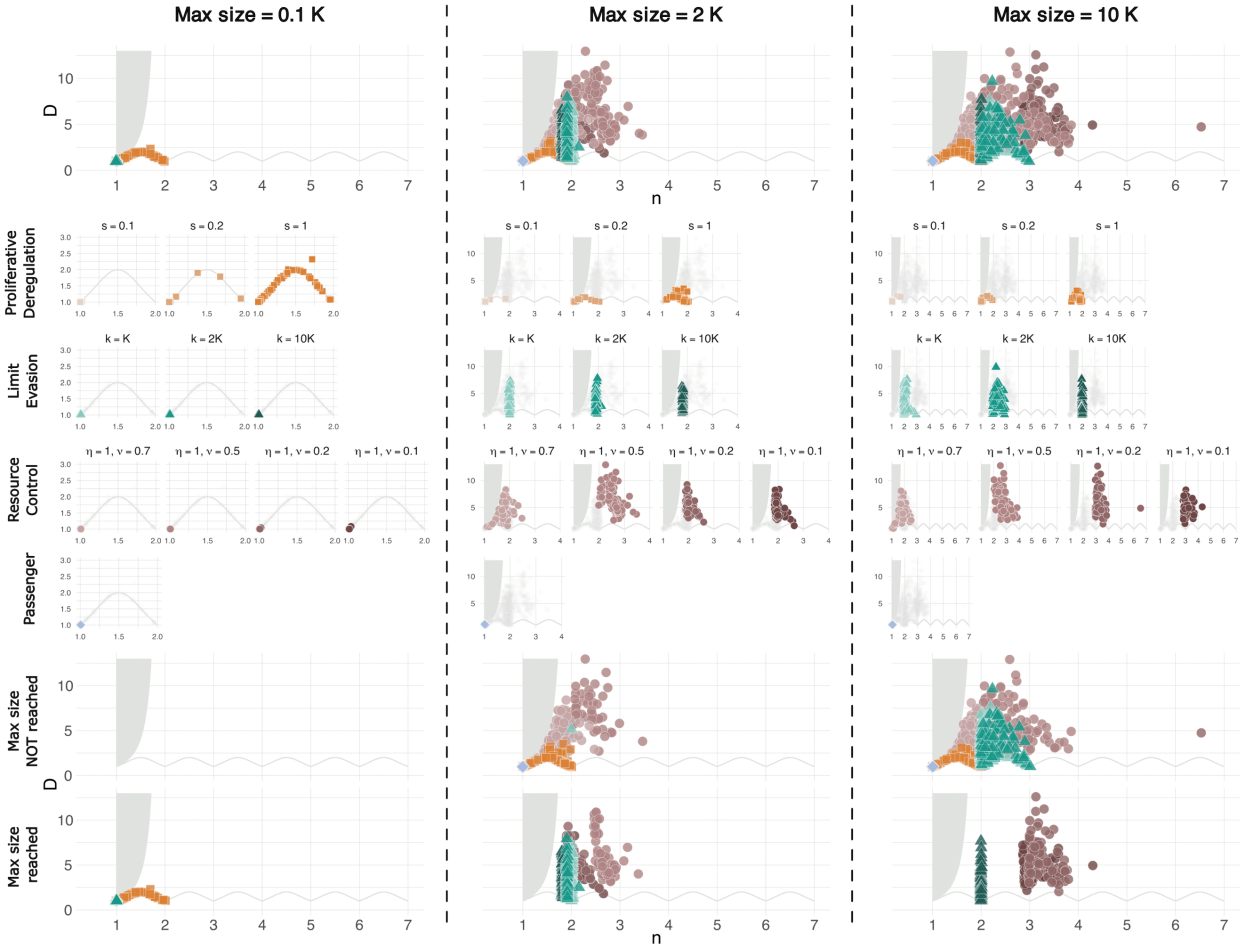
Simulated tumors on the (*n*,*D*) plane. Each point represents a single independent simulation run; the shape indicates the type of primary mechanism included, and the colour denotes the specific parameter configuration. Simulations start from a single cell already carrying a mutation associated to type 1-Deregulation of the proliferation program primary mechanism with parameter $s_{base}=0.1$. The baseline mutation rate is equal to $m_{base}=2.66\cdot 10^{-9}$ (across $N_{bp}=1000$ bp), and carrying capacity is fixed at *K* = 10^6^. In each run, the functional event list *F*, see Eq. 1, consists of 6 identical events of the same type, chosen among type 1-Deregulation of the proliferation program (**squares**), type 3-Limit evasion (**triangles**), type 4-Resource control (**circles**), type 0-Null effect (**diamonds**). Different choices of the parameters have been explored: s∈{0.1,0.2,1.0} (**light**, **medium** and **dark** orange), k∈{K,2K,10K} (**light**, **medium** and **dark** green) and η=1, ν∈{0.7,0.5,0.2,0.1} (from **lightest to darkest** mauve). For each configuration of the parameters, a total of 100 simulations have been run. Simulations are stopped either upon reaching the maximum size *M* or the maximum time equal to 5 years. In the **left column** $M=\frac{1}{10}K$, corresponding to an early stopping during the expansive phase of the tumor. In the **central column** *M* = 2*K*, corresponding to a stopping during an early stable phase of the tumor. In the **left column** *M* = 10*K*, corresponding to a stopping during a late stable phase of the tumor. **Top row**: all runs are shown together in the same (*n*,*D*) plane, coloured by parameter values and shaped by mechanism type. **Middle row**: runs are stratified to isolate each mechanism and parameter configuration. **Bottom row**: runs are separated based on whether the maximum size *M* was reached before the maximum time.

To address this, we turn to the integrated experiment summarized in Fig 4, which condenses many simulated tumors into a unified (*n*,*D*) representation. In this setting, each simulated tumor can accumulate mutations that are associated to six functional events maximum, all of the same type and with identical parameters. This design enables a controlled, differential comparison between functional mechanisms while still accounting for cumulative effects of repeated alterations of the same type. Each point in Fig 4 therefore represents the outcome of a full stochastic trajectory under a specific mechanism and parameter choice.

Simulations are stopped either upon reaching the maximum size *M* or the maximum time equal to 5 years. Three different values of *M* have been explored. A value smaller than the carrying capacity *K*, namely $M=\frac{1}{10}K$, which corresponds to an early stopping during the *expansive* phase of the tumor. Two values larger than the carrying capacity, *M* = 2*K* and *M* = 10*K*, corresponding to a stopping during an *early stable* phase of the tumor and a *late stable* phase of the tumor, respectively. A first, clear separation emerges between the behaviors exhibited at the expansive phase and at the two stable phases. In the expansive regime, only the accumulation of mutations associated to type 1-Deregulation of the proliferation program is able to drive any appreciable departure from the baseline point (*n* = 1, *D* = 1). As predicted by [41], if the selective advantage *s* is sufficiently large, the evolution follows sequential selective sweeps and (dark) orange squares align on the light gray curves calculated in [41]. Diversity stays low and the number of dominant clones never exceeds n≈2, even when the system is observed across all replicates. In this regime, the framework naturally recovers *punctuated*-like dynamics: early proliferative events generate rapid expansions, after which the system quickly saturates and no further diversification occurs. This picture changes radically in the stable regime. Once resource saturation and ecological constraints become relevant, proliferative deregulation (type 1) loses explanatory power: its trajectories remain confined to the same low-diversity sweep structure observed in the early phase. In contrast, mechanisms based on resource control (type 4) and limit evasion (type 3) become the dominant drivers of evolution. They progressively occupy the entire (*n*,*D*) plane, generating both high-diversity configurations and complex branching patterns. The transition is not gradual in terms of qualitative behaviour: it is a regime shift in which the relevant explanatory mechanisms are different. Passenger-only configurations (type 0), by contrast, remain concentrated at the baseline point (*n* = 1, *D* = 1) across all phases, reflecting the fact that the accumulation of neutral mutations alone does not generate dominant clones at the population scale.

**Fig 5:**
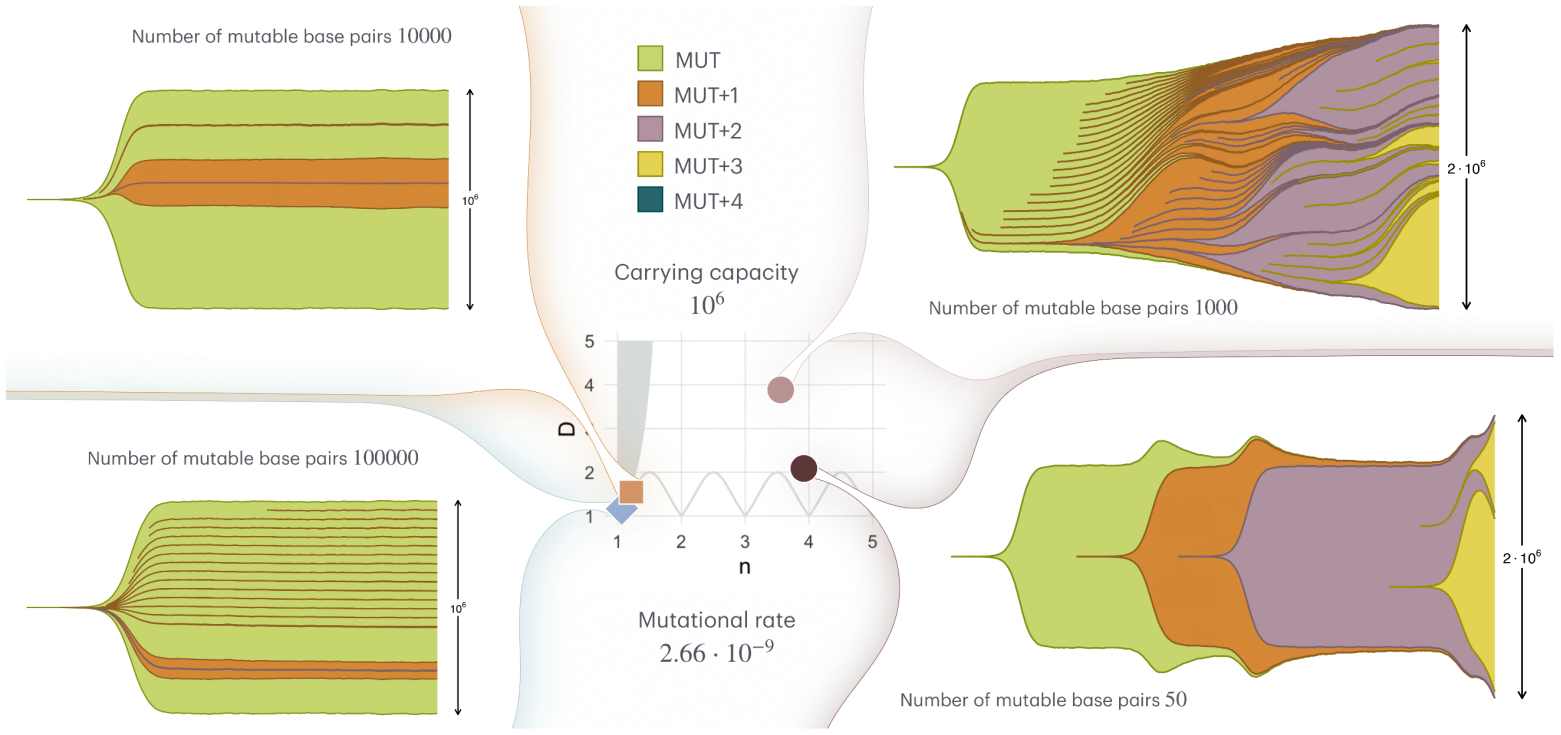
Examples of clonal architectures observed in the experiments. Four dots in the (*n*,*D*) plane (single simulated tumors) are illustrated through the Muller plots, coloured according to the number of mutational events associated with the clone. MUT represents the first oncogenic mutation originating the tumor. The initiating cell has a single mutation with associated functional effect of type 1-Deregulation of the proliferation program defined by the parameter $s_{base}=0.1$, see Eq. (11). The **bottom-left panel** reports an experiment in which only type 0-Null effect (passenger) functional effects can be associated to mutations. The **top-left panel** reports an experiment in which only single type 1-Deregulation of the proliferation program functional effects can be associated to mutations (with parameter *s* = 0.1). The **top-right panel** reports an experiment in which six type 4-Resource control functional effects maximum can be associated to mutations, (with identical parameters (ν=0.7,η=1), see Eq. (13)). The **lower-right panel** reports an analogous experiment with six type 4-Resource control functional effects maximum, but with parameters (ν=0.1,η=1). Other parameters are: baseline mutational rate $m_{base}=2.66\cdot 10^{-9}$ per base pair, number of mutable base pairs $N_{bp}=10000$ (upper-left panel), $N_{bp}=100000$ (lower-left panel), $N_{bp}=50$ (lower-right panel), $N_{bp}=1000$ (upper-right panel), carrying capacity *K* = 10^6^ cells.

The zoomed examples represented in Fig 5 illustrate the microscopic origin of these macroscopic patterns. Under pure passenger accumulation (type 0-Null effect functional events), most subclones remain extremely small, with only rare events producing detectable expansions, an example of this uncommon occurrence is visible in Fig 5 bottom-left panel. When such expansions occur, they are not driven by any intrinsic advantage of the clone, but by a purely stochastic fluctuation: it is only by chance that lineages in the surrounding population go extinct, allowing the neutral clone to drift to an unexpectedly large prevalence that is then preserved once carrying capacity is reached. This explains why these events are observed only for mutations that arise very early, during the expansive phase of the tumor evolution. By contrast, proliferative deregulation mutations (type 1-Deregulation of the proliferation program) exploit a genuine selective advantage during this same phase, which makes them consistently more likely to expand as visible in Fig 5 top-left panel. Once the carrying capacity is reached, however, this advantage becomes ineffective: clonal sizes stabilize, and newly acquired proliferative mutations no longer produce significant expansions. As a result, both neutral-like and proliferative regimes converge to the same thin, sweep-like structures, explaining why they collapse onto the same low-*n* region in Fig 4. In this sense, the framework also recovers signatures typically associated with *neutral evolution*: in [24], the authors already showed that, under neutrality, passenger mutations yield a VAF distribution heavily skewed toward very low frequencies, exactly as we observe in our simulations; however, their conclusion that such patterns imply predominantly neutral evolution has been widely criticized [55–57]. Indeed in our framework low-frequency variants arise not only from passenger mutations (type 0), but also from proliferative deregulation mutations (type 1) acquired in the stable phase, where their selective advantage cannot be expressed.

The key result of the systematic exploration of the tumor evolution under our model is that this degeneracy, caused by the resources exhaustion when the system reaches the carrying capacity, is broken only by ecological interaction terms. In particular, the susceptibility index ν, associated to type 4-Resource control functional effects, acts as the main control knob of the system. Recall that large positive values of the parameter indicate that the population is strongly impaired by competitors, whereas positive values below one indicate a weaker harming effect due to competition. Equivalently, large positive values of the parameter indicate that the population has weak resistance to competitors, whereas positive values below one indicate a stronger response to competition. The sensitivity of the model to this parameter is discussed in details in S3 Appendix, yet it is worth noting that varying this parameter allows for spanning from complete clonal sweeps to fully branched architectures of evolution. When the tumor accumulate, at low rate, mutations associated to type 4-Resource control functional effects with small positive values of ν, the new populations have strong resistance to competition and, given the low rate of mutation, they have the time to increase their sizes by using a larger amount of the limited resources letting the system collapse into near-perfect selective sweeps, see Fig 5 bottom-right panel. Under these conditions, only a single lineage at a time can effectively persist, reproducing dynamics consistent with *linear evolution*. Conversely, for milder resistance (larger susceptibility parameters), multiple lineages can coexist and expand simultaneously, producing architectures such as that in Fig 5 top-right panel, and giving rise to *branching evolution*. These two regimes emerge from continuous parameter variations within the same mechanism, rather than from fundamentally distinct model assumptions.

Type 3-Limit evasion events display a complementary behaviour, strongly coupled to the stopping condition. When simulations terminate upon reaching the given maximum size, tumors tend to exhibit similar clonal nesting values and align vertically along the *D* axis. When simulations are instead time-limited, the points become more dispersed, although always above a minimum threshold in *n*. This reflects the interplay between the additional space introduced by mutations and the imposed size constraint. In the *M* = 2*K* scenario, differences between parameter values are minimal: a single mutation providing an additional carrying capacity (or more) is sufficient to reach the stopping condition, leading to an accumulation of points along the line *n* = 2. By contrast, in the *M* = 10*K* scenario, only mutations conferring large increases in available space remain localized, while weaker effects generate broader distributions. In principle, given sufficient time and repeated events, these trajectories would converge toward discrete vertical structures corresponding to the number of mutations required to reach the imposed size threshold.

Importantly, the high-diversity region of the plane (*n*,*D*) in Fig 4 cannot be populated by tumors with type 1-Deregulation of the proliferation program or type 0-Null functional capabilities alone: they are structurally confined to low-*n* and low-*D* configurations. The full experiment therefore isolates type 4-Resources control and type 3-Limit evasion as the only mechanisms capable of generating sustained intratumor heterogeneity in the stable regime, as proliferative deregulation (type 1) only governs early-time expansion dynamics.

### Validation against empirical data

**Fig 6:**
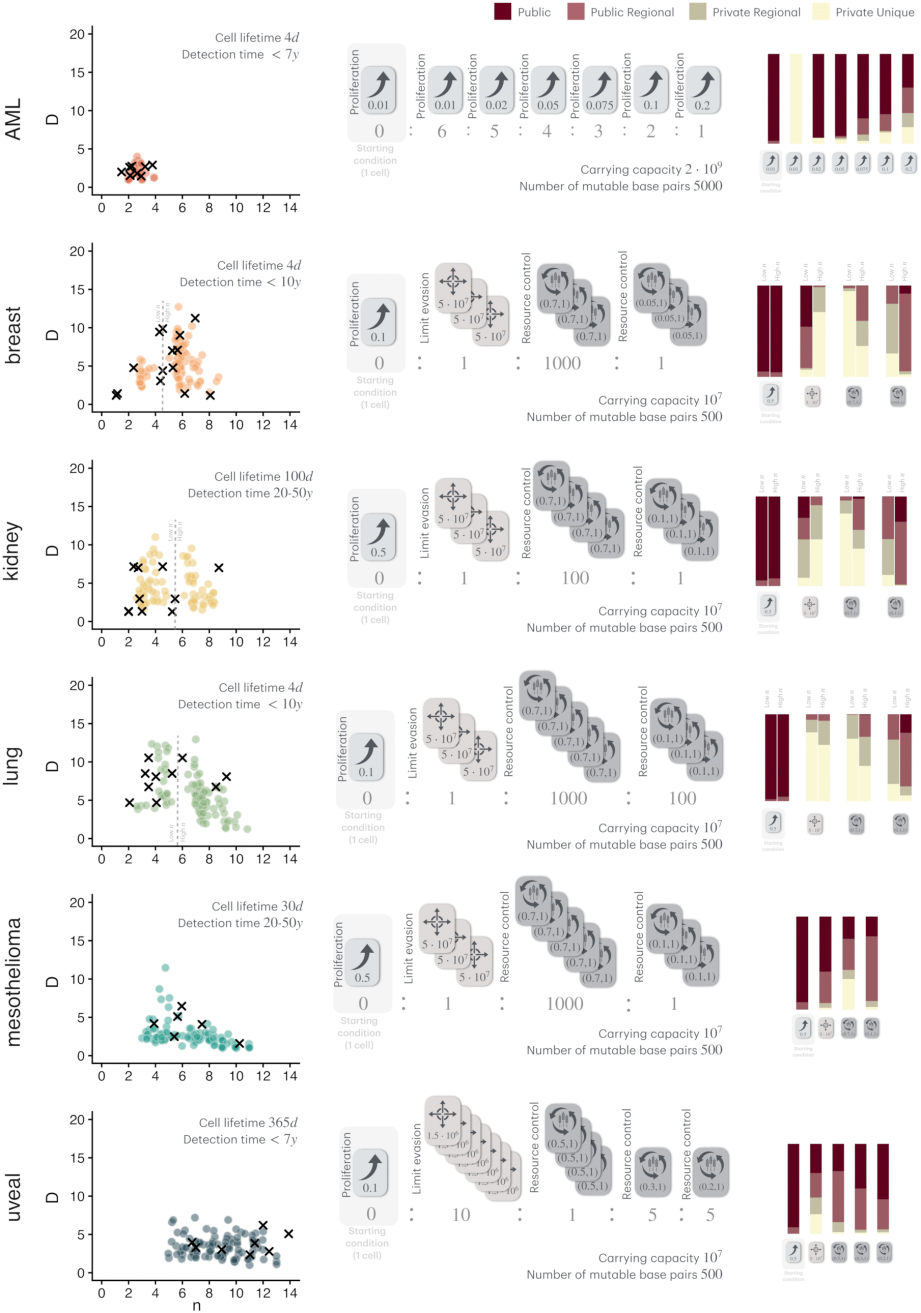
On the left column, plots in the (*n*,*D*) plane for 6 cancer types. Black crosses are the (*n*,*D*) values extracted from real tumors in [41]. Coloured dots are the (*n*,*D*) values extracted from synthetic tumors simulated according to our model with parameters values equal to the maximum likelihood parameters estimates. The darker and white-bordered dot in each panel is the centroid of the coloured dots. On the **central column**, the maximum likelihood estimates parameters of the model are represented as **cards** with a symbol corresponding to the type of the primary mechanism associated to the functional event and the values of the parameters. For the sake of readability, the cards are grouped by primary mechanism and parameters values. The name of the primary mechanism is reported on the left and the frequency of association of each card to a newly acquired mutation is reported at the base of each group of cards. The full set of cards is a visualization of the functional event list *F*, see Eq. (1). The light gray shaded card is the phenotype of the initiating cell. On the **right column**, grouped barplots describe the joint distribution of the type of functional effect and the spatial pattern of the associated mutations, see Fig 5. For breast, kidney and lung cancers the barplots are derived separately for tumors with low/high values of *n*.

To evaluate whether the simulated evolutionary trajectories are biologically plausible, we compared them with real tumor data using the *clonal diversity* (*D*) and *clonal nesting* (*n*) indices evaluated for clones above a minimal frequency threshold of 10^−2^. The idea behind this is that different tumor types occupy distinct regions of the (*n*,*D*) space, corresponding to characteristic evolutionary behaviours. Real experimental data were obtained from the GitHub repository accompanying [41], where the evolutionary indices of phylogenetic trees previously inferred from multi-region sequencing and single-cell sequencing have been derived. This procedure has been conducted using data from 7 different studies regarding 6 different tumors: acute myeloid leukaemia [58], clear cell renal cell carcinoma [59], mesothelioma [60], breast cancer [61] and [62], non-small cell lung cancer [63], uveal melanoma and [64]. From each tumor, specific constraints regarding the parameters are derived: for instance, the average lifetime of the cells has been set according to the tissue, they are reported in Fig 6. The basic healthy tissue mutation rate per base pair per cell division has been fixed at $m_{base}=2.66\cdot 10^{-9}$ according to [51]. Simulations have been stopped when the synthetic lesion reaches a detectable size, which in literature has been identified with 1 cm^3^ for solid tumors, except for uveal melanoma, which resides in the eye, thus it becomes perceivable already at 1 mm^3^. The size 1 cm^3^ is usually considered to correspond to 10^9^ cells [65]. Though our model only traces alive cells, which are a fraction of the overall tumor mass. Hence stopping size has been set to 10^8^ for kidney, mesothelioma, breast, and lung, and to 10^7^ for uveal, corresponding to the assumption that 10% of tumor cells are alive. For AML, which is a liquid tumor, a larger limit of 10^9^ cells has been used to set the stopping time. For each cancer type, a realistic, generous range for the detection time has been set according to literature [66–75] and reported in Fig 6.

All the simulations have been assumed to start with a single cell carrying a proliferative advantageous mutation (type 1 primary mechanism), that have been excluded by the later acquirable mutations, setting its relative frequency of occurrence *r*_1_ = 0. Passenger mutations (type 0 primary mechanism) are not included in those experiments. Different configurations of primary mechanisms and of their parameters have been tried, running 100 simulations for each chosen configuration. Then, the (*n*,*D*) metrics have been calculated and each resulting group of 100 points (corresponding to the 100 simulations) derived from the same parameter set θ has been used to construct a 2-dimensional distribution via kernel estimation ${\hat{f}}_{\theta }(n,D)$. The estimated density ${\hat{f}}_{\theta }(n,D)$ is used to compute the likelihood function at the real experimental sample points (the black crosses on the (*n*,*D*) plane), which is equal to $ℒ(\theta )=\prod_{i\in 1,\ldots ,n}{\hat{f}}_{\theta }(n_{i},D_{i})$. A visual representation of the procedure is depicted in Fig M in S3 Appendix. The likelihood value is a good estimation of the goodness of fit of the parameter set, hence the parameters realizing the maximum are chosen and represented in Fig 6. The left column comprises the plots on the (*n*,*D*) plane of the real experimental sample points (black crosses) together with the synthetic points corresponding to the 100 simulations with parameters equal to the maximum likelihood estimates. The central column illustrate such parameters as cards (see the caption of Fig 4 for a detailed explanation of the cards). In the accompanying Fig N in S3 Appendix, for each plot on the (*n*,*D*) plane, the centroid of the synthetic points (emphasized in the scatterplots) has been extracted and its corresponding synthetic tumor is plotted through the Muller plot and the evolutionary tree.

A key distinction emerges between liquid and solid tumors. For AML, the maximum likelihood parameter set consists purely of type 1-Growth-enhancing primary mechanisms, combined with a carrying capacity larger than the stopping size, reflecting the fact that hematologic malignancies can reach detectable sizes without exhausting their environment; here, simple proliferative advantage is sufficient to explain tumor expansion. In contrast, all solid tumors are more likely described by parameter sets that include a carrying capacity smaller than the detection size and acquirable functional events increasing space or resources (type 3-Limit evasion), or exploiting resistance (type 4-Resources control, with susceptibility between 0 and 1 and offensiveness equal to 1). This suggests that the commonly assumed effect of driver mutations as pure growth enhancers may not capture the main mechanisms in solid tumors, where physical constraints and inter-clonal competition play a larger role. Interestingly, once space and resistance are identified as the dominant factors, very different tumor dynamics across solid cancers can be reproduced by only modest changes in parameter values or in the relative frequency of these events. This highlights how subtle shifts in the balance of space acquisition and resistance can generate diverse clonal architectures, reflecting the heterogeneity observed across different tissue types and environmental contexts.

Together, these results validate the simulator as a faithful model of tumor evolution. Its fitted configurations recover the qualitative differences between liquid and solid cancers and reproduce realistic tumor sizes and growth times. Moreover, the fit to empirical data reinforces the conclusion drawn from the sensitivity analysis illustrated in S3 Appendix: in solid tumors, evolutionary dynamics are primarily shaped by resource limitations and by the ability of subclones to evade or withstand ecological constraints, rather than by proliferative advantage alone.

### Synthetic multiregional sequencing from multiple tumors

Following [25], we classified mutations according to their spatial pattern of occurrence and analyzed these patterns in relation to the associated functional events.

To this goal, we mimicked a multiregional sequencing of the synthetic tumors, see Fig 3. The results are shown in Fig 6 right-column, as grouped barplots from the two-way contingency tables. For breast, kidney, and lung cancers, where simulations plotted on the (*n*,*D*) plane revealed a separation along the horizontal axis, we preformed the analysis separately for low-*n* and high-*n* synthetic tumors and compared the resulting frequency distributions.

The results show that AML evolution is solely driven by mutations or structural variations that directly affect proliferative capacity and that have mainly a Public spatial pattern, as expected by a clonal architecture built by subsequent selective sweeps. Interestingly, in breast and kidney cancers, the distribution of the Public/Private spatial pattern differs depending on whether tumors are characterized by a low-*n* or high-*n* number of driver gene mutations. Specifically, the last column of Fig 6 reports the grouped barplots of the joint distribution of the functional event and Public/Private spatial pattern, at the time when the tumor reaches a detectable size (diagnosis time). We see two evolutionary scenarios. In the first scenario, tumors with a low-*n* number of driver mutations are characterized by public spatial patterns associated to functional events with type 3-Limit evasion functional effects. Moreover, we could assume that the mutations responsible for those funtional effects have been acquired during the early phases of cancer progression, see Fig O in S3 Appendix, where the times to diagnosis for the two groups are plotted and the low-*n* group is shown to have significantly smaller times to diagnosis. Notably, the three distinct categories of functional effects can be related to different biological processes involving genes linked to pathways associated with loss of cell adhesion and/or increased efficiency of nutrient usage and survival under hypoxia or starvation. Distinctly, the tumors characterized by a high-*n* number of driver mutations exhibit a different evolutionary pattern. These tumors show a higher prevalence of private spatial patterns related to functional events of type 3-Limit evasion functional events, alongside a more persistent presence of public spatial patterns associated with functiona events of type 4-Resource control functional events. As described by Fig O in S3 Appendix, the high-*n* tumors are associated to larger times to diagnosis, hence to slower growth trends. This suggests that, from a biological perspective, alterations that induce type 4-Resource control functional capabilities may be acquired more slowly over tumor evolution. For these tumors, seven (kidney) and six (breast) distinct functional events within the type 4-Resource-control category are modeled, characterized by different degrees of susceptibility and offensiveness. These events can be interpreted as related to internal resource generation during starvation or stress, support of rapid proliferation and membrane remodeling, or reduced immune metabolic competition, ultimately leading to increased resource availability for cancer cells. A similar analysis was performed for lung cancer, stratifying tumors into groups with low-*n* and high-*n* number of driver mutations. In this case, intratumoral heterogeneity appears to be more pronounced, as more cases result with large *D* values, see Fig 6. The type 3-Limit evasion functional events are mainly Private unique classified, both for the high-*n* and low-*n* groups. This suggests a more complex and heterogeneous evolutionary landscape in lung cancer, where multiple functional strategies may coexist at the time of diagnosis. Finally, in mesothelioma and uveal melanoma, the number of driver mutations is more concentrated within a narrower range and is associated with limited heterogeneity, as reflected by lower values of the heterogeneity parameter, *D*. These tumors are characterized by a generally high proportion of public mutations, broadly distributed across all the biological categories considered, indicating a more homogeneous evolutionary trajectory dominated by shared functional events.

## Discussions

We have designed and implemented a stochastic tumor evolution model that unifies, within a single mathematical framework, the main conceptual approaches currently proposed in the literature. In particular, our model integrates branching process descriptions of cell division and mutational events [13,16,34–37], neutral expansion scenarios as formalized in the Big Bang model [24,25], and ecological perspectives that emphasize environmental limitations and clonal competition [13,14,38,41,42]. By explicitly incorporating resource limitation and density-dependent feedbacks, our framework extends these models beyond unconstrained growth while retaining a fully stochastic core; moreover, it allows the acquisition of mutations that provide different kind of advantages, in line with recent theories of cancer hallmarks [43–45].

Previous stochastic models that aimed to describe tumor growth using a branching-process approach centered exclusively on driver mutations that confer proliferative advantage, see [13,16,34–37]. In [76], the authors present a recent attempt to incorporate more complex aspects of cancer progression into the model. Their work is aligned with our perspective in adopting the hallmarks framework as a guiding principle, although their analysis is restricted to apoptosis, invasion (metastatic transformation), immortalization, and cell division. Moreover, their model is not fully stochastic, since cell growth is described only through mean trajectories, while stochasticity is confined to what the authors term the “trial” sequence. Furthermore, the model does not account for interactions among clones, which,in our approach, constitute the key mechanism for describing hallmarks related to metabolic reprogramming and to the induction of, and access to, tumor-associated neovasculature.

A major limitation of many tumor evolution models lies in their difficulty in generating heterogeneous cell populations that provide a realistic representation of intratumor heterogeneity (ITH). Two main modelling strategies may be considered to address this issue. The first consists in introducing a spatial structure for the environment in which cells proliferate, so as to promote the segregation of the tumor mass into distinct subpopulations, see for example [39–41]. Under assumptions ensuring unbounded growth, with no saturation of either space or available resources, each spatially segregated niche gives rise to a distinct evolutionary trajectory, thereby leading to increasing genetic diversity. The resulting tumor exhibits a controllable degree of ITH, determined by the extent of segregation among the different subpopulations. A second approach, on which we focus here inspired by [77], consists in considering a well-mixed population of cells with growth limitations and capable of exhibiting different mechanisms of resources exploitation. Both the growth limitation and the different resources exploitations are necessary to show modelled tumors with genetic heterogeneity. Indeed, a growth constraint alone tends to reduce genetic variability, since saturation of the limited resources yields a system in which the relative frequencies of the subclones present at the time the maximum population size is reached can no longer change, thereby fixing the genetic composition at all subsequent times. The model should therefore explicitly incorporate mechanisms of differential exploitation of the limited resources (the type 4-Resource control functional effect), under which subpopulations able to recruit a larger share of the scarce resources can achieve greater growth than others. A similar attempt has been proposed in [42], where a double growth-constraint mechanism acting both on the total population size (global confinement) and on individual subclones (local confinement) is used to mimic competition for shared resources, thereby generating genetically heterogeneous tumors. However, we believe that the ability to evade growth constraints and the ability to differentially dispose of the shared resources have to be mapped into different parts of the model. Moreover we think that those mechanisms should be clone-specific instead of shared by all the population. Indeed, those capabilities can be biologically thought as emerging from the specific set of genetic alterations that have been accumulated by the single clone.

The two approaches to modelling here discussed, the spatially organized population and the well-mixed but subject to competition for limited resources population, lead to genetically heterogeneous tumors though diverging conceptually: we argue that it is not the geometry of space itself that governs tumor evolution, but rather the control and distribution of shared resources. In our framework, competition arises from differential access to these resources and can be modulated by new driver mutations, thus the creation of new spatial compartments is not required to explain clonal diversity. Still, these spatially explicit models remain valuable, and for tumor types where physical architecture plays a dominant role, their principles could be seamlessly integrated into our simulator, further demonstrating its flexibility and generality. It would also be interesting to explore models that are neither fully well-mixed nor fully spatially segregated, but instead display an intermediate degree of local-neighborhood interactions. In this setting, it would be important to determine which results are found to be robust.

Using our model, we performed a large set of systematic simulation experiments to quantify how the evolutionary fate of newly arising subclones depends on both the functional nature of the mutation and the phase of tumor growth at which it is acquired. Specifically, we compared the survival probability and final prevalence of subclones carrying neutral mutations to those acquiring functionally disruptive mutations of different classes (i.e., proliferative advantage, evasion of growth limits, and control or modulation of shared resources) stratifying results by early expanding versus late saturated growth phases of the mass. These experiments consistently revealed a biphasic pattern of tumor evolution. In an initial phase, before the expanding mass reaches the physical and metabolic limits of the surrounding tissue, proliferative advantages can transiently influence clonal dynamics, although they remain confined to low-prevalence ranges. In the subsequent phase, once the available space and nutrients are fully exploited, only mutations that alleviate environmental constraints, such as resistance to resource scarcity or the ability to bypass local limitations, allow clones to escape saturation, continue expanding, and potentially dominate the population.

In conclusion, the proposed framework provides a flexible and integrative approach to exploring tumor evolution by jointly analyzing heterogeneous data sources routinely generated in both clinical practice and research settings. These include longitudinal clinical monitoring data, targeted mutational panels, and whole-exome or whole-genome sequencing data. By integrating these data streams, the framework enables a systematic assessment of how empirical observations intersect with the results of simulation-based evolutionary models. At the same time, the framework allows domain-specific knowledge for a given cancer type to be incorporated into the analysis, supporting hypothesis-driven exploration of tumor evolution. Through an intuitive graphical interface, users can visually inspect evolutionary trajectories and identify the classes of mutational effects that drive distinct tumor progression patterns. This combination of data integration, simulation, and interactive visualization enhances interpretability and provides a practical tool for linking molecular alterations to evolutionary dynamics in cancer.

## Supporting information

S1 Appendix The model. Mathematical details on the cancer progression model.
(PDF)

S2 Appendix The simulation algorithm. Mathematical details on the simulation algorithm.
(PDF)

S3 Appendix Parameter exploration. A detailed analysis of the distributional properties of the simulated tumors for a wide-ranging choice of parameters.
(PDF)
