# Supplementary material for: A new cancer progression model: From synthetic tumors to real data and back
Source: PLoS Comput Biol. 2026 Jun 24;22(6):e1013991. doi: 10.1371/journal.pcbi.1013991 (PMC13327523; doi:10.1371/journal.pcbi.1013991)
Supplement: S1 Appendix — Mathematical details on the cancer progression model. (PDF) [file pcbi.1013991.s001.pdf]

Supporting information

S1 Appendix: the model

S1.1 N is a locally finite rooted tree

The phylogenetic evolution of a tumor is a rooted tree. Rooted trees are collections of elements of the set $\mathcal{U} = \bigcup_{n=0}^{\infty} \mathbb{N}^n$ made of the finite sequences of natural numbers $\mathbb{N}$, with $\mathbb{N}^0 = \emptyset$. On the generic element $u = (u_1, \dots, u_n) \in \mathcal{U}$ we can define $|u| := n$ the generation of u, $p(u) = (u_1, \dots, u_{n-1})$ the parent of u and we will denote as $u|_j := (u_1, \dots, u_j)$ the restriction up to generation j of the element u. A *locally finite ordered rooted tree* (LFORT) τ is a subset of $\mathcal{U}$ satisfying:

1. $\varnothing \in \tau$
2. $u \in \tau \Rightarrow$ the parent $p(u) = (u_1, \dots, u_{n-1}) \in \tau$
3. $\forall u \in \tau\ \exists A_u \in \mathbb{N} \cup 0 : uj = (u_1, \dots, u_n, j) \in \tau \Leftrightarrow j \leq A_u$.

The number A_u is the number of children of u. We will denote with $\mathcal{T}$ the collection of all LFORTs. An example of LFORT is illustrated in Fig A.

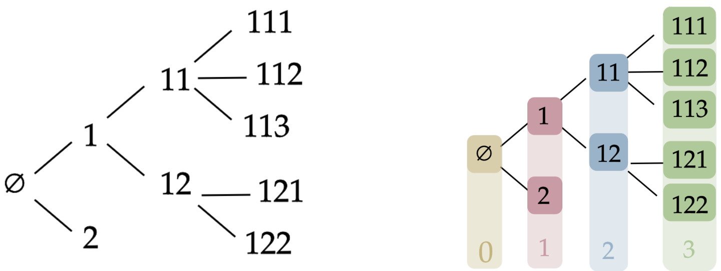

Fig A. Example of Locally Finite Ordered Rooted Tree: on the left the graphical representation of $\tau = \{\varnothing, 1, 2, (1,1), (1,2), (1,1,1), (1,1,2), (1,1,3), (1,2,1), (1,2,2)\}$; on the right, a partial order naturally defined on the *LFORT*, as the generation associated with each population.

Theorem S1.1.1. *$N(t)$ is almost surely a Locally Finite Rooted Tree for any t.*

Proof. Let's prove that, once fixed t, $N(t)$ satisfies all the 3 requirements:

1. $\varnothing \in N(t)$ as $\mathbb{P}(\sigma_\varnothing = 0) = 1$, thus $\mathbb{P}(\sigma_0 \leq 0) = 1$.
2. Given $u \in N(t)$, $\sigma_u \leq t$ by definition and since $\sigma_{p(u)} \leq \sigma_u$, then also $\sigma_{p(u)} \leq t$ which means $p(u) \in N(t)$.
3. Defining $A_u := \#\{v \in N(t) : p(v) = u\}$ as the number of populations with u as parent emerged until time t, $uj \in N(t) \iff j = 0, \dots, A_u$ by definition of A_u. The only proof required is that $A_u < \infty$ with probability 1.
   First of all, let's observe that A_u

   $$(A_u \mid X_{p(u)}(s), s < t) \sim Poisson\left(\mu_{p\!\!\!/(u)} \int_0^t X_u(s)ds\right)$$

   as it is precisely the *Cox process* that we have built Eq. 6 over.

Then,

$$\begin{gathered}\mathbb{P}(A_u < \infty) = 1 \\ \Updownarrow \\ \mathbb{P}(Poisson\,(M) < \infty) = 1\end{gathered}$$

with $M = \mu_{p\hslash(u)} \int_0^t X_u(s)ds$. The Poisson random variable is a.s. finite, provided its mean is finite, thus

$$\begin{gathered}\mathbb{P}(Poisson\,(M) < \infty) = 1 \\ \Updownarrow \\ \mathbb{P}(M < \infty) = 1 \\ \Updownarrow \\ \mathbb{P}\left(\mu_{p\hslash(u)} \int_0^t X_u(s)ds < \infty\right) = 1.\end{gathered}$$

Since $t < \infty$ and trajectories of $X_u(s)$ are *simple functions*, it is enough to prove that $X_u(s)$ doesn't explode in finite time, i.e. that $\mathbb{P}(X_u(s) < \infty) = 1$ for any $s \in [\sigma_u, t]$.

By definition $X_u(s) = Z(s - \sigma_u)$ with Z birth-death process of parameters $a_{p\hslash(u)}$ and $b_{p\hslash(u)}$, and it is well-known (see [1]) that

$$\mathbb{P}(Z(r) = k) = \begin{cases} \alpha & k = 0 \\ (1-\alpha)(1-\beta)^{k-1}\beta & k > 0 \end{cases}$$

$$\text{with} \quad \begin{aligned} \alpha &:= \frac{b - be^{-(a-b)r}}{a - be^{-(a-b)r}}, \\ \beta &:= \frac{a - ae^{-(a-b)r}}{a - be^{-(a-b)r}}, \end{aligned}$$

hence the probability of Z being finite is

$$\begin{aligned} \mathbb{P}(Z(r) < \infty) &= \sum_{k=0}^{\infty} \mathbb{P}(Z(r) = k) \\ &= \alpha + \sum_{k=1}^{\infty} (1-\alpha)(1-\beta)^{k-1}\beta \\ &= \alpha + (1-\alpha)\beta \sum_{k=1}^{\infty} (1-\beta)^{k-1} \\ &= \alpha + (1-\alpha)\beta \sum_{k=0}^{\infty} (1-\beta)^{k} \\ &= \alpha + (1-\alpha)\beta \frac{1}{1-(1-\beta)} \\ &= 1 \end{aligned}$$

□

S1.2 X is a Markov process

The state space S, for the cancer evolution model X, can be equipped with a sum

$$(\tau, \mathbf{x}) + (\tau', \mathbf{x}') := (\tau \cup \tau', (x''_u)_{u \in \tau \cup \tau'}), \quad \text{where } x''_u = \begin{cases} x_u + x'_u & u \in \tau \cap \tau' \\ x_u & u \in \tau \setminus \tau' \\ x'_u & u \in \tau' \setminus \tau \end{cases}$$

with a scalar product on $\mathbb{R}$

$$k \cdot (\tau, \mathbf{x}) := (\tau, (kx_u)_{u \in \tau})$$

and with a norm:

$$\|(\tau, \mathbf{x})\| := \sqrt{\sum_{u \in \tau} x_u^2}.$$

Hence $(S, +, \cdot)$ is a linear space and $(S, \|\cdot\|)$ is a metric space. For functions defined over a normed vector space there exists a natural definition of differentiability (Fréchet derivative).

Theorem S1.2.1. *$X(t)$ is a Markov process*

Proof. Given a time t and conditioning the process to be in $(\tau, \mathbf{x})$ at time t, we can consider the possible states $(\tau', \mathbf{x}')$ in which the process can be found after an infinitesimal amount of time dt:

1. $(\tau, \mathbf{x} \pm \mathbf{e}_u)$ for some $u \in \tau$ (where $\mathbf{e}_u$ is 0 unless for 1 in position u), consequent to a birth or death occurring in population u alive at time t;

2. $(\tau \cup \{u\}, (\mathbf{x}, 1))$ for some $u \notin \tau$ such that $p(u) \in \tau$, corresponding to the rising of population u from a population $p(u)$ alive at time t.

As it concerns 1, the waiting time for this event is that of a birth-death processes conditioned to be in state x_u, which is an exponential random variable of parameter $(a_{ph(u)} + b_{ph(u)})x_u$. Therefore, the waiting time for an event of kind 1, is the minimum between n independent exponentially distributed random variables, which is an exponential random variable itself with parameter the sum of all the parameters:

$$Exp\left(\sum_{u \in \tau} (a_{ph(u)} + b_{ph(u)})x_u\right). \tag{S1.1}$$

Regarding 2, the distribution of the waiting time is explicitly defined in Eq. 6. Since we are supposing that $u \notin \tau$ at time t, we need to condition the distribution to $\sigma_u > t$:

$$
\begin{aligned}
&\mathbb{P}\left(\sigma_u \le t+dt \mid \sigma_u > t, X_{p(u)}(s), s\in[t,t+dt]\right)\\
&\qquad= \frac{\mathbb{P}\left(\sigma_u \le t+dt, \sigma_u > t \mid X_{p(u)}(s), s\in[t,t+dt]\right)}{\mathbb{P}\left(\sigma_u > t \mid X_{p(u)}(s), s\in[t,t+dt]\right)}\\
&\qquad= \frac{\mathbb{P}\left(\sigma_u \le t+dt \mid X_{p(u)}(s), s\in[t,t+dt]\right) - \mathbb{P}\left(\sigma_u \le t \mid X_{p(u)}(s), s\in[t,t+dt]\right)}{\mathbb{P}\left(\sigma_u > t \mid X_{p(u)}(s), s\in[t,t+dt]\right)}\\
&\qquad= \frac{1 - e^{-\mu_{p\hbar(p(u))}\int_0^{t+dt} X_{p(u)}(s)ds} - 1 + e^{-\mu_{p\hbar(p(u))}\int_0^{t} X_{p(u)}(s)ds}}{e^{-\mu_{p\hbar(p(u))}\int_0^{t} X_{p(u)}(s)ds}}\\
&\qquad= \frac{- e^{-\mu_{p\hbar(p(u))}\int_0^{t+dt} X_{p(u)}(s)ds} + e^{-\mu_{p\hbar(p(u))}\int_0^{t} X_{p(u)}(s)ds}}{e^{-\mu_{p\hbar(p(u))}\int_0^{t} X_{p(u)}(s)ds}}\\
&\qquad= \frac{- e^{-\mu_{p\hbar(p(u))}\int_0^{t+dt} X_{p(u)}(s)ds}}{e^{-\mu_{p\hbar(p(u))}\int_0^{t} X_{p(u)}(s)ds}} + 1\\
&\qquad= -e^{-\mu_{p\hbar(p(u))}\int_0^{t+dt} X_{p(u)}(s)ds + \mu_{p\hbar(p(u))}\int_0^{t} X_{p(u)}(s)ds} + 1\\
&\qquad= 1 - e^{-\mu_{p\hbar(p(u))}\int_t^{t+dt} X_{p(u)}(s)ds}.
\end{aligned}
$$

Moreover, $X_{p(u)}(s) = x_{p(u)}$ for any $s \in [t, t+dt]$, as otherwise 1 instead of 2 would be the very next event, so

$$\mathbb{P}\left(\sigma_u \le t+dt \mid \sigma_u > t, X_{p(u)}(s), s\in[t,t+dt]\right) = 1 - e^{-\mu_{p\hbar(p(u))}x_{p(u)}dt}$$

which is the probability of an exponential random variable of parameter $\mu_{p\hbar(p(u))}x_{p(u)}$ being smaller than dt. Again the waiting time for *any* of the possible events of type 2 is the minimum between the holding times associated with each possible $p(u) \in \tau$, hence it is

$$Exp\left(\sum_{u\in\tau}\mu_{p\hbar(u)}x_u\right). \tag{S1.2}$$

Finally, the minimum between the quantities in Eq. (S1.1) and Eq. (S1.2) is an exponential again with the sum of the two parameters as parameter, which gives the distribution of the holding time in state $(\tau, \mathbf{x})$ for the process:

$$T_{(\tau,\mathbf{x})} \sim Exp\left(\sum_{u\in\tau}(a_{p\hbar(u)} + b_{p\hbar(u)} + \mu_{p\hbar(u)})x_u\right).$$

The memoryless property of an exponential random variable guarantees the independence from the past of the system once conditioned on a certain state at a fixed time. Furthermore, exploiting properties of the birth-death processes and the definition given in Eq. (6), it is possible to derive the transition probabilities for the events enlisted before:

1. $(\tau, \mathbf{x}) \to (\tau, \mathbf{x} + \mathbf{e}_u)$ for $u \in \tau$ (birth in population u)

$$
\begin{aligned}
&\mathbb{P}(X(t+dt) = (\tau, \mathbf{x}+\mathbf{e}_u) \mid X(t) = (\tau,\mathbf{x}), T_{(\tau,\mathbf{x})} \in dt)\\
&\qquad= \frac{a_{p\hbar(u)}x_u}{\sum_{v\in\tau}(a_{p\hbar(v)} + b_{p\hbar(v)} + \mu_{p\hbar(v)})x_v}
\end{aligned}
$$

2. $(\tau, \mathbf{x}) \to (\tau, \mathbf{x} - \mathbf{e}_u)$ for $u \in \tau$ (death in population u)

$$
\begin{aligned}
&\mathbb{P}(X(t+dt) = (\tau, \mathbf{x}-\mathbf{e}_u) \mid X(t) = (\tau,\mathbf{x}), T_{(\tau,\mathbf{x})} \in dt)\\
&\qquad= \frac{b_{p\hbar(u)}x_u}{\sum_{v\in\tau}(a_{p\hbar(v)} + b_{p\hbar(v)} + \mu_{p\hbar(v)})x_v}
\end{aligned}
$$

3. $(\tau, \mathbf{x}) \to (\tau \cup \{u\}, (\mathbf{x}, 1))$ for some $u \notin \tau$ such that $p(u) \in \tau$ (appearance of population u)

$$\mathbb{P}(X(t+dt) = (\tau \cup \{u\}, (\mathbf{x}, 1)) \mid X(t) = (\tau, \mathbf{x}), T_{(\tau,\mathbf{x})} \in dt) = \frac{\mu_{p\hbar(p(u))} x_u}{\sum_{v \in \tau} (a_{p\hbar(v)} + b_{p\hbar(v)} + \mu_{p\hbar(v)}) x_v}$$

□

S1.3 Size-dependencies

To account for the limiting effect of finite resources on cell growth, it is required to transition from a classical birth-and-death model that on average follows an exponential expansion, to a logistic growth model, see Fig B. The idea behind this adjustment is that the growth rate of the population is directly proportional to its size, while also decreasing quadratically as the population size increases:

$$\dot{x}(t) = \lambda x(t)$$

differential equation governing the basic exponential growth

$$\dot{x}(t) = \lambda x(t) \left(1 - \frac{x(t)}{K}\right)$$

differential equation governing the logistic growth

Extending this idea to a multi-population setting, it is natural to assume that each

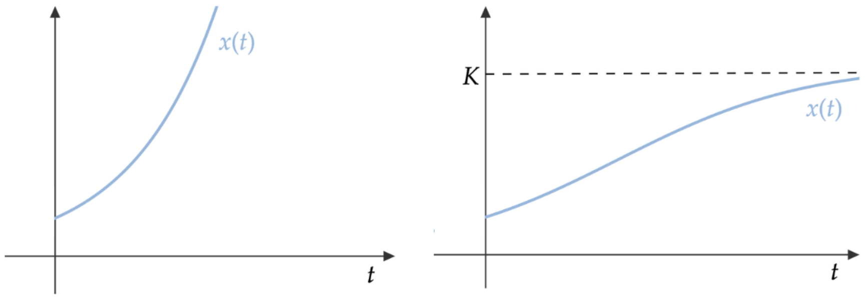

Fig B. Exponential growth on the left: the number of cells enlarges indefinitely. Logistic growth on the right: the number of cells reaches a maximum that is called *carrying capacity* (K).

population is constrained by the overall size of the tumor, represented by $\sum_i x_i(t)$. In a deterministic setting, this leads to the dynamical system

$$\dot{x_i}(t) = \lambda_i x_i(t) \left(1 - \frac{\sum_j x_j(t)}{K}\right),$$

where λ_i represents the growth rate of population i.

This adjustment follows a "first come, first served" principle, where early populations grow without limitation until the tumor reaches a certain size. At that point, however, new populations encounter such strong growth restrictions that they may be barely detectable. This limitation motivated us to adopt a slightly more complex framework: a Lotka-Volterra interaction model, aligning with established literature on the subject.

The **Lotka-Volterra model** can be viewed as an extension of the logistic model, where each population is influenced by others through a parameter α_{ij}, representing the effect that population j has on population i. This leads to a system of equations of the form:

$$\dot{x_i}(t) = \lambda_i x_i(t) \left(1 - \frac{\sum_j \alpha_{ij} x_j(t)}{k_i}\right).$$

This version of the Lotka-Volterra equation is particularly intuitive, as it maintains the concept of resource limitation through a carrying capacity (one for each population) while incorporating additional parameters for flexibility. However, in this representation, some parameters are superfluous, so we set $\alpha_{ii} = 1$ to remove unnecessary constants without reducing the system's degrees of freedom.

$$\dot{x_i}(t) = \lambda_i x_i(t) \left(1 - \frac{x_i(t) + \sum_{j \neq i} \alpha_{ij} x_j(t)}{k_i}\right). \tag{S1.3}$$

In this setting, term $1 - \frac{\sum \cdots}{\cdots}$ represent a limiting term for the growth due to the carrying capacity: as the numerator of the fraction get closer to the carrying capacity of the considered population, the term get smaller and so does the speed of growth of the population. In this sense, α_{ij} is the effect that population j has on population i:

- if $\alpha_{ij} = 0$ the size of population j has no effect on the reduction of the growth of x;
- if $\alpha_{ij} > 0$ population j size limits population i growth as the $\alpha_{ij} x_j$ term of the sum is positive, in particular:
  - if $\alpha_{ij} = 1$ each element of population j counts as an element of population i;
  - if $\alpha_{ij} > 1$ each element of population j counts as more than one element of population i, meaning that it has a major weight in the global limitation, hence i will be strongly negatively effected by j;
  - if $\alpha_{ij} < 1$ each element of population j counts as less than one element of population i, meaning that it has a minor weight in the global limitation, hence i will be mildly negatively effected by j;
- if $\alpha_{ij} < 0$ the term $\alpha_{ij} x_j$ of the sum is negative, hence the effect that j has on i is a positive one, allowing to enlarge the possibility it has to expand.

In our model, we will mimic this size-dependence letting the growth parameter of the birth-death process to be multiplied by the limiting term $1 - \frac{\sum \cdots}{\cdots}$.

To better clarify the reciprocal interactions and appreciate the explainability of the model built this way, it is possible to study the result of the interaction of a general couple of populations (let them be called x and y from now on) in the simpler deterministic setting.

The dynamical system we are considering is therefore

$$\begin{cases} \dot{x}(t) = \lambda_1 x(t) \left(1 - \frac{x(t) + \alpha_{12} y(t)}{k_1}\right) \\ \dot{y}(t) = \lambda_2 y(t) \left(1 - \frac{y(t) + \alpha_{21} x(t)}{k_2}\right) \end{cases}, \tag{S1.4}$$

with $k_1, k_2 > 0$ and $\lambda_1, \lambda_2 \geq 0$, as we are only interested in the first quadrant of the (x, y) plane and in populations that are not subject to pure death.

Equilibrium points Let us restrict to the case $\lambda_1, \lambda_2 > 0$ for now, we will later explore the particular case in which one or both the grow rates are zero. Following [2], by analysing the equilibrium points and their stability and applying the general results to our specific case, one can derive that the system exhibits five possible regimes, each corresponding to a distinct relative configuration of the parameters, as shown in Fig C. For our purposes we can assume that a population, namely x, is already present, while the other appears later. Population x doesn't necessarily represent the original tumor clone neither an ancestor of y; it could be any population independent of y. This assumption is simply a convention to designate which population appears first. With this interpretation we can consider for our study orbits starting in $(x_0, 1)$ for $x_0 > 0$, as

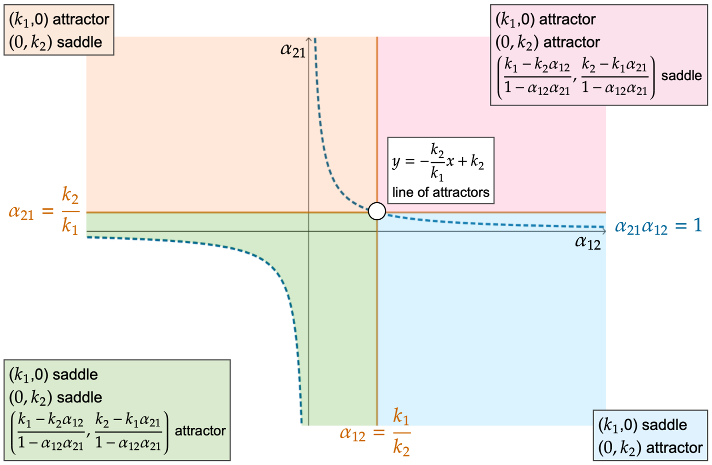

Fig C. Stability of the equilibria of system in Eq. (S1.4) for different values of the parameters.

population y will always originate from a single newly mutated cell and x_0 will be the size x has reached when y appears.

In such a framework the five regimes correspond to four possible ecological occurrences:

Predominance of population x: Whenever α_{12} is smaller than $\frac{k_1}{k_2}$ and α_{21} is greater then $\frac{k_2}{k_1}$ (orange area of Fig C), the system will be absorbed in $(k_1, 0)$ no matter the starting point. This behavior corresponds to the native population being stronger and fitter than the newcomer: as visible in Fig D, clone y can enlarge for a bit, but eventually it will die out as the most resources are stolen by x.

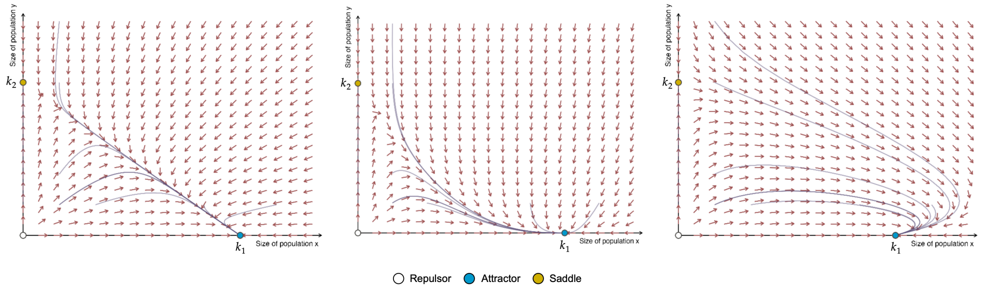

Fig D. Examples of phase portraits of the system in Eq. (S1.4) with parameters in the orange area plotted in Fig C.

Predominance of population y: Whenever α_{12} is greater than $\frac{k_1}{k_2}$ and α_{21} is smaller then $\frac{k_2}{k_1}$ (light blue area of Fig C), the system will be absorbed in $(0, k_2)$ no matter the starting point. This behavior corresponds to the newly appeared population being stronger and fitter than the native: as visible in Fig E, clone x can resist for a while, but soon or later it will die out as the most resources are stolen by y.

Predominance non-predefined When both α_{12} is greater than $\frac{k_1}{k_2}$ and α_{21} is greater then $\frac{k_2}{k_1}$ (pink area of Fig C), the system will either be absorbed in $(k_1, 0)$

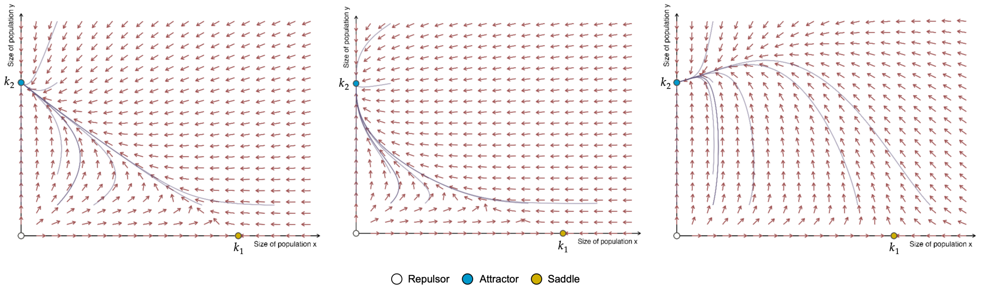

Fig E. Examples of phase portraits of the system in Eq. (S1.4) with parameters in the light blue area plotted in Fig C.

or in $(0, k_2)$, based on the starting point, i.e. on the size of x when first cell of y appears. This behavior reflects that the newly appeared population is strong enough to overtake x only up to a certain threshold size: as shown in Fig F, the parameters set a limit for the size of x that y can dominate and the saddle plays the role of the physical threshold for the orbits. Once this threshold is defined, that is to say once $\alpha_{12}, \alpha_{21}, k_1$ and k_2 are known, the final outcome will be determined by the initial proportion between the clones.

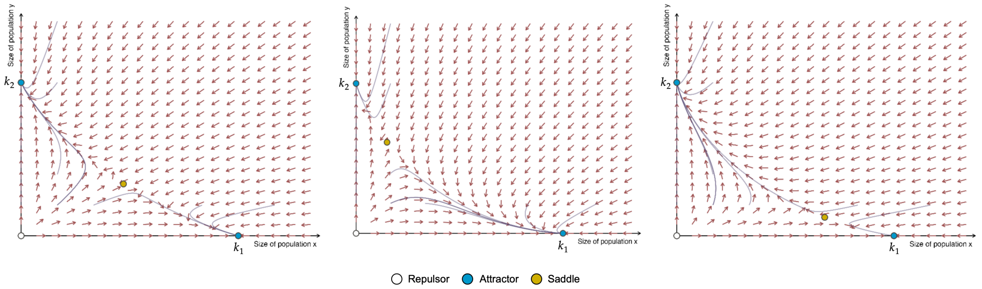

Fig F. Examples of phase portraits of the system in Eq. (S1.4) with parameters in the pink area plotted in Fig C.

Equilibrium predictable When both α_{12} is smaller than $\frac{k_1}{k_2}$ and α_{21} is smaller then $\frac{k_2}{k_1}$ but at the same time $\alpha_{12}\alpha_{21} < 1$ (green area of Fig C), the two populations will reach an intermediate equilibrium, namely $\left(\frac{k_1 - k_2\alpha_{12}}{1-\alpha_{12}\alpha_{21}}, \frac{k_2 - k_1\alpha_{21}}{1-\alpha_{12}\alpha_{21}}\right)$. This behavior reflects a more complicated interaction compared to those presented before as there is no a priori stronger clone.

In particular, it is possible to consider the position of the compromised equilibrium compared to the equilibrium the population would have reached without the other population appearing (k_1 for x and k_2 for y):

- The equilibrium is advantageous for x if $\frac{k_1 - k_2\alpha_{12}}{1-\alpha_{12}\alpha_{21}} \geq k_1$, which is equivalent (as we are limiting to the case $\alpha_{12}\alpha_{21} < 1$, $\alpha_{12} \leq \frac{k_1}{k_2}$, $\alpha_{21} \leq \frac{k_2}{k_1}$) to $\alpha_{12} \leq 0$. In particular for $\alpha_{12} = 0$, the equilibrium have ascissa k_1, hence x is neither advantaged nor disadvantaged by the compromise.
- The equilibrium is advantageous for y if $\frac{k_2 - k_1\alpha_{21}}{1-\alpha_{12}\alpha_{21}} \geq k_1$, which is equivalent (as we are limiting to the case $\alpha_{12}\alpha_{21} < 1$, $\alpha_{12} \leq \frac{k_1}{k_2}$, $\alpha_{21} \leq \frac{k_2}{k_1}$) to $\alpha_{21} \leq 0$. In particular for $\alpha_{21} = 0$, the equilibrium have ascissa k_2, hence y is neither advantaged nor disadvantaged by the compromise.

There is a further interesting point of view when populations are two clones of a same tumor, that is the global advantage/disadvantage given to the tumor by the presence of the two clones compared to the size it would have reach with one of the two populations only. This is equivalent to wondering

$$\frac{k_1 - k_2\alpha_{12}}{1-\alpha_{12}\alpha_{21}} + \frac{k_2 - k_1\alpha_{21}}{1-\alpha_{12}\alpha_{21}} \geq \max\{k_1, k_2\}$$

which branches into

$$\begin{array}{c} \text{if } k_1 \geq k_2 \\ k_1 - k_2\alpha_{12} + k_2 - k_1\alpha_{21} \geq k_1(1-\alpha_{12}\alpha_{21}) \\ k_1(1-\alpha_{21}) + k_2(1-\alpha_{12}) - k_1(1-\alpha_{12}\alpha_{21}) \geq 0 \\ k_1(\alpha_{21}\alpha_{12} - \alpha_{21}) + k_2(1-\alpha_{12}) \geq 0 \\ -k_1\alpha_{21}(1-\alpha_{12}) + k_2(1-\alpha_{12}) \geq 0 \\ (1-\alpha_{12})(k_2 - k_1\alpha_{21}) \geq 0 \\ (\text{since } \alpha_{21} \leq \frac{k_2}{k_1} \text{ and } \alpha_{12} \leq \frac{k_1}{k_2}) \\ \alpha_{12} \leq \min\{1, \frac{k_1}{k_2}\} \end{array} \qquad \begin{array}{c} \text{if } k_2 \geq k_1 \\ k_1 - k_2\alpha_{12} + k_2 - k_1\alpha_{21} \geq k_2(1-\alpha_{12}\alpha_{21}) \\ k_1(1-\alpha_{21}) + k_2(1-\alpha_{12}) - k_2(1-\alpha_{12}\alpha_{21}) \geq 0 \\ k_1(1-\alpha_{21}) + k_2(\alpha_{21}\alpha_{12} - \alpha_{12}) \geq 0 \\ k_1(1-\alpha_{21}) - \alpha_{21}k_2(1-\alpha_{12}) \geq 0 \\ (k_1\alpha_{21}k_2)(1-\alpha_{12}) \geq 0 \\ (\text{since } \alpha_{21} \leq \frac{k_2}{k_1} \text{ and } \alpha_{12} \leq \frac{k_1}{k_2}) \\ \alpha_{21} \leq \min\{1, \frac{k_2}{k_1}\} \end{array}$$

The green area plotted in Fig C can be thus further subdivided as visible in Fig G Some phase portrait examples are visible in Fig H

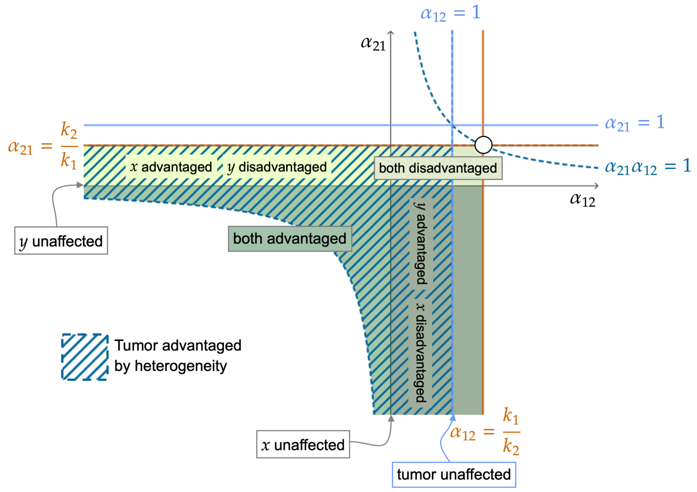

Fig G. Different effects the middle-ground equilibrium $\left(\frac{k_1-k_2\alpha_{12}}{1-\alpha_{12}\alpha_{21}}, \frac{k_2-k_1\alpha_{21}}{1-\alpha_{12}\alpha_{21}}\right)$ has on x and y based on the values of the parameters. Each population can be advantaged or disadvantaged by the compromise, based on the ending size it can reach when in equilibrium: this creates 4 different regions identified with different colors. In addiction the striped layer shows how the global tumor size is affected from the relationships among the two clones, subdividing competition and parasitism into a zone of sacrifice for the major good and a zone of toxic competition (either sacrifice of one or of both). This picture refers to the case $k_1 > k_2$: the vice versa is an equivalent figure with $\alpha_{21} = 1$ filling the role of $\alpha_{12} = 1$.

Equilibrium non-predefined When $\alpha_{12} = \frac{k_1}{k_2}$ and $\alpha_{21} = \frac{k_2}{k_1}$, system in Eq. (S1.4) becomes

$$\begin{cases} \dot{x}(t) = \lambda_1 x(t)\left(1 - \frac{x(t)}{k_1} - \frac{y(t)}{k_2}\right) \\ \dot{x}(t) = \lambda_2 y(t)\left(1 - \frac{x(t)}{k_1} - \frac{y(t)}{k_2}\right) \end{cases}$$

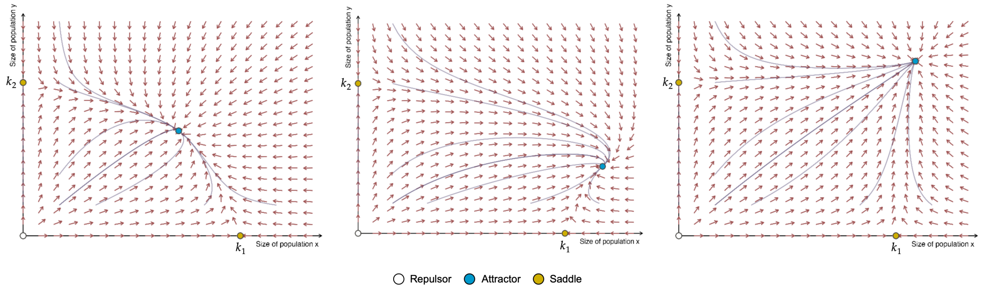

Fig H. Examples of phase portraits of the system in Eq. (S1.4) with parameters in the green area (Fig C). The first one refers to the case in which the equilibrium is disadvantageous for both the populations, the second one shows an advantage of x at the expenses of y, in the third one the middle-ground equilibrium benefits both the populations.

which makes it possible to derive an explicit formula for the orbits starting in $(x_0, 1)$, by solving

$$\frac{dy}{dx} = \frac{\lambda_2}{\lambda_1}\frac{y}{x}$$
$$\frac{1}{y}dy = \frac{\lambda_2}{\lambda_1}\frac{1}{x}dx$$
$$\log(y) = \frac{\lambda_2}{\lambda_1}\left(\log(x) - \log(x_0)\right)$$
$$y = \left(\frac{x}{x_0}\right)^{\frac{\lambda_2}{\lambda_1}}$$

hence the equilibrium in which the orbit will be absorbed is the solution of

$$\begin{cases} \left(\frac{x}{x_0}\right)^{\frac{\lambda_2}{\lambda_1}} = -\frac{k_2}{k_1}x + k_2 \\ y = -\frac{k_2}{k_1}x + k_2 \end{cases}$$

that depends not only on the parameters λ (that don't have a role in the previous cases), but also on the number of cells of population x when y appears. Some examples of orbits are visible in Fig I.
It is possible to observe that the tendency of such system is to favor the newly appeared population up to a certain threshold regarding the size of the native population, and to favor the native population once overcome such threshold, i.e. when it was already too large to reverse the proportions between the 2. In particular the ration $\frac{\lambda_2}{\lambda_1}$ operates on this threshold lowering it for $\lambda_1 > \lambda_2$ and raising it for $\lambda_2 > \lambda_1$. The special case $\lambda_1 = \lambda_2$ corresponds to proportions between the populations being maintained.

Whatever the attractor, however, both the populations will be negatively affected by the middle-ground equilibrium as for positive x and y, all the points of the line $y = -\frac{k_2}{k_1}x + k_2$ have smaller abscissa than k_1 and smaller ordinate than k_2. The global size of the tumor is reduced as well compared to the size it could reach if only the population capable of greater expansion was there. As a matter of facts, considering a general point on the line of attractors $\left(x, -\frac{k_2}{k_1}x - k_2\right)$, the total size $x + y$ is:

$$-\frac{k_2}{k_1}x + k_2 + x \geq \max\{k_1, k_2\}$$

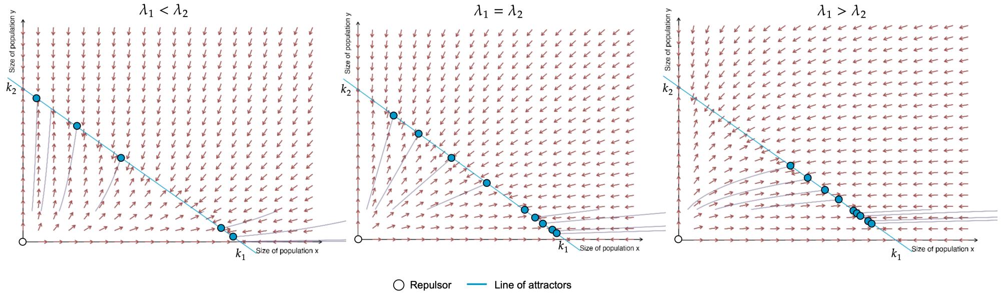

Fig I. Examples of phase portraits of the system in Eq. (S1.4) with parameters $\alpha_{12} = \frac{k_1}{k_2}$ and $\alpha_{21} = \frac{k_2}{k_1}$.

$$
\begin{array}{cc}
\text{(in case } k_1 \geq k_2) & \text{(in case } k_2 \geq k_1) \\
-\frac{k_2}{k_1}x + k_2 + x \geq k_2 & -\frac{k_2}{k_1}x + k_2 + x \geq k_1 \\
\left(1 - \frac{k_2}{k_1}\right) x \geq 0 & \frac{k_1 - k_2}{k_1} x \geq k_1 - k_2 \\
\left(\text{since } \dfrac{k_2}{k_1} \geq 1\right) & (\text{since } k_1 - k_2 \geq 0) \\
x \leq 0 & x \geq k_1
\end{array}
$$

which are points outside of the I quadrant.

The trivial case $\lambda_1 = 0$ or $\lambda_2 = 0$ can now be faced. The system in Eq. (S1.4) loses the meaning in one of the equations when one of the growth rates is null, resulting in a constant in size population and a logistic growth of the other:

$$
\begin{cases} \dot{x}(t) = 0 \\ \dot{y}(t) = \lambda_2 y(t) \left(1 - \frac{y(t) + \alpha_{21} x(t)}{k_2}\right) \end{cases}
\qquad
\begin{cases} \dot{x}(t) = \lambda_1 x(t) \left(1 - \frac{x(t) + \alpha_{12} y(t)}{k_1}\right) \\ \dot{y}(t) = 0 \end{cases}
$$

$$
\begin{cases} x(t) = x_0 \\ \dot{y}(t) = \lambda_2 y(t) \left(1 - \frac{y(t) + \alpha_{21} x_0}{k_2}\right) \end{cases}
\qquad
\begin{cases} \dot{x}(t) = \lambda_1 x(t) \left(1 - \frac{x(t) + \alpha_{12} y_0}{k_1}\right) \\ y(t) = y_0 \end{cases}
$$

$$
\begin{cases} x(t) = x_0 \\ y(t) = \frac{e^{\left(\lambda_2 - \frac{\alpha_{21}}{k_2} x_0\right)t}}{1 + e^{\left(\lambda_2 - \frac{\alpha_{21} x_0}{k_2} x_0\right)t}} (k_2 + \alpha_{21} x_0). \end{cases}
\qquad
\begin{cases} x(t) = \frac{e^{\left(\lambda_1 - \frac{\alpha_{12}}{k_1} y_0\right)t}}{1 + e^{\left(\lambda_1 - \frac{\alpha_{12}}{k_1} y_0\right)t}} (k_1 + \alpha_{12} y_0) \\ y(t) = y_0. \end{cases}
$$

Thus, The system will be absorbed respectively in:

$$
(x_0, k_2 - \alpha_{21} x_0) \qquad (k_1 - \alpha_{12} y_0, y_0).
$$

Lastly, if both λ_1 and λ_2 are null, the system is stuck in (x_0, y_0).
Ecological relationships Merging the information deriving from the equilibrium states study and the observations made in the very beginning about the sign of α_{12} and α_{21}, we can derive an ecological interpretation of the interaction between two populations depicted by the system in Eq. (S1.4) and starting in $(x_0, 1)$, visible in Fig J.

It is possible to appreciate that the Lotka-Volterra model analyzed, effectively captures the complex biological interactions that may occur between two populations, that is to say:

Competition where two species compete for the same limited resources, negatively affecting both;

Parasitism where one species (the parasite) benefits at the expense of the other (the host);

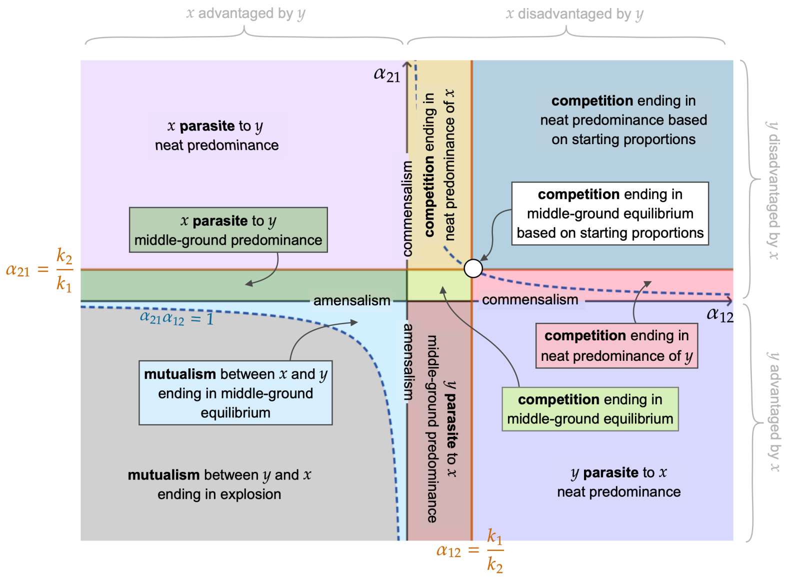

Fig J. Ecological relationships between two populations described in Eq. (S1.4) based on the values assumed by the parameters α and k. Each population growth can be positively or negatively affected by the presence of the other population. The growth rates λ do not have a role in determining how two populations interact and their effect on the stable point is visible only in the spacial case $\alpha_{12} = \frac{k_1}{k_2}$, $\alpha_{21} = \frac{k_2}{k_1}$ (the white point); in all the other cases, growth rates only play a role in the transitory phase and do not affect the stationary limit. The zone identified are characterized by *neat predominance* of one of the populations (eventually based on starting points or growth rates), *middle-ground equilibrium* (advantageous for none, one or both the populations) or *explosion* to $+\infty$ (something we will no further discuss being an unrealistic possibility).

Commensalism where one species benefits while the other is neither helped nor harmed;

Amensalism where one species is harmed while the other remains unaffected;

Mutualism : where both species benefit from the interaction;

References

1. Bailey NT. The elements of stochastic processes with applications to the natural sciences. John Wiley & Sons; 1991.

2. Murray JD. In: Models for Interacting Populations. Springer New York; 1993. p. 79–118. Available from: http://dx.doi.org/10.1007/978-0-387-22437-4_3.
