# Supplementary material for: A new cancer progression model: From synthetic tumors to real data and back
Source: PLoS Comput Biol. 2026 Jun 24;22(6):e1013991. doi: 10.1371/journal.pcbi.1013991 (PMC13327523; doi:10.1371/journal.pcbi.1013991)
Supplement: S2 Appendix — Mathematical details on the simulation algorithm. (PDF) [file pcbi.1013991.s002.pdf]

Supporting information

S2 Appendix: the simulation algorithm

Algorithm 1: The simulation algorithm

input: $N_0 = (u_1, u_2, \dots)$, ▷ Starting genotypes
$\Phi_0 = (ph(u_1), ph(u_2), \dots)$, ▷ Starting phenotypes
$X_0 = (x_{u_1}, x_{u_2}, \dots)$, ▷ Initial number of cells of each genotype
$F = ((F_1, \theta_1), \dots, (F_I, \theta_I))$, ▷ Functional event list
$r = (r_1, \dots, r_I)$, ▷ Frequency distribution of functional events
ϵ_{rel}, ▷ Relative error tolerance
printing_checkpoints = (0, . . . , ending_time), ▷ Output time points

$t = 0$
$N = N_0$
$\Phi = \Phi_0$
$X = X_0$
checkpoint_counter = 0
save t, N, Φ, X
while $t <$ *ending_time* **do**
  a = local_birth_rates(X, Φ, θ) ▷ Adjust birth rates, see eq.(10)
  b = local_death_rates(X, Φ, θ) ▷ Adjust death rates, see eq.(10)
  μ = local_mutation_rates(X, Φ, θ) ▷ Adjust mutation rates, see eq.(10)
  Δ = max_timestep($X, \Phi, a, b, \mu, \theta, \epsilon_{rel}$) ▷ Set maximum timestep see eq.(S2.9)
  for $u \in N$ **do**
    p_u = birthdeath_prob_dens(a_u, b_u, Δ) ▷ Daughter distribution, see eq.(15)
    Sample $z \sim$ Multinomial(X_u, p_u)
    Set $X_u^{new} = \sum_i i \cdot z_i$
    n_newmut $\sim$ Poisson $\left(\mu_u \frac{\Delta}{2}(X_u + X_u^{new})\right)$ ▷ Acquired new mutations
    for $i \in 1, 2, \dots,$ *n_newmut* **do**
      $v = (u, A_u + 1)$ ▷ where A_u are u's number of daughters
      Sample $f_{new} \sim r$
      if $f_{new} \in ph(u)$ **then**
        $ph(v) = ph(u)$
      else
        $ph(v) = ph(u) \cup f_{new}$
      $X_v = 1$
      $X^{new} = (X^{new}, X_v)$
      $N = (N, v)$
      $\Phi = (\Phi, ph(v))$
  $t = t + \Delta$
  $X = X^{new}$
  if *time* $>$ *printing_checkpoints(checkpoint_counter)* **then**
    save t, N, Φ, X
    checkpoint_counter = checkpoint_counter +1

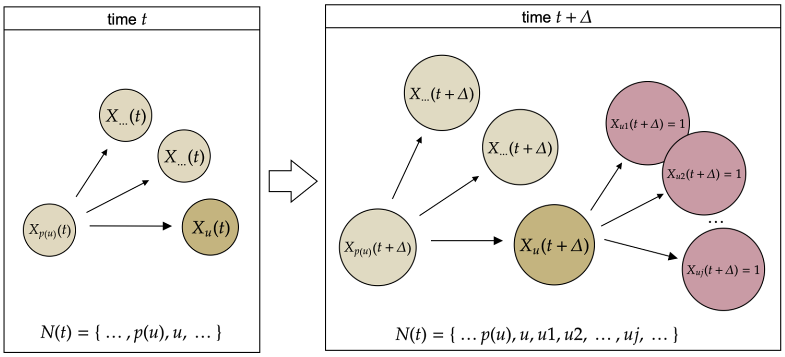

Fig A. Graphical representation of a simulating step. Focusing on population u, the simulation will jump from time t to time $t + \Delta$, computing $X_u(t + \Delta)$; then it will sample the number j of daughter populations of u and set $X_{u1}(t + \Delta) = \cdots = X_{uj}(t + \Delta) = 1$.

S2.1 Bounds on the time step Δ

In this Section, we report the mathematical results used to determine the time step Δ such that the maximum simulation error is controlled. Given the process $X(t)$ is in state $(\tau, \mathbf{x})$, we want to evaluate the expected state in which we will find the process after a time step Δ and chose Δ sufficiently small that $a.((\cdot))$ and $\mu.((\cdot))$ did not change too much. Hence, for any $u \in \tau$ and for any ϵ, we are looking for Δ such that:

$$\left| a_{p\hbar(u)}(m_{(\tau,\mathbf{x})}(\Delta)) - a_{p\hbar(u)}(\tau, \mathbf{x}) \right| < \epsilon, \tag{S2.1}$$

$$\left| \mu_{p\hbar(u)}(m_{(\tau,\mathbf{x})}(\Delta)) - \mu_{p\hbar(u)}(\tau, \mathbf{x}) \right| < \epsilon, \tag{S2.2}$$

with $m_{(\tau,\mathbf{x})}(\Delta) = \mathbb{E}(X(\Delta) \mid X(0) = (\tau, \mathbf{x}))$.

The state space S of the process X is countable, thus the generator is of the form $\mathcal{A} f(s) = \sum_{s' \in S} q_{ss'} f(s')$ for some $q_{ss'} = p'_{ss'}(0)$ where $p_{ss'}(t) = \mathbb{P}(X(t) = s' \mid X(0) = s)$, see [1]. Indeed, the generator given in eq. (8) satisfies this equality, once set $s = (\tau, \mathbf{x})$ and

$$q_{ss'} = \begin{cases} a_{p\hbar(u)}(\tau, \mathbf{x}) x_u & s' = (\tau, \mathbf{x} + \mathbf{e}_u), u \in \tau \\ x_u & s' = (\tau, \mathbf{x} - \mathbf{e}_u), u \in \tau \\ \mu_{p\hbar(u)}(\tau, \mathbf{x}) x_u & s' = (\tau \cup \{u(A_u(\tau) + 1)\}, (\mathbf{x}, 1)), u \in \tau \\ -(a_{p\hbar(u)}(\tau, \mathbf{x}) + 1 + \mu_{p\hbar(u)}(\tau, \mathbf{x})) x_u & s' = s \\ 0 & \text{otherwise.} \end{cases}$$

Therefore, the expected state after a timestep Δ is given as

$$\begin{aligned}
m_{(\tau,\mathbf{x})}(\Delta) &= \sum_{s'\in S} s' p_{ss'}(\Delta) \\
&= \sum_{s'\in S} s'(p_{ss'}(0) + p'_{ss'}(0)\Delta + o(\Delta)) \\
&= \sum_{s'\in S} s'(\delta_s(s') + q_{ss'}\Delta + o(\Delta)) \\
&= s + \sum_{s'\in S} s' q_{ss'}\Delta + o(\Delta) \\
&= (\tau,\mathbf{x}) + \sum_{u\in\tau}\Big[a_{p\hbar(u)}(\tau,\mathbf{x})x_u(\tau,\mathbf{x}+\mathbf{e}_u) + x_u(\tau,\mathbf{x}-\mathbf{e}_u) + \\
&\qquad + \mu_{p\hbar(u)}(\tau,\mathbf{x})x_u(\tau\cup\{u(A_u(\tau)+1)\},(\mathbf{x},1)) + \\
&\qquad - (a_{p\hbar(u)}(\tau,\mathbf{x}) + 1 + \mu_{p\hbar(u)}(\tau,\mathbf{x}))x_u(\tau,\mathbf{x})\Big]\Delta + o(\Delta) \\
&= (\tau,\mathbf{x}) + \sum_{u\in\tau}\Big[a_{p\hbar(u)}(\tau,\mathbf{x})\left((\tau,\mathbf{x}+\mathbf{e}_u) - (\tau,\mathbf{x})\right) + \left((\tau,\mathbf{x}-\mathbf{e}_u) - (\tau,\mathbf{x})\right) + \\
&\qquad + \mu_{p\hbar(u)}(\tau,\mathbf{x})\left((\tau\cup\{u(A_u(\tau)+1)\},(\mathbf{x},1)) - (\tau,\mathbf{x})\right)\Big]x_u\Delta + o(\Delta) \\
&= (\tau,\mathbf{x}) + \sum_{u\in\tau}\Big[a_{p\hbar(u)}(\tau,\mathbf{x})(\{u\},1) - (\{u\},1) + \\
&\quad \mu_{p\hbar(u)}(\tau,\mathbf{x})(\{(u_1,u_2,\ldots,u_{A_u(\tau)},A_u(\tau)+1)\},1)\Big]x_u\Delta + o(\Delta) \\
&= (\tau,\mathbf{x}) + (\hat{\tau},\hat{\mathbf{x}}),
\end{aligned} \tag{S2.3}$$

where $\hat{\tau} = \tau \cup \{u(A_u(\tau)+1) : u \in \tau\}$ and

$$\hat{x}_u = \begin{cases} (a_{p\hbar(u)}(\tau,\mathbf{x}) - 1)x_u\Delta & u\in\tau \\ \mu_{p\hbar(u)}(\tau,\mathbf{x})x_{p(u)}\Delta & u\in\hat{\tau}\setminus\tau \end{cases} \tag{S2.4}$$

Then, we can observe that

$$\begin{aligned}
\left|a_{p\hbar(u)}(m_{(\tau,\mathbf{x})}(\Delta)) - a_{p\hbar(u)}(\tau,\mathbf{x})\right| &< \epsilon \\
&\Updownarrow \\
\left|a_{p\hbar(u)}((\tau,\mathbf{x}) + (\hat{\tau},\hat{\mathbf{x}})) - a_{p\hbar(u)}(\tau,\mathbf{x})\right| &< \epsilon \\
&\Updownarrow \\
\left|Da_{p\hbar(u)}(\tau,\mathbf{x})(\hat{\tau},\hat{\mathbf{x}}) + o(\Delta)\right| &< \epsilon \\
\left|\sum_{v\in\tau}\frac{\partial a_{p\hbar(u)}}{\partial x_v}(\tau,\mathbf{x})\hat{x}_v + o(\Delta)\right| &< \epsilon
\end{aligned}$$

and similarly

$$\begin{aligned}
\left|\mu_{p\hbar(u)}(m_{(\tau,\mathbf{x})}(\Delta)) - \mu_{p\hbar(u)}(\tau,\mathbf{x})\right| &< \epsilon \\
&\Updownarrow \\
\left|\sum_{v\in\tau}\frac{\partial \mu_{p\hbar(u)}}{\partial x_v}(\tau,\mathbf{x})\hat{x}_v + o(\Delta)\right| &< \epsilon
\end{aligned}$$

The partial derivatives are easily computed:

$$\frac{\partial a_{p\hbar(u)}}{\partial x_v}(\tau, \mathbf{x}) = \begin{cases} -\frac{1}{m_{p\hbar(u)}+1}\frac{\lambda_{p\hbar(u)}}{k_{p\hbar(u)}}\alpha_{p\hbar(u)p\hbar(v)} & v \in \tau, v \neq u \\ -\frac{1}{m_{p\hbar(u)}+1}\frac{\lambda_{p\hbar(u)}}{k_{p\hbar(u)}} & v = u \end{cases} \tag{S2.5}$$

$$\frac{\partial \mu_{p\hbar(u)}}{\partial x_v}(\tau, \mathbf{x}) = \begin{cases} -\frac{m_{p\hbar(u)}}{m_{p\hbar(u)}+1}\frac{\lambda_{p\hbar(u)}}{k_{p\hbar(u)}}\alpha_{p\hbar(u)p\hbar(v)} & v \in \tau, v \neq u \\ -\frac{m_{p\hbar(u)}}{m_{p\hbar(u)}+1}\frac{\lambda_{p\hbar(u)}}{k_{p\hbar(u)}} & v = u \end{cases} \tag{S2.6}$$

Finally leading to

$$\begin{gathered} \left| a_{p\hbar(u)}(m_{(\tau,\mathbf{x})}(\Delta)) - a_{p\hbar(u)}(\tau, \mathbf{x}) \right| < \epsilon \\ \Updownarrow \\ \left| -\sum_{\substack{v\in\tau \\ v\neq u}} \frac{1}{\mu_{p\hbar(u)}+1}\frac{\lambda_{p\hbar(u)}}{k_{p\hbar(u)}}\alpha_{p\hbar(u)p\hbar(v)}(a_{p\hbar(v)}(\tau,\mathbf{x})-1)x_v\Delta - \frac{1}{\mu_{p\hbar(u)}+1}\frac{\lambda_{p\hbar(u)}}{k_{p\hbar(u)}}(a_{p\hbar(u)}(\tau,\mathbf{x})-1)x_u\Delta \right| < \epsilon \\ \Updownarrow \\ \left| \frac{1}{m_{p\hbar(u)}+1}\frac{\lambda_{p\hbar(u)}}{k_{p\hbar(u)}}\Delta \right| \left| \sum_{\substack{v\in\tau \\ v\neq u}} \alpha_{p\hbar(u)p\hbar(v)}(a_{p\hbar(v)}(\tau,\mathbf{x})-1)x_v + (a_{p\hbar(u)}(\tau,\mathbf{x})-1)x_u \right| < \epsilon, \end{gathered} \tag{S2.7}$$

Analogously,

$$\begin{gathered} \left| \mu_{p\hbar(u)}(m_{(\tau,\mathbf{x})}(\Delta)) - \mu_{p\hbar(u)}(\tau, \mathbf{x}) \right| < \epsilon \\ \Updownarrow \\ \left| \frac{m_{p\hbar(u)}}{m_{p\hbar(u)}+1}\frac{\lambda_{p\hbar(u)}}{k_{p\hbar(u)}}\Delta \right| \left| \sum_{\substack{v\in\tau \\ v\neq u}} \alpha_{p\hbar(u)p\hbar(v)}(a_{p\hbar(v)}(\tau,\mathbf{x})-1)x_v + (a_{p\hbar(u)}(\tau,\mathbf{x})-1)x_u \right| < \epsilon, \end{gathered} \tag{S2.8}$$

which is equivalent, combining the two and isolating Δ, to

$$\Delta < \frac{\epsilon}{\left| \sum_{\substack{v\in\tau \\ v\neq u}} \alpha_{p\hbar(u)p\hbar(v)}(a_{p\hbar(v)}(\tau,\mathbf{x})-1)x_v + (a_{p\hbar(u)}(\tau,\mathbf{x})-1)x_u \right|} \left| \frac{m_{p\hbar(u)}+1}{\max\{m_{p\hbar(u)}, 1\}}\frac{k_{p\hbar(u)}}{\lambda_{p\hbar(u)}} \right|. \tag{S2.9}$$

Thus, taking Δ as the minimum of all possible values computed for each population $u \in \tau$, conditions (S2.1) and (S2.2) are guaranteed.

The error ϵ is to be scaled considering the measurement units of the parameters, which is well represented by $\sum_{u\in\tau}(a_{p\hbar(u)}(\tau,\mathbf{x}) + 1 + \mu_{p\hbar(u)}(\tau,\mathbf{x}))$, hence:

$$\epsilon = \epsilon_{rel} \cdot \sum_{u\in\tau}(a_{p\hbar(u)}(\tau,\mathbf{x}) + 1 + \mu_{p\hbar(u)}(\tau,\mathbf{x})).$$

References

1. Ethier SN, Kurtz TG. Markov Processes. Ethier SN, Kurtz TG, editors. Probability & Mathematical Statistics S.. Nashville, TN: John Wiley & Sons; 1986.
