# Supplementary material for: A new cancer progression model: From synthetic tumors to real data and back
Source: PLoS Comput Biol. 2026 Jun 24;22(6):e1013991. doi: 10.1371/journal.pcbi.1013991 (PMC13327523; doi:10.1371/journal.pcbi.1013991)
Supplement: S3 Appendix — A detailed analysis of the distributional properties of the simulated tumors for a wide-ranging choice of parameters. (PDF) [file pcbi.1013991.s003.pdf]

Supporting information

S3 Appendix: parameter exploration

This supplementary section reports some of the simulation experiments performed to explore the behaviour of the model under a broad range of parameter configurations. The goal is to make explicit how different assumptions shape the growth trajectories, clonal architecture, and mutational landscape generated by the model.

All simulations share a core set of global biological parameters to ensure consistency. While certain values are chosen for computational efficiency, they are designed to maintain the scaling laws of tumor growth. Specifically:

Carrying Capacity ($K = 10^6$): This value is chosen primarily for computational rapidity. While smaller than a clinical-stage tumor, it is sufficiently large to support complex population dynamics and large-scale clonal competition. Furthermore, the system is designed to be scalable; for instance, type 3-Limit evasion is analyzed in relative terms, exploring additional space ranging from $\frac{1}{10} times$ to $10\times$ the original K.

Baseline Proliferative Advantage ($s_{base} = 0.1$): The native population (we will refer to it as Wide Type WT) begins with a fixed selective advantage. This value is a matter of scaling relative to the cell cycle; the same ecological equilibrium could be reached by proportionally increasing cell lifespan or decreasing s. Again, we will analyze type 1-Deregulation of the proliferation program relatively to the baseline, exploring additional proliferative advantages from $\frac{1}{10}\times$ to $10\times$ the original s_{base}.

Cell Cycle Duration (4 days): We assume an indicative “cell life" of 4 days to provide a human-interpretable timescale. This allows us to map simulation steps to real-world time, making it possible to simulate several years of tumor progression in a way that is meaningful for clinical comparison. It does not affect the results once the proper adjustment are made, it only set the time unit.

Baseline Mutation Rate ($m_{base} = 2.66 \times 10^{-9}$): When the mutation rate is not set to zero, we use this value (per base pair per division) as it represents a known biological benchmark for cancer human cells [51].

Mutational Target Size (1000 bp): We assume a target of 1000 mutable base pairs. This is a reasonable abstraction that ensures a sufficient flux of mutations to observe diverse evolutionary trajectories without introducing unnecessary computational overhead.

Experiment 1 To substantiate the statements regarding the probability of a subclone to persist (i.e., not to go extinct) and the differences in final prevalence observed among different types of functional effects, we performed two sets of simulations mimicking simplified conditions: (1) acquisition of a new mutation during the expansive phase (before carrying capacity is attained), and (2) acquisition of a new mutation during the stable phase (after carrying capacity is attained). We ran 1000 simulations for each parameter configuration, starting with n wild-type cells with proliferative advantage $s = 0.1$ and one additional mutant cell (the newly acquuired mutation). In group (1), n was set to one quarter of the carrying capacity, while in group (2), it was set to the carrying capacity. The mutation rate was fixed to zero, to focus exclusively on the fate of the newly introduced subclone. The functional event conferred by the new mutation varied among three out of the four primary mechanisms: we excluded the type 2-Mutation burden augmentation, as it would have no effect under zero mutation rate.

Different parameter values were explored for each run. In particular, for functional events associated with type 1-Deregulation of the proliferation program, the proliferative advantage tested were chosen in relation with the selective advantage of the wild-type, running from one tenth to ten times it. For type 3-Limit evasion events, a similar reasoning was applied to the carrying capacity: additional space was let to vary between one tenth and ten times the original limitation. Lastly, type 4-Resource control parameters were explored in a range between -2 and 2, excluding those few combinations expected to result in explosion, see Fig J.

As a first step, we evaluated, for each parameter set and starting condition, how often the clone survived until the end of the simulation, across the 1000 replicates, and whether the survival frequency was different under the two acquisition scenarios (expansive phase vs. stable phase). Results are shown in Fig A. The squares with significant p-values represent, for different choices of the parameters and of the type of primary mechanism, the experiments where the survival probability of the subclone is different in the two acquisition scenarios (expansive phase vs. stable phase). The plot shows that such differences are always significant for type 0-Null effect events (passenger mutations) and type 1-Deregulation of the proliferation program events. In these cases, the test for the null hypothesis of equal proportions consistently showed a highly significant rejection across all the parameter values that were considered. This indicates that, for these functional mechanisms, the acquisition during the expansive phase makes the emergence of a persistent subclone significantly more likely. For type 4-Resource control events, the model displayed a marked sensitivity to the susceptibility parameter. Overall, the probability of subclone persistence tended to increase as susceptibility decreased, regardless of the acquisition phase. However, the magnitude and direction of the difference between expansive and stable acquisitions depended on the susceptibility range. A first cluster of significant differences was observed around susceptibility values close to 1, which corresponds to the near-neutral case. In this range, the behaviour resembled that of passenger mutations: earlier acquisition (during expansion) conferred a higher survival probability. Interestingly, for lower susceptibility values, the trend reversed: acquisition during the stable phase was more likely to lead to persistence. These cases, however, are rare and biologically less plausible, as a negative susceptibility would imply an ability to extract resources from neighbouring cells rather than from a newly acquired source. Finally, occasional but inconsistent differences in survival proportions were also detected for some type 3-Limit evasion functional events.

We investigated also whether the survival probability of each experiment was significantly different than the survival probability of type 0-Null effect (passenger) mutations. The results are shown in Fig B. The 4-resource control functional events show clear differences compared with passenger mutations across all experiments in which the parameters are set to values different from neutrality, i.e., not equal to 1. A similar remark applies to the 3-limit evasion functional events. The 1-deregulation of the proliferative program functional events exhibit survival dynamics that differ from those of passenger mutations only when they are acquired during the early phases of mass expansion; otherwise, any proliferative advantage does not necessarily translate into higher survival probabilities.

In this same setting, we analyzed as well the distribution of the final subclone mass among the survived simulated tumors, focusing on the proportion of total mass gained. To this end, we applied a VAF-density-like approach, assuming that each mutation lies on a single allele of the carrier cell. Accordingly, the Variant Allele Frequency (VAF) was estimated as half the final fraction of cells carrying the mutation. We then tested whether the VAF distributions associated with each mutation type differed between the two acquisition scenarios (expansive vs. stable phase) at fixed parameter values, using a Kolmogorov–Smirnov test (`ks.test()` in `R`). The same test was also used to compare

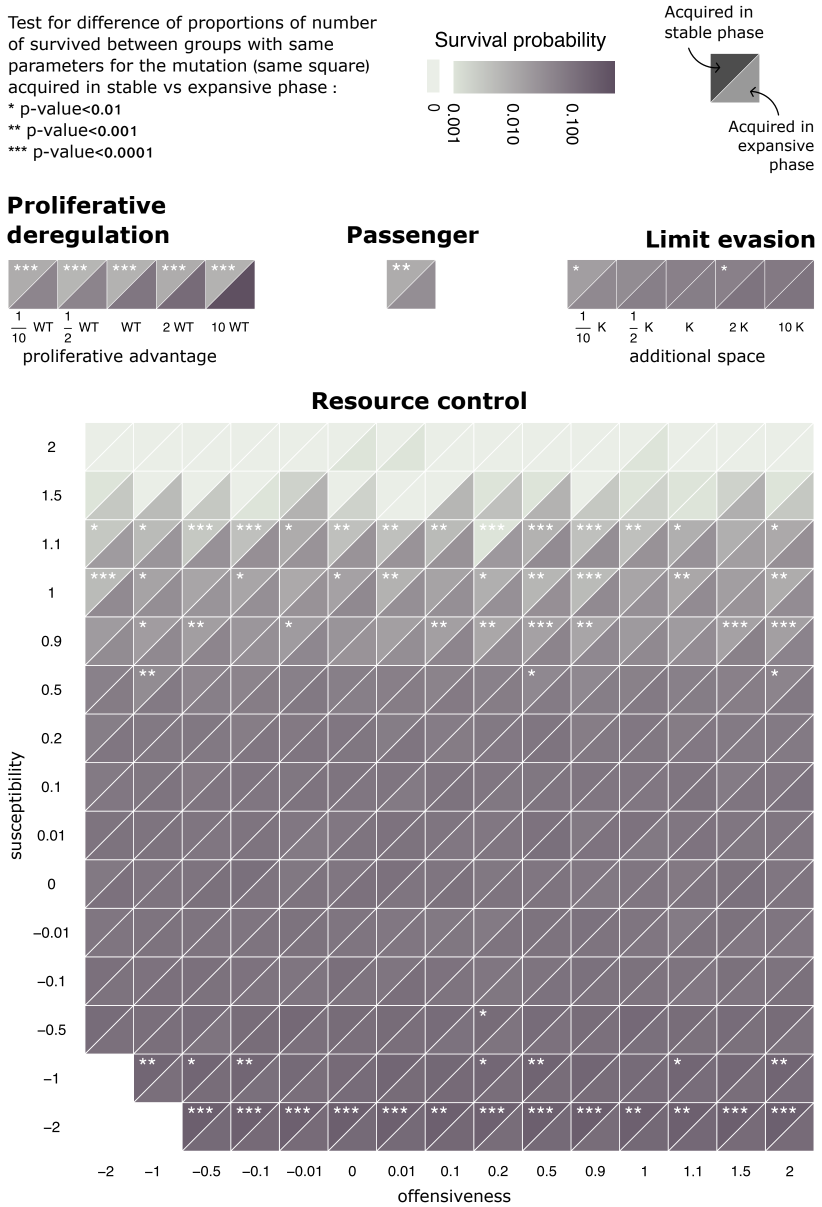

Fig A. Proportion of surviving subpopulations across 1000 simulation runs, grouped by type of the primary mechanism of the new mutation and by time of acquisition. Each square represents a specific phenotype and is divided diagonally: the bottom-right half refers to early acquisitions during the expansive phase, and the top-left half to late acquisitions during the stable phase. The color gradient indicates the proportion of surviving clones (log scale). The significance value shown in each square refers to the test for the equality of survival probabilities among the clones born during expansive phase and the clones born during the stable phase.

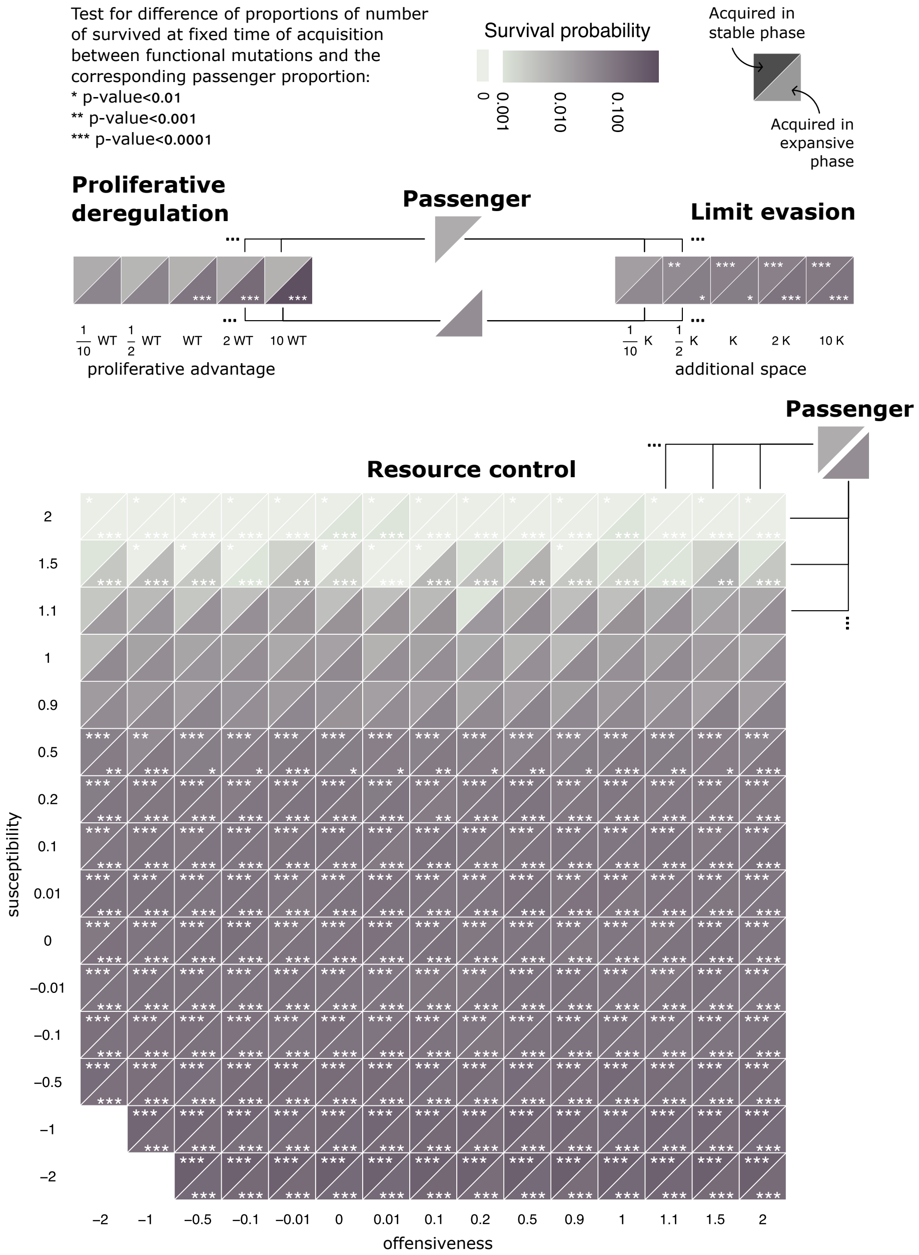

Fig B. Proportion of surviving subpopulations across 1000 simulation runs, grouped by type of the primary mechanism of the new mutation and by time of acquisition. Each square represents a specific phenotype and is divided diagonally: the bottom-right half refers to early acquisitions during the expansive phase, and the top-left half to late acquisitions during the stable phase. The color gradient indicates the proportion of surviving clones (log scale). The significance reported in each half square is referred to the difference of survival probability of the clone and the survival probability of type 0-Null effect (passenger) mutations.

the VAF distributions of non-neutral mutations to those of passenger mutations acquired during the same phase. The analysis of the final prevalence of the survived subclones confirmed and extended the results obtained from survival probabilities. Passenger mutations produced consistently low VAFs, independently of the acquisition phase: the corresponding distributions did not differ significantly between expansive and stable conditions, see Fig C. This reinforces that purely neutral mutations, although occasionally able to persist, cannot give rise to substantial fractions of the tumor mass and therefore fail to account for the observed intratumor heterogeneity. Type 1-Deregulation of the proliferation program mutations display a clear dependence on the time of acquisition. The Kolmogorov–Smirnov test revealed statistically significant differences with markedly higher VAFs for subclones that were born during the expansive phase of growth, but only when the proliferative advantage exceeded that of the wild type, see Fig C. Moreover, there is no observable difference between the VAFs of neutral mutations and the VAFs of the type 1 whenever acquired during the stable phase, while a significant shift to larger VAFs values is observed for the acquisitions during the expansive phase, see Fig D. Nevertheless, even under these conditions, the typical VAFs remained modest, and only the extreme case of a tenfold advantage produced subclones with substantial prevalence. This indicates that while early acquisition enhances the chance of expansion, deregulation of proliferation alone is generally insufficient to generate dominant clones once the system approaches the carrying capacity. For type 3-Limit evasion functional events, no consistent differences between the two acquisition timings were detected, in line with their mechanism of action, which affects the total space available rather than the growth rate, see Fig C. However, the VAFs resulting from this kind of mutations were higher than those of passengers acquired in the same phase, provided a large enough additional space conferred by them, see Fig D. If acquired in stable phase it is enough an additional space half the carrying capacity to observe a rise in the prevalence compared to the neutral case, while if acquired during the early expansive phase the additional space provided needs to be at least two times the carrying capacity to actually make a difference. This is consistent with the idea that these kind of events play a major role when the mass of the tumor has reached its limit size. The type 4-Resource control mechanisms, instead, exhibited the richest spectrum of behaviors: the KS tests identified significant differences both between acquisition phases and relative to passenger mutations, mostly depending on the susceptibility parameter, see Fig C and Fig E for a detailed view. For values near the neutral case (susceptibility≈ 1), the trend is not different to that of passengers, see Fig F. As susceptibility decreased, however, VAFs became substantially larger, and for strongly negative values, acquisitions during the stable phase even outperformed those during expansion, see Fig C. Overall, these patterns highlight that only mutations that alter ecological constraints by modulating access to space or resources can consistently produce subclones that can occupy a significant fraction of the tumor mass and that can be major actors during the stable phase of the tumor evolution.

Experiment 2 To assess how functional events and parameter choices influence the dynamics in a less constrained setting, we repeated the simulations without conditioning the acquisition of the mutational event, and without enforcing the uniqueness of the emerging subclone. Each simulation started from a single cancer cell carrying a proliferative advantage of 0.1. New subclones could arise freely, but all of them were linked to the same functional event. For each functional event (using the same values considered in **Experiment 1**), we ran 100 independent simulations.

Unlike in the previous setting, here multiple subclones can emerge, so we can apply the sampling procedure described in Fig 2 to derive allele frequencies, which more

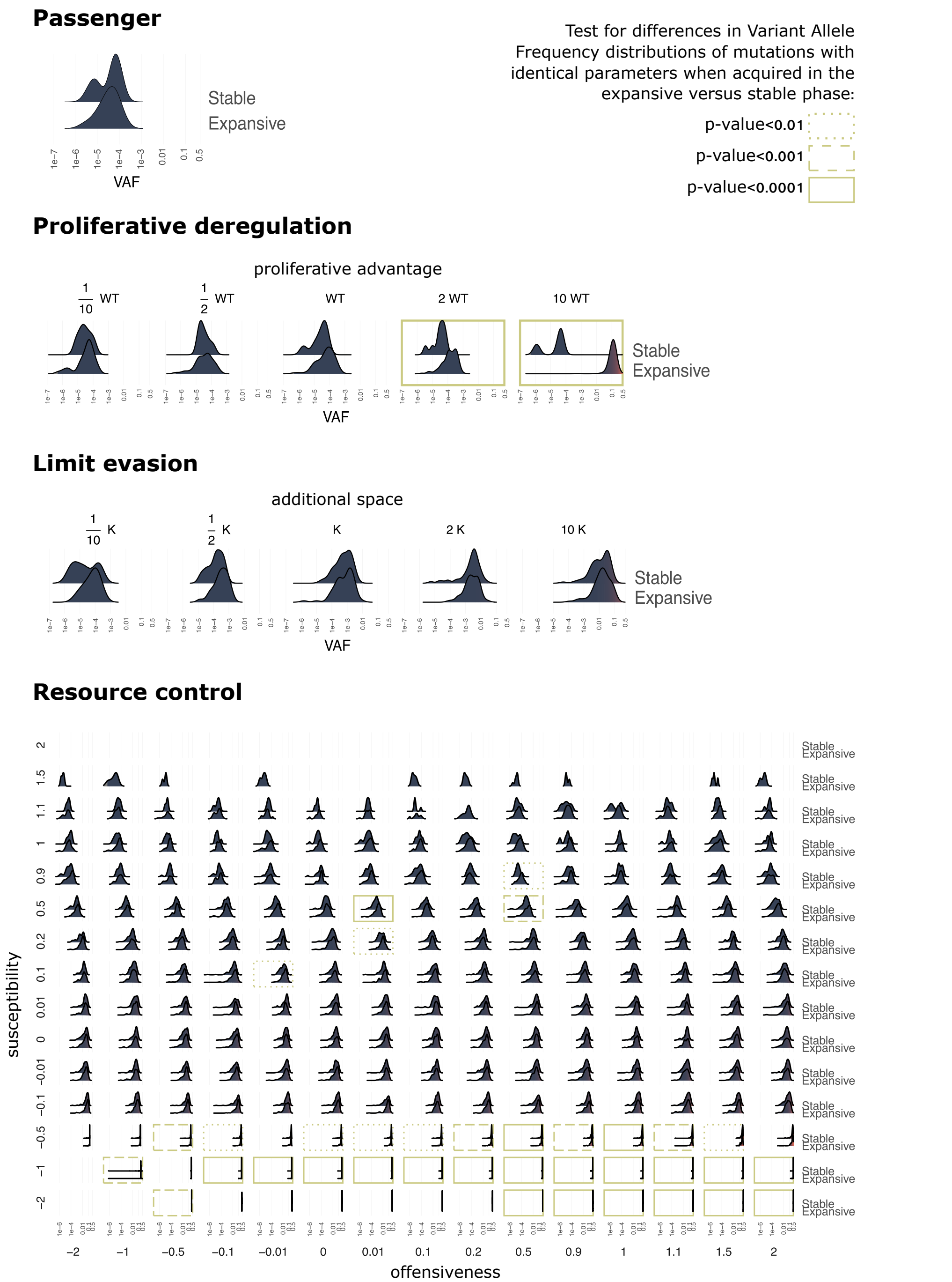

Fig C. Probability density of allele prevalences in surviving subclones. Each plot displays the VAFs of mutations sharing the same functional effect and compares two distributions: mutations acquired during the early expansive phase and those acquired during the late stable phase. Plots are outlined when the difference between the two distributions is significant according to a Kolmogorov–Smirnov (K–S) test. The x-axis is shown on a logarithmic scale to allow comparison across event types characterized by markedly different VAF ranges.

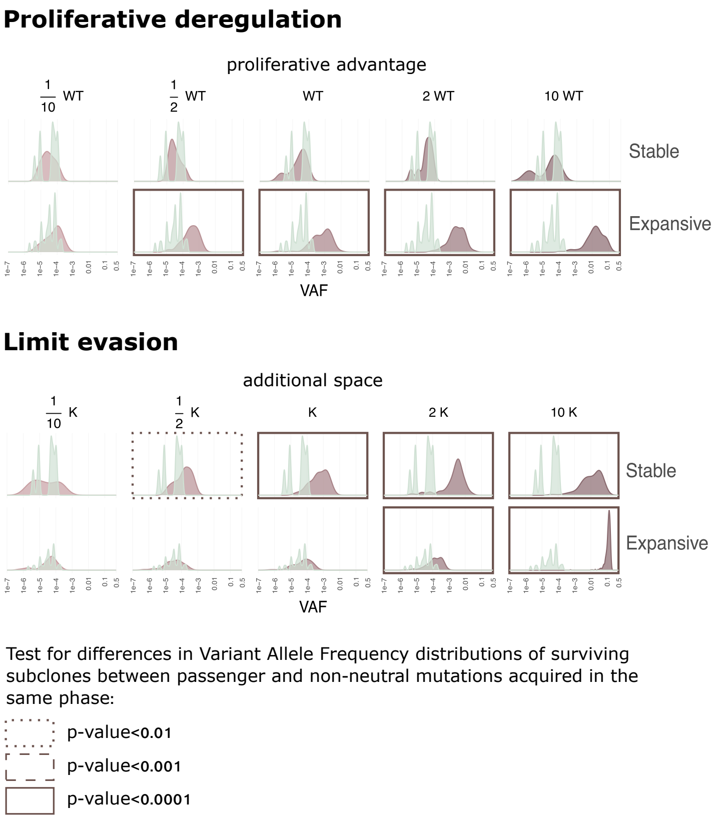

Fig D. Probability density of allele prevalences in surviving subclones with type 1 mutations providing a proliferative advantage and with type 3-Limit evasion mutations providing the ability to evade environmental constraints. In each plot, the distribution of the considered event (in mauve) is compared with that of passenger mutations acquired in the same phase (mint blue). Plots are outlined when the difference between the two distributions is statistically significant. The x-axis is shown on a logarithmic scale to accommodate the wide range of VAF values.

closely reflects how VAFs are obtained in real sequencing. To simulate sequencing, we subsampled 1% of the final synthetic tumor mass using a proximity-based approach informed by the Relative Abundance Muller plots: a value between 0 and 1 was sampled, and cells from populations in its neighborhood were included. Reads were then randomly amplified (uniformly), and for each mutation a depth of coverage (DP) was drawn from a distribution based on TCGA data. Finally, a number of reads up to the sampled DP was selected for each mutation. Additionally, in the setting of **Experiment 1** we could directly estimate the survival probability of each subclonal type. This is no longer possible here. However, because all simulations use the same mutation rate and the same number of mutable sites, we expect the number of subclones arising during tumor growth to be comparable across parameter sets. Therefore, if a sequencing-like sampling reveals different numbers of detectable mutations, this indicates differences in subclone survival: fewer detected mutations imply a higher extinction rate. The dotplots in Fig G illustrate this, while also showing how the allele frequency distributions vary across scenarios. We again observe that passenger mutations rarely persist and that are associated with low VAFs. The same holds for type 1-Deregulation of the proliferation program mutations, unless the proliferative advantage they confer exceeds by far that of the founding driver. Note that this corresponds to an extreme scenario: especially assuming a tenfold stronger proliferative effect is not realistic and is inconsistent with previous models that consider proliferation alone. Still, it is worth noting that the ability of a subclone to generate persistent and invasive lineages increases as the proliferative advantage does, eventually allowing it to overtake the original wild-type population.

Mutations associated with the ability to access new resources or space (i.e. type 3-Limit evasion events) are consistently observed in large numbers and with high prevalence, provided that the newly available resource allows at least one half of the carrying capacity in additional cells. On the other hand, the analysis of type 4-Resource control mutations that modify how resources are shared and used among clones offer new insights in this set of experiments. The influence of the susceptibility parameter is again evident, and the threshold at $\nu = 1$ confirms a passenger-like behavior: susceptibility values close to 1 correspond to very few detected mutations at low frequencies, an effect even stronger for values above 1. This is reasonable, as such cases correspond to subclones that are more sensitive than the surrounding tumor tissue to competitive pressure. For susceptibility values lower than 1, distinct VAF density profiles emerge, with higher peaks corresponding to higher offensiveness values. This trend becomes even clearer in the ridgeplots, see Fig H. Within each column, comparing the density distributions reveals a visible tendency for the VAFs to concentrate around 0.5 in the upper panels, whereas the lower ones display wider distributions with an additional peak near 0. This pattern is largely explained by the number of mutations detected: as shown in Fig I, the number of mutations identified by simulated sequencing decreases as susceptibility increases, while it rises with increasing offensiveness. Again, the value 1 marks a threshold for offensiveness as well, distinguishing genuinely offensive from cooperative behavior. Regardless of susceptibility, two distinct clusters are observable, one below and one above this threshold. Further analysis indicates that the number of low-VAF subclones remains stable across all parameter combinations; what changes is the number of high-VAF subclones detected, see Fig J. This explains the VAF distributions in the ridgeplots Fig H: those with a single, more centered peak have not lost the low-VAF mutations, their number stays the same, but they become proportionally fewer compared to the growing number of high-VAF subclones, shifting the probability mass toward higher values. Overall, the analysis of type 4-Resource control functional events suggests that a clone's ability to withstand pressure from surrounding cells (susceptibility) determines its fate at a broad level, effectively marking

the boundary between neutrality and selection. In contrast, the harmful potential (offensiveness) acts at a finer scale, influencing competition only on the advantageous side of that boundary, and enhancing the subclone's ability to overtake its siblings and occupy most of the available space. The effect produced by the offensiveness, already visible in Fig E, becomes even clearer when it is analyzed at the community level, allowing several subclones of the same type to emerge, and when applying the VAF sampling procedure that mimics real sequencing experiments.

This last factor, in particular, seemed to be important and interesting to investigate. To assess how much noise is introduced into VAF data by the sequencing process itself, we considered the neutral case and repeated the sampling procedure 100 times, then compared the sampled values with the theoretical allelic prevalences of the mutations. The sampled allele frequencies were found to be both strongly inflated and more dispersed than their corresponding pure prevalences, see Fig K. We further examined this discrepancy by testing several scenarios that included only passenger mutations but varied in the number of base pairs considered. Regardless of the panel size, the shift remained consistent.

Experiment 3 (*discussed in the main paper*) In the previous experiments we restricted the analysis to first generation subclones, allowing only a single effect to be summed to the wild-type. Now we relax this constraint. We still keep simulations segregated by functional effect, but we now allow each simulated tumor to accumulate up to six events of the same type, using identical primary mechanisms and identical parameter values. This distinction is essential: a list of six identical functional events is not equivalent to a single event. In the single event case, higher generation subclones gain no additional advantage over their parents, because a given biological mechanism can be disrupted only once: multiple mutations targeting the same process do not stack. Conversely, when the functional event list contains several distinct mutations that produce the same class of effect, their contributions accumulate: each additional hit breaks a different component of the same biological system, and their selective effects add up. Under these conditions, mutational paths branch and deepen, making it possible for later generation lineages to follow a different fate from their ancestors.

The simulation begins with a single cell ($s = 0.1$). Mutations are acquired during tumor development ($m_{base} = 2.66 \cdot 10^{-9}$ across 1000 bp). In this experiment we restricted the parameter space to the biologically and computationally relevant subset identified in the previous analyses. For type 1-Deregulation of the proliferation program, we excluded cases in which the added advantage is weaker than that of the wild type, as these consistently behaved like passenger mutations. We retained only advantages equal to 1, 2 and 10 times the wild-type value ($s \in \{0.1, 0.2, 1.0\}$). For type 3-Limit evasion, we focused on mutations that provide at least one full carrying capacity of additional space, since smaller increments had negligible impact, i.e. $k \in \{10^6, 2 \cdot 10^6, 10^7\}$. For type 4-Resource control, we limited the analysis to the "resistant" regime, where the emerging subclone is better able to withstand competitive pressure. This corresponds to fixed offensiveness equal to 1 and susceptibility values between 0 and 1, in particular $\nu \in \{0.1, 0.2, 0.5, 0.7\}$. The choice is motivated by biology, as these configurations correspond to interpretable competitive strategies, and by computation, since earlier results showed that susceptibility is the dominant parameter shaping resource based advantages. Each specific parameter configuration was tested across *100 independent runs* to capture the emergence of complex hierarchies. Simulations are stopped either after 5 years, or if they reach a maximum size M. For each parameter configuration three different scenarios of maximum sizes have been considered:

- $M = \frac{1}{10}K$, i.e. one tenth of the carrying capacity, to keep the evolution in the *expansive phase*;

- $M = 2K$, i.e. two times the carrying capacity, to explore *stable phase* early dynamics;
- $M = 10K$, i.e. ten times the carrying capacity, to explore *stable phase* late dynamics.

This experiment is commented in the main paper under the "Results-Exploration of evolutionary paradigms" section of the main text.
Run times. We used this experiment to compile an indicative report of run times, as it represents the most complete and non-trivial experiment conducted under a controlled scenario. The experiment was run on an HPC infrastructure equipped with an OmniPath network and 2 $\times$ Intel Xeon CPU E5-2697 v4 processors and holding 125GB RAM. Simulations were executed in parallel (i.e., different simulations running concurrently), while run times were recorded on a per-simulation basis. On average, each simulated tumor mass requires 1.36 minutes (sd 1.04 min). However, run times are strongly influenced by the model parameters. In particular, the maximum size M has a clear impact, which is expected since it acts as a stopping criterion: the smaller M, the earlier the simulation terminates. Specifically, the average run time is 7.98 seconds (sd 2.78 s) for $M = 0.1K$, 1.72 minutes (sd 38.2 s) for $M = 2K$, and 2.24 minutes (sd 39.5 s) for $M = 10K$. This also explains the differences observed in Fig L across functional effects: longer runs are typically associated with parameter configurations that are less likely to reach the stopping threshold (see Fig 4).

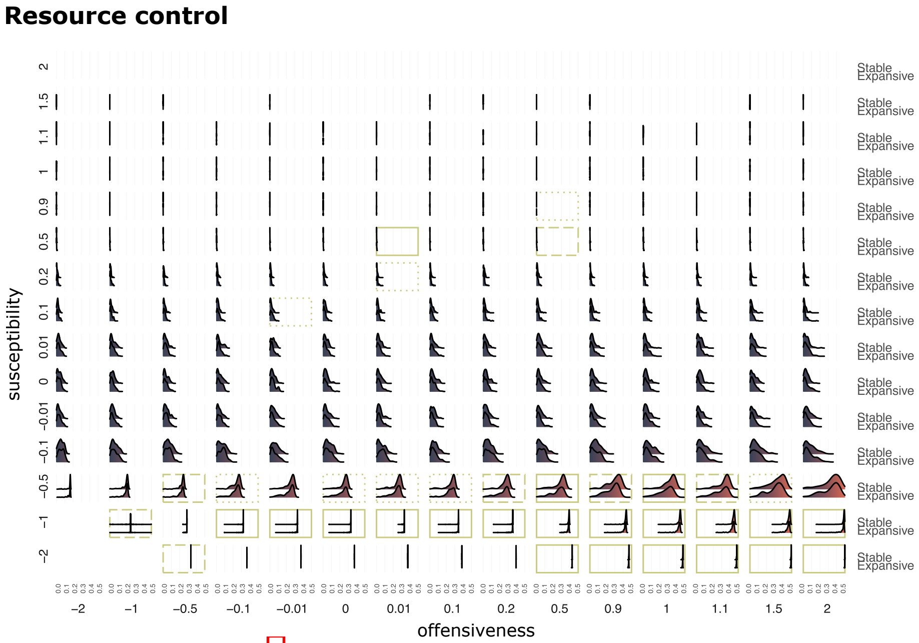

Fig E. Focus on type 4-Resource control functional events. The content is identical to that of Fig C, but the x-axis is displayed on a linear scale to better highlight internal differences.

Resource control

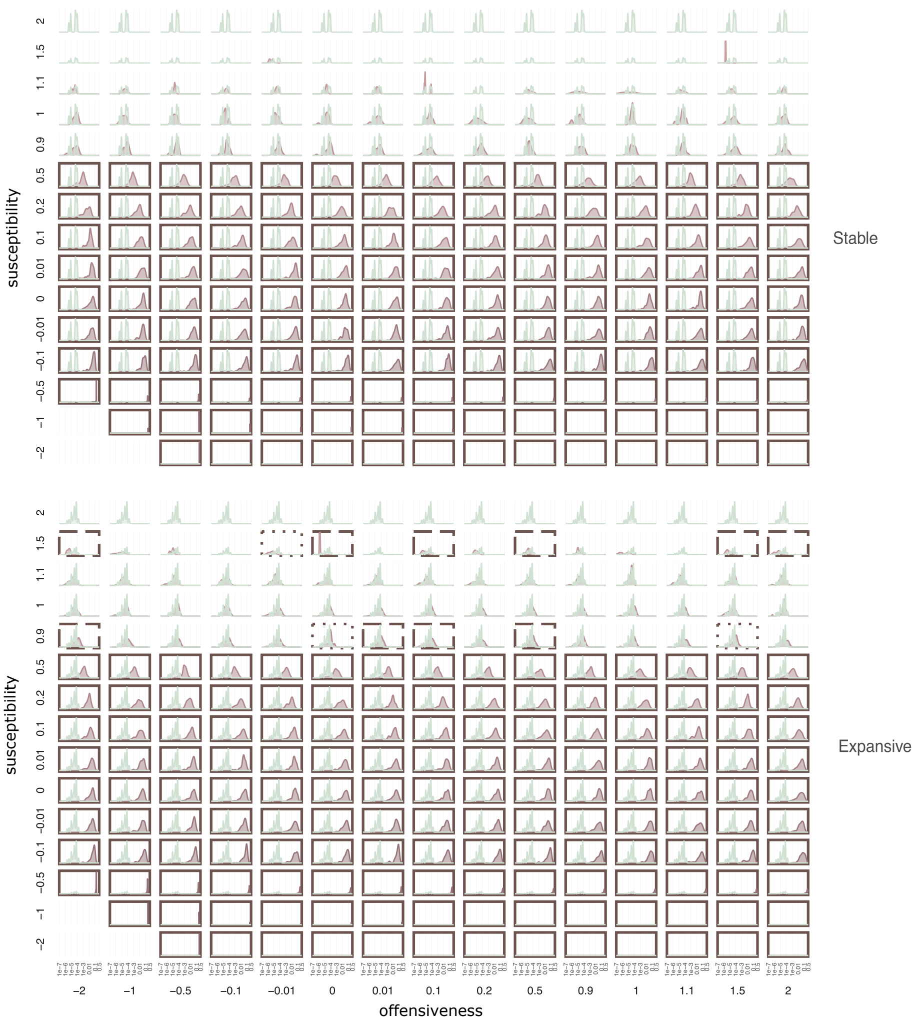

Fig F. Probability density of allele prevalences in surviving subclones with type 4-Resource control mutations. In each plot, the distribution of the considered event (in mauve) is compared with that of passenger mutations acquired in the same phase (mint blue). Plots are outlined when the difference between the two distributions is statistically significant. The x-axis is shown on a logarithmic scale to accommodate the range of VAF values.

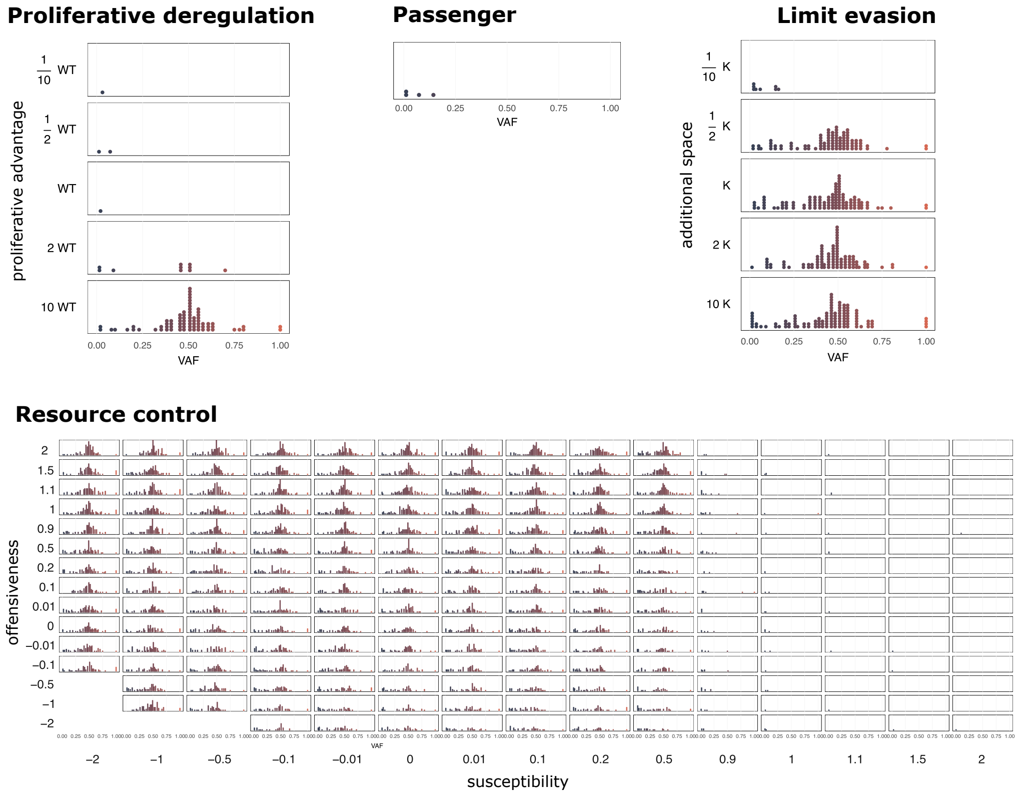

Fig G. Dotplots of the sampled VAFs for each tumor, grouped by primary mechanism of the functional effect. Each dot represents a sampled mutation, and dots are stacked in a histogram-like fashion to illustrate their distribution.

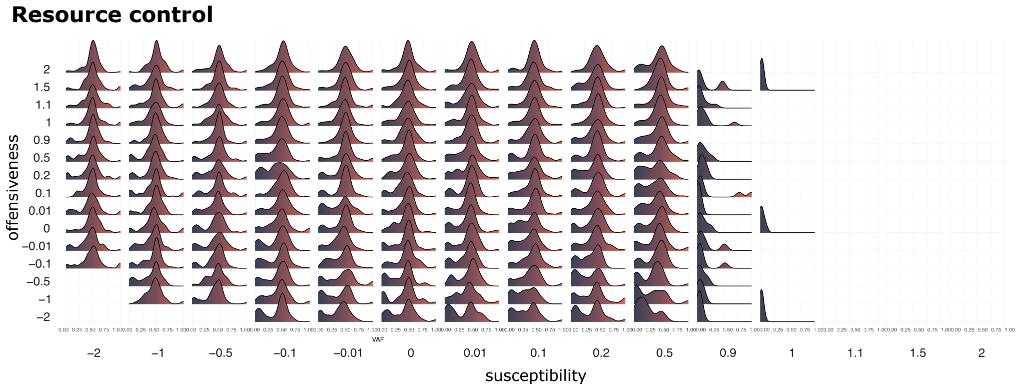

Fig H. Ridgeplot of the density of sampled allele frequencies for subclones with a type 4-Resource control functional effect. Each density curve corresponds to a specific combination of offensiveness and susceptibility parameters associated with the mutation.

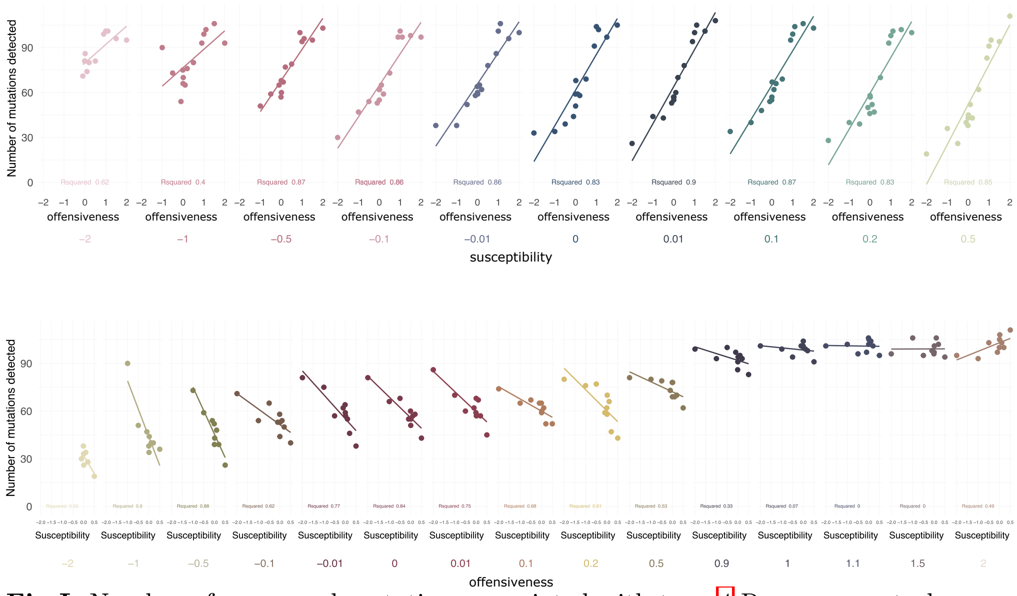

Fig I. Number of sequenced mutations associated with type 4-Resource control functional events as a function of the parameters offensiveness and susceptibility. Susceptibility values of 0.9 or higher were excluded due to insufficient data.

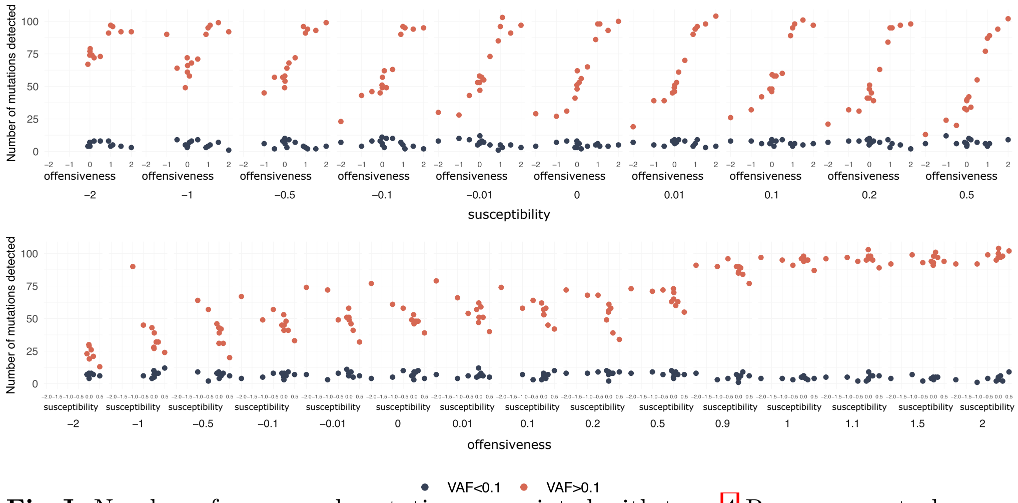

Fig J. Number of sequenced mutations associated with type 4-Resource control functional events as a function of the parameters offensiveness and susceptibility. Mutations are divided into two groups, colored differently: the red dots include only low-VAF mutations and the blue dots include high-VAF mutations (threshold 0.1). Susceptibility values of 0.9 or higher were excluded due to insufficient data.

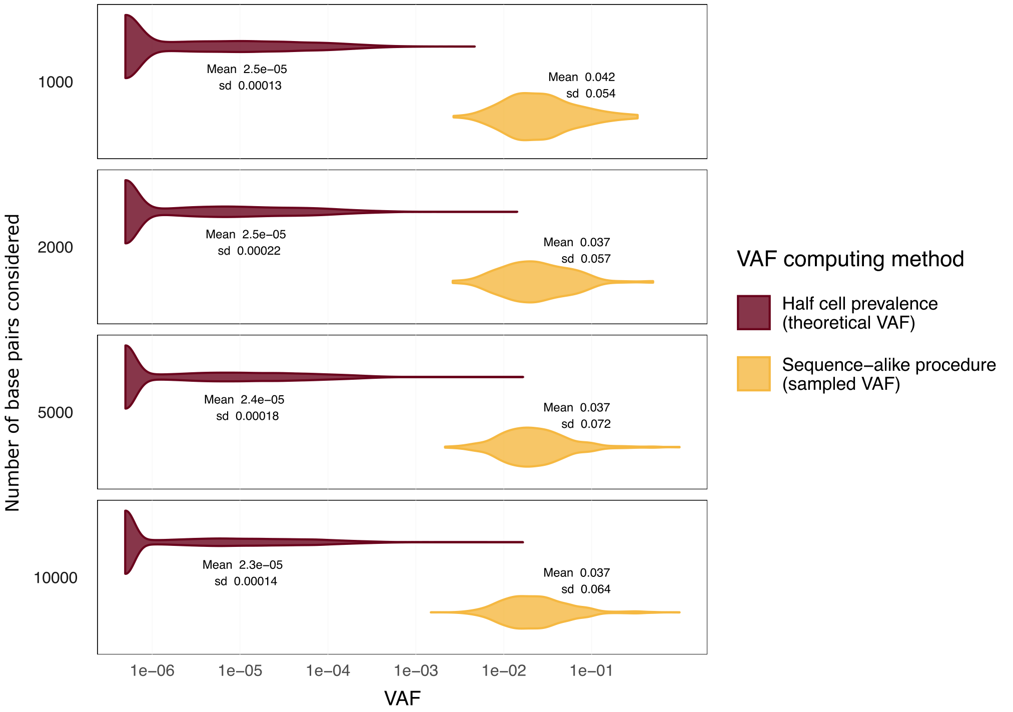

Fig K. Violin plots of VAF values comparing two approaches: the first derives VAFs by simply dividing the cell prevalence of each mutation by two, while the second simulates sequencing, including extraction of a fraction of the tumor mass, random amplification of reads, and random selection up to a randomly chosen coverage.

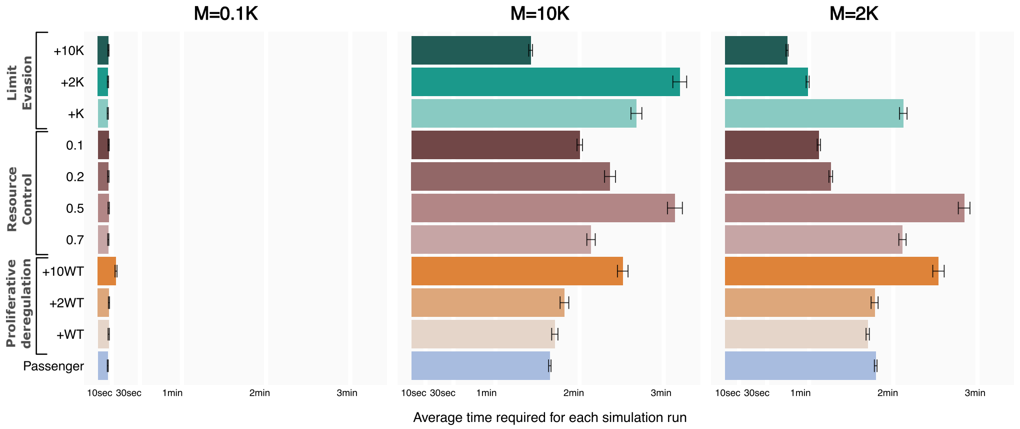

Fig L. Average running time (among the 100 independent runs) of each parameter configuration. The error bars correspond to 95% confidence intervals.

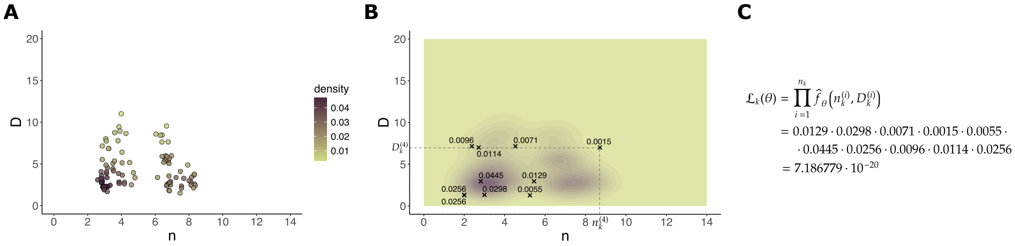

Fig M. Procedure for evaluating the likelihood of a parameter set θ given a real tumor dataset k. **A**. Each dot represents a simulation run with parameters θ; their scatterplot allows the construction of a bi-dimensional density. **B**. The corresponding density estimate (gradient background) is shown. Each real data point ($\times$) of dataset k is mapped onto this plane, and its probability density is read from the kernel estimate (label of the points). **C**. The product of these densities yields the likelihood of observing the real dataset under the simulated distribution. The higher the likelihood, the more consistent the parameter set is with the real data.

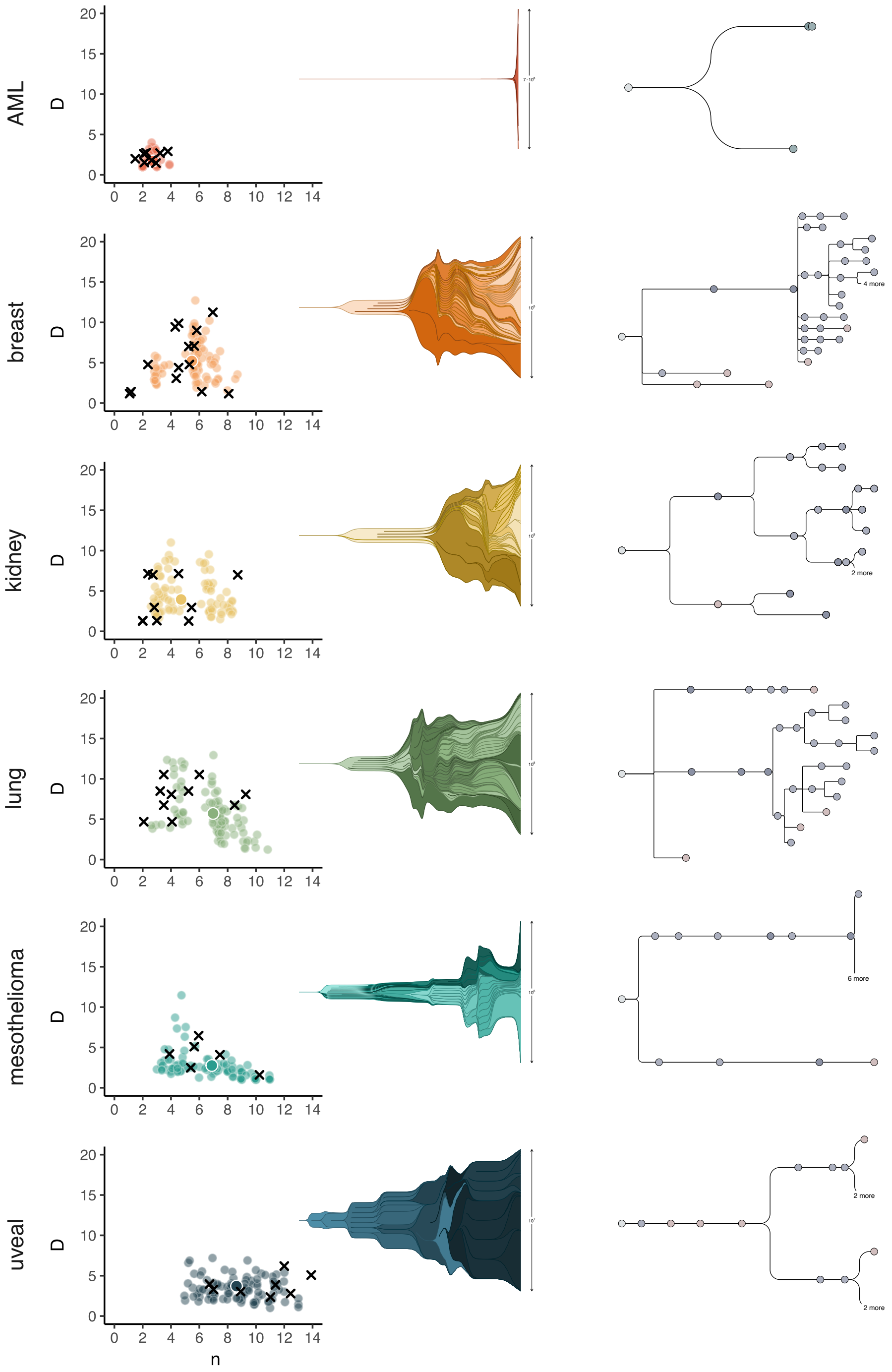

Fig N. For each cancer type, the maximum likelihood parameter set is shown, and the centroid of the corresponding distribution in the (n, D) plane is highlighted as a representative synthetic tumor. This representative tumor is shown alongside its Muller plot and the corresponding evolutionary tree. The evolutionary tree includes only mutations that persist until the end of the simulation and reach a final prevalence of at least 0.01. Tree colors indicate the associated functional events.

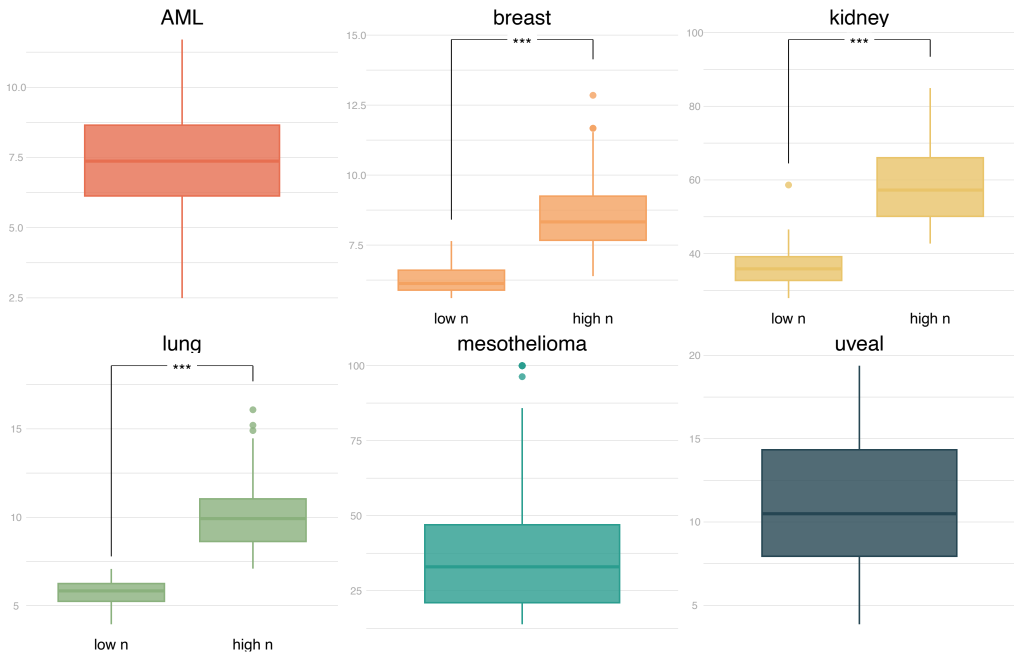

Fig O. Boxplots of the times to reach detection size, for each synthetic cancer type plotted in Fig 6. For breast, lung and kidney cancer types, the times have been divided in the two groups low-n and large-n. Significance (p-value < 0.0001) for the t-test for mean differences between the two groups are represented with triple stars.

References

1. Noble R, Burri D, Le Sueur C, Lemant J, Viossat Y, Kather JN, et al. Spatial structure governs the mode of tumour evolution. Nature Ecology & Evolution. 2021;6(2):207–217. doi:10.1038/s41559-021-01615-9.
