# Supplementary material for: A new cancer progression model: From synthetic tumors to real data and back
Source: PLoS Comput Biol. 2026 Jun 24;22(6):e1013991. doi: 10.1371/journal.pcbi.1013991 (PMC13327523; doi:10.1371/journal.pcbi.1013991)
Supplement: Attachment — Submitted filename: Response to reviewers.pdf [file pcbi.1013991.s004.pdf]

Dear Editor,

We are pleased to submit the revised version of our manuscript, entitled “A new cancer progression model: from synthetic tumors to real data and back”, together with our detailed responses to the reviewers’ comments. We are grateful to you and to the reviewers for the time and care devoted to the evaluation of our work. Your thoughtful and constructive comments have helped us improve the manuscript substantially. We are deeply thankful.

We addressed all the points raised by the reviewers. As a general and introductory comment, we can say that:

1. We have re-organized the manuscript including in the main text a new section “Materials and methods – The cancer progression model – The phenotype as the model parameters manager”, in which we provide the complete description of the model and its parameters. This section includes both a clear mathematical formulation of the model in all its details, and the biological interpretation of the parameter values.
2. We have included new references, we discuss them in the section “Discussion” in which we contextualize our model with respect to the literature in the field. Moreover, we improved the discussion about the differences between our model and the spatially segregated models.
3. We worked on the description of the figures and their captions, including the values of all the involved parameters for a clear understanding of all experimental results.
4. We added in the main text a new set of experiments dedicated to understanding the distributional properties of the simulated tumors. Those experiments have been included to the revised section “Results – Exploration of evolutionary paradigms”. The goal is to give to the reader a good understanding of the characteristics of the simulated tumors grown with the model. Each type of functional event is considered separately and the different effects are analyzed and described with their biological interpretation.
5. We created a github repository https://github.com/qBioTurin/Insite containing the R package Insite which includes the code for reproducing all the experiments and the figures of the paper. We corrected the technical errors on the graphical interface we designed to run explorative experiments with our simulator, https://github.com/qBioTurin/Insite_Interface. Moreover we created a Zenodo repository for all the simulated tumors included in the paper, https://zenodo.org/records/19821839. Now, all the results illustrated in the paper are reproducible.

In the following of this letter, the responses to each reviewer’s comments are reported. The reviewers’ original comments are reproduced in *yellow italics*, the authors’ responses are presented in plain black text, and excerpts from the manuscript are shown with gray background. All the reported line numbers, figure numberings and equation numberings are referred to the clean copy of the revised manuscript.

Sincerely,

Roberta Sirovich

—-------

Reviewer #1:

Authors introduce a very interesting evolutionary and ecological modeling framwork which consolidates many ideas from previous agent-based modeling papers in cancer, as well as conceptual ideas from the cancer hallmarks manuscripts. The figures are very nice, and the methods are very nice, however the results only show a small and incomplete picture that is unlikely to be reproducible. A new version of the manuscript should work harder to explain the figures, show how the results are generalizable beyond a single simulation, and to report the parameters used in each simulations.

We thank the referee for the careful reading of the manuscript and for the constructive comments and suggestions. We are submitting a revised version of the manuscript following the suggested recommendations. In particular:

1. Figures: we worked on the captions, to ensure that they include a clear description of the plot and the values of all the parameters involved. We worked on the text that describes and comments the figures, in order to give a better explanation of the results illustrated.
2. Beyond single simulation: we completely revised the section "Results/Exploration of evolutionary paradigms" adding a new set of experiments that are illustrated in the new Fig. 4. The goal is to illustrate at a tumor population level (overcoming the single tumor description) the main emerging abilities of the proposed model. Moreover, we better refer the reader to the supplementary section "S3 Parameter exploration" which contains a detailed analysis of the distributional properties of the simulated tumors for a wide-ranging choice of parameters.
3. Reproducibility: we extended the publicly available resources associated with the manuscript. Specifically, we now provide: 1. a Zenodo repository (https://doi.org/10.5281/zenodo.19821839) containing the complete raw outputs of all simulations used in both the main manuscript and the Supplementary Materials; 2. a GitHub repository Insite (https://github.com/qBioTurin/Insite.git) containing the full simulation and analysis package, example scripts illustrating how to run simulations and perform downstream analyses, the processed datasets derived from the raw simulation outputs, the complete code required to reproduce all figures in the manuscript from the processed data, the code required to regenerate the processed datasets starting from the raw simulation outputs deposited on Zenodo, a Docker image (distributed through DockerHub) with the package and all required dependencies pre-installed to facilitate reproducibility; 3. the previously available GitHub repository Insite_Interface (https://github.com/qBioTurin/Insite_Interface.git) providing the graphical user interface, which builds on top of the Docker image of the simulator and remains available as a user-friendly exploratory tool for running and analysing example simulations. The full simulation outputs, processing pipeline, and figure-generation workflow are therefore now publicly available.

Responses to each comment are reported below. The reviewers' original comments are reproduced in *yellow italics*, the authors' responses are presented in plain black text, and excerpts from the manuscript are shown with gray background. All the reported line numbers, figure numberings and equation numberings are referred to the clean copy of the revised manuscript.

1. Line 115: "A single phenotype can be determined by different genotypes" – is "determined" the right word here?

We modified the sentence as "A given phenotype may arise from different genotypes", lines 114-115.

2. Line 184: "a" repeated twice, "a a boost"

We corrected the typo, line 169.

3. Equation 4 isn't sufficiently described – it's not clear to me if this is a size-dependent branching process, or a variation with included phenotypic evolution. Secondly, please define SDBD in equation 4. Should this be SDBP?

We modified the description of Eq. 4 from line 247 to line 266 stressing that the model is a size-dependent branching process and that the dependency on the population size is encoded in the parameters $a_{ph(u)}$ and $b_{ph(u)}$ which are both phenotype associated and size dependent. We also defined SDBD which stands for Size-Dependent Birth and Death process.

> **The population evolution model** For each genotype $u \in \tau$, we associate $\sigma_u \geq 0$, the birth time of u, and $(X_u(t), t \geq \sigma_u)$, the number of cells with genotype u at time t, i.e. the clone.
>
> We make the following choices. The process determining the size of the cell population $(X_u(t), t \geq \sigma_u)$ is modeled as a *size-dependent birth and death process*. The theory of size-dependent branching processes, which extends classical branching models by allowing reproduction and survival rates to depend on population size, originated in the mid-20th century and includes contributions from mathematicians such as Klebaner and Jagers, see [46], [47], [48].
>
> Despite the name, size-dependent branching processes do not satisfy the classical branching property, namely that "each individual evolves independently into a branching process identically distributed to all the others". Indeed, the law governing a size-dependent branching process depends on the population size, making it impossible for an individual evolving within a population to follow the same law as an isolated individual generating the process alone.
>
> Recalling the idea that the functional mechanisms of a clone are completely determined by its *phenotype*, the birth and death rates of each population, $a_{ph(u)}$ and $b_{ph(u)}$, are phenotype-associated (as well as dependent on the population size). In other words, the rates depend both on the clone size and on the functional effect of the accumulated mutations.
>
> We therefore define the process describing the number of cells sharing mutational profile u as

$$X_u(t-\sigma_u) \sim SDBD\,(a_{ph(u)}, b_{ph(u)}), \quad T \geq \sigma_u,$$
$$X_u(t)=0, \quad t<\sigma_u,$$
$$X_u(\sigma_u)=1$$

where SDBD stands for *Size-Dependent Birth and Death* process.

In order to better describe the model and the dependencies included in the parameters, we added the section “The cancer progression model – The phenotype as the model parameters manager”, lines 288-385. This section is a revised and summarized version of section S1.4 that has been deleted. It includes a detailed description of the quantities included in Eq. 4, which are actually essential for the understanding of the Results.

The phenotype as the model parameters manager At a high level, the stochastic process defined by the generator in Eq.(8) relies on three fundamental rates: those governing growth, $a_{ph(u)}(\tau,x)$ and $b_{ph(u)}(\tau,x)$, and the rate describing mutagenesis, $\mu_{ph(u)}(\tau,x)$. However, our framework establishes a deep structural link between these terms through the phenotype and the capabilities acquired by the associated clone.
In order to account for overall resource limitations and for different capabilities to control such resources, we rely on the competitive Lotka Volterra model for any number of species. A detailed analysis of this kind of models is presented in Section S1. In particular we decide to represent the global growth rate of the clone, which is given by $a_{ph(u)}(\tau,x) + \mu_{ph(u)}(\tau,x) - b_{ph(u)}(\tau,x)$, as described in Eq. (S1.3). As a result we have

$$a_{ph(u)}(\tau,x)+\mu_{ph(u)}(\tau,x)-b_{ph(u)}(\tau,x)=\lambda_{ph(u)}\left(1-\frac{x_u+\sum_{\substack{v\in\tau,\, v\neq u}}\alpha_{ph(u)ph(v)}x_v}{k_{ph(u)}}\right),$$

where $\lambda_{ph(u)}$, $\alpha_{ph(u)ph(v)}$, and $k_{ph(u)}$ are parameters depending solely on the phenotype of population u (and not on the population size) which represent respectively a global maximal growth rate λ, the carrying capacity k (as the maximum number of cells the system can sustain) and the interaction terms α's. The coefficients $\alpha_{ph(u)ph(v)}$ dictates the nature of the interplay between the clones with phenotypes ph(u) and ph(v).
To refine this system, we decompose the mutational rate $\mu_{ph(u)}(\tau,x)$ into two components: the expected number of mutations per cell division, $m_{ph(u)}$, and the frequency of divisions per unit of time. Notably, this frequency is exactly the birth rate $a_{ph(u)}(\tau,x)$, leading to the definition:

$$\mu_{ph(u)}(\tau,x) := m_{ph(u)} \cdot a_{ph(u)}(\tau,x).$$

Furthermore, we set the death rate $b_{ph(u)}$ to the constant value equal to 1, which is not only convenient, but also it gives a natural interpretation of the simulation’s time unit: if the death rate is equal to one, then the time unit is the expected life time of a cell of the tissue under analysis, see [35]. The system of equations governing our parameters becomes:

$$b_{ph(u)}(\tau,x) = 1$$
$$a_{ph(u)}(\tau,x) + \mu_{ph(u)}(\tau,x) - b_{ph(u)}(\tau,x) = \lambda_{ph(u)} \left(1 - \frac{x_u + \sum_{v \neq u} \alpha_{ph(u)ph(v)}\, x_v}{k_{ph(u)}} \right)$$
$$\mu_{ph(u)}(\tau,x) = m_{ph(u)} \cdot a_{ph(u)}(\tau,x)$$

By solving this system, we can express the birth and mutation rates explicitly:
label{eq:adjusted_rates}

$$b_{ph(u)}(\tau,x) = 1$$

$$a_{ph(u)}(\tau,x) = \frac{1}{m_{ph(u)} + 1} \left[\lambda_{ph(u)} \left(1 - \frac{x_u + \sum_{v \in \tau, v \neq u} \alpha_{ph(u)ph(v)} x_v}{k_{ph(u)}} \right) + 1 \right]$$

$$\mu_{ph(u)}(\tau,x) = \frac{m_{ph(u)}}{m_{ph(u)} + 1} \left[\lambda_{ph(u)} \left(1 - \frac{x_u + \sum_{v \in \tau, v \neq u} \alpha_{ph(u)ph(v)} x_v}{k_{ph(u)}} \right) + 1 \right]$$

This reduction leaves us with four actual parameters that drive the evolution of the tumor and that are independent from the state of the process: the proliferative advantage $\lambda_{ph(u)}$, the carrying capacity $k_{ph(u)}$, the interaction coefficients $\alpha_{ph(u)ph(v)}$, and the mutational parameter $m_{ph(u)}$. As previously underlined, these rules depend solely on the phenotype. However, individual functional events do not affect all parameters equally. We now detail how specific categories of functional effects map onto these four parameters.

Proliferative Advantage. A functional event of type 1-Deregulation of the proliferation program is characterized by a single parameter s_i, denoting the proliferative advantage conferred by that specific mutation. In accordance with the principle of clonal evolution, these effects are assumed to be summative as they accumulate. Consequently, by isolating the growth-related events from the phenotype,

$$F_{growth} := \{f_i \in F : F_i = 1\},$$

we define the total proliferative advantage as:

$$\lambda_{ph(u)} := \sum_{f_i \in F_{growth} \cap ph(u)} s_i.$$

Crucially, if a cell has not acquired any mutations altering cell cycle regulation, its growth rate remains null. This accurately reflects the natural state of healthy tissue, where cell birth and death are in a perfect, homeostatic balance.

Mutation Rate. While healthy cells have a balanced growth rate, they do not have a null mutation rate; DNA replication is inherently prone to occasional errors. We therefore define a baseline mutational parameter $m_{\beta ase}$. Empirical data suggest the somatic mutation rate in normal human cells is approximately $1 \cdot 10^{-9}$ to $5 \cdot 10^{-9}$ per base pair per division [50,51]. Functional events of type 2-Mutation burden augmentation serve to amplify this baseline. For each such event f_i in

$$F_{mut} := \{f_i \in F : F_i = 2\},$$

we define a mutational amplificator factor m_i. The cumulative mutational parameter for the phenotype is then:

$$m_{ph(u)} := \left[\prod_{f_i \in F_{mut} \cap ph(u)} m_i \right] \cdot m_{\beta ase} \cdot N_{\beta p},$$

where $N_{\beta p}$ represents the number of base pairs under analysis. This term is highly adaptable; it can represent the entire genome or be scaled to match the coverage of a specific sequencing panel when comparing synthetic data to clinical results.

Ecological Interaction. Because tumor clones share the same physical microenvironment, they naturally compete for resources, establishing a baseline interaction of $\alpha_{ph(u)ph(v)} = \alpha_{ph(v)ph(u)} = 1$. The parameter $\alpha_{ph(u)ph(v)}$ represents the ``ecological weight'' that population v exerts on the growth limitation of population u. Managing independent interaction parameters for every possible pair of populations is computationally and biologically impractical. Instead, we derive these values from the ``ecological power'' of a population's mutations. Each type 4-Resource control functional effect f_i in

$F_{res} := \{f_i \in F : F_i = 4\}$

is characterized by two values: a susceptibility index v_i and an offensive score η_i:

- v_i (susceptibility index) measures how strongly a population is affected by interactions with another: it can be interpreted as a relative weight to be applied to the elements of the other population to get the effect on the reference population. Hence, if positive but smaller than one, it indicates that the elements of the other population subtract resources shared with the reference population only partially, while if larger than one, it accounts for a substantial removal of resources. If negative, it describes a beneficial interaction in which the other population supplies resources necessary for the focal population.
- η_i (offensive score) quantifies the reference population's impact on the other: it can be interpreted as a relative weight to be applied to the elements of the reference population to get the effect on the other population. Hence, if positive but smaller than one, it indicates that the elements of the reference population subtract resources shared with the other population only partially, while if larger than one, it accounts for a substantial removal of resources. If negative, it describes a beneficial interaction in which the reference population supplies resources necessary for the other population.

From these indices, we derive the interaction coefficient α_{ph(u)ph(v)} as:

$$\alpha_{ph(u)ph(v)} := \sum_{f_i \in F_{res} \cap ph(u) \setminus ph(v)} (v_i - 1) + \sum_{f_i \in F_{res} \cap ph(v) \setminus ph(u)} (\eta_i - 1) + 1.$$

This additive formula treats 1 as the neutral element of competition. It balances the ``offensive score'' of mutations unique to population v against the ``susceptibility index'' of mutations unique to u. If X_u has no vulnerability alterations ($v_i = 1$), the interaction depends entirely on the offensive score of X_v, and vice versa, ensuring the model remains consistent with common-sense ecological constraints.

Carrying capacity. Finally, the carrying capacity k_{ph(u)} is governed by functional events of type 3-Limit evasion. These mutations allow a clone to expand beyond the initial constraints of the environment, whether by physically finding more space, gathering resources more aggressively, or utilizing them more efficiently. For each such event f_i in

$F_{lim} := \{f_i \in F : F_i = 3\}$,

we define a parameter k_i representing the additional space provided. Starting from a baseline capacity $k_{\beta ase}$ for wild-type clones, the carrying capacity is:

$$k_{ph(u)} := k_{\beta ase} + \sum_{f_i \in F_{lim} \cap ph(u)} k_i.$$

It is important to distinguish between type 4-Resource control and type 3-Limit evasion functional effects. While both help a tumor overcome growth limitations related to space constraints and resources exhaustion, they operate through different mechanisms. The type 4 functional effects contribute in regulating how existing resources are internally allocated among clones through competition or cooperation. In contrast, type 3 functional events induce an expansion mechanism, allowing specific clones to access previously unavailable nutrients or niches, thereby providing an exclusive advantage that alters the tumor's global resource dynamics.

4. Line 383: define VCF at first use.

We defined VCF at its first use, line 472.

5. I think it would be helpful to expand the figure captions to explain the cards which indicate the type of functional advantage considered, to ensure there is no misunderstandings. I'd like to further understand the difference between Fig 3 top right and Fig 3 top left – I believe it's a functional advantage (multiplier??) for proliferation for each subsequent mutation – perhaps capped at N=2 total mutations? If so, the result that similar patterns as neutral evolution can be observed in non-neutral is also shown in a similar model in previous research (DOI: 10.1038/s41467-021-22123-1), related to lines 433-448 in the manuscript. The mechanism in that research is spatial competition, which I don't think is considered in Figure 3. Can the authors clarify this, and why their model isn't similar to non-neutral non-spatial patterns of diversity?

We defined the cards at their first use in the caption of Fig. 6, as we deleted the cards in the revised Fig. 5 (old Fig. 3).

> On the **central column**, the maximum likelihood estimates parameters of the model are represented as **cards** with a symbol corresponding to the type of the primary mechanism associated to the functional event and the values of the parameters. For the sake of readability, the cards are grouped by primary mechanism and parameters values. The name of the primary mechanism is reported on the left and the frequency of association of each card to a newly acquired mutation is reported at the base of each group of cards. The full set of cards is a visualization of the functional event list F, see Eq. (1). The light gray shaded card is the phenotype of the initiating cell.

The reviewer's interpretation of the Muller plots reported in the revised Fig. 5 top-left and bottom-left panels is correct. When the model includes a carrying capacity term, functional events providing an increase in the birth rate of the cells (type 1-Deregulation of the proliferation program) give rise to clones able to reach larger sizes only if they appear very early in the tumor evolution, the expansive phase before the carrying capacity is attained. Indeed, as carrying capacity is reached and no resources are available, an increase in the birth rate becomes ineffective as it is capped by scarcity of shared resources. Hence, the Muller plot will show a clone with detectable size (gained in the expansive phase) and no subsequent modifications of the relative sizes (in the stable phase), see Fig. 5 top-left panel. A very similar shape can be obtained even when only passenger mutations are acquired in the expansive phase of the tumor evolution, see Fig. 5 bottom-left panel. However, in the latter case, this is a rare event that can be observed when a random fluctuation of clone sizes changes by chance the relative abundances. After this random (and unlikely) event, the altered sizes are conserved and a clone of larger size can be detected even if no alterations of the proliferation program were experienced. We improved the description of Fig. 5, lines 559-607.

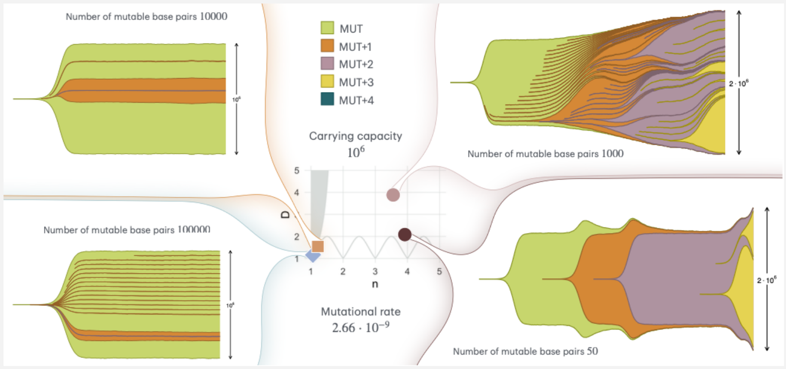

Fig.5 Examples of clonal architectures observed in the experiments. Four dots in the (n,D) plane (single simulated tumors) are illustrated through the Muller plots, coloured according to the number of mutational events associated with the clone. MUT represents the first oncogenic mutation originating the tumor. The initiating cell has a single mutation with associated functional effect of type 1-Deregulation of the proliferation program defined by the parameter sbase = 0.1, see Eq. (11). The **bottom-left panel** reports an experiment in which only type 0-Null effect (passenger) functional effects can be associated to mutations. The **top-left panel** reports an experiment in which only single type 1-Deregulation of the proliferation program functional effects can be associated to mutations (with parameter s= 0.1). The **top-right panel** reports an experiment in which six type 4-Resource control functional effects maximum can be associated to mutations, (with identical parameters (v = 0.7,η= 1), see Eq. (13)). The **lower-right panel** reports an analogous experiment with six type 4-Resource control functional effects maximum, but with parameters (v = 0.1,η= 1). Other parameters are: baseline mutational rate $m_{base} = 2.66 \cdot 10^{-9}$ per base pair, number of mutable base pairs Nbp = 10000 (upper-left panel), Nbp = 100000 (lower-left panel), Nbp = 50 (lower-right panel), Nbp = 1000 (upper-right panel), carrying capacity $K = 10^{6}$ cells.

The zoomed examples represented in Fig.5 illustrate the microscopic origin of these macroscopic patterns. Under pure passenger accumulation (type 0-Null effect functional events), most subclones remain extremely small, with only rare events producing detectable expansions, an example of these uncommon occurrence is visible in Fig.5 bottom-left panel. When such expansions occur, they are not driven by any intrinsic advantage of the clone, but by a purely stochastic fluctuation: it is only by chance that lineages in the surrounding population go extinct, allowing the neutral clone to drift to an unexpectedly large prevalence that is then preserved once carrying capacity is reached. This explains why these events are observed only for mutations that arise very early, during the expansive phase of the tumor evolution. By contrast, proliferative deregulation mutations (type 1-Deregulation of the proliferation program) exploit a genuine selective advantage during this same phase, which makes them consistently more likely to expand as visible in Fig. 5 top-left panel. Once the carrying capacity is reached, however, this advantage becomes ineffective: clonal sizes stabilize, and newly acquired proliferative mutations no longer produce significant expansions. As a result, both neutral-like and proliferative regimes converge to the same thin, sweep-like structures, explaining why they collapse onto the same low-n region in Fig. 4. In this sense, the framework also recovers signatures typically associated with *neutral evolution*: in [24], the authors already showed that, under neutrality, passenger mutations yield a VAF distribution heavily skewed toward very low frequencies, exactly as we observe in our simulations; however, their conclusion

that such patterns imply predominantly neutral evolution has been widely criticized [55-57]. Indeed in our framework low-frequency variants arise not only from passenger mutations (type 0), but also from proliferative deregulation mutations (type 1) acquired in the stable phase, where their selective advantage cannot be expressed.

The key result of the systematic exploration of the tumor evolution under our model is that this degeneracy, caused by the resources exhaustion when the system reaches the carrying capacity, is broken only by ecological interaction terms. In particular, the susceptibility index v, associated to type 4-Resource control functional effects, acts as the main control knob of the system. Recall that large positive values of the parameter indicate that the population is strongly impaired by competitors, whereas positive values below one indicate a weaker harming effect due to competition. Equivalently, large positive values of the parameter indicate that the population has weak resistance to competitors, whereas positive values below one indicate a stronger response to competition. The sensitivity of the model to this parameter is discussed in details in Section S3 Parameter exploration, yet it is worth noting that varying this parameter allows for spanning from complete clonal sweeps to fully branched architectures of evolution. When the tumor accumulate, at low rate, mutations associated to type 4-Resource control functional effects with small positive values of v, the new populations have strong resistance to competition and, given the low rate of mutation, they have the time to increase their sizes by using a larger amount of the limited resources letting the system collapse into near-perfect selective sweeps, see Fig.5 bottom-right panel. Under these conditions, only a single lineage at a time can effectively persist, reproducing dynamics consistent with *linear evolution*. Conversely, for milder resistance (larger susceptibility parameters), multiple lineages can coexist and expand simultaneously, producing architectures such as that in Fig.5 top-right panel, and giving rise to *branching evolution*. These two regimes emerge from continuous parameter variations within the same mechanism, rather than from fundamentally distinct model assumptions.

We thank the reviewer for suggesting the paper *(DOI: 10.1038/s41467-021-22123-1).* We included the paper in the References. We added an explicit comment on the similarities/dissimilarities to the model presented by West et al. in the Discussion, lines 775-819. The reviewer is correct: we do not have a spatial component in the model, our model is for a well-mixed population of cells, while West et al. propose a model for spatially segregated, with partial mixing, population of cells that are not affected by resources limitations.

A major limitation of many tumor evolution models lies in their difficulty in generating heterogeneous cell populations that provide a realistic representation of intratumor heterogeneity (ITH). Two main modelling strategies may be considered to address this issue. The first consists in introducing a spatial structure for the environment in which cells proliferate, so as to promote the segregation of the tumor mass into distinct subpopulations, see for example [39-41]. Under assumptions ensuring unbounded growth, with no saturation of either space or available resources, each spatially segregated niche gives rise to a distinct evolutionary trajectory, thereby leading to increasing genetic diversity. The resulting tumor exhibits a controllable degree of ITH, determined by the extent of segregation among the different subpopulations. A second approach, on which we

focus here inspired by [77], consists in considering a well-mixed population of cells with growth limitations and capable of exhibiting different mechanisms of resources exploitation. Both the growth limitation and the different resources exploitations are necessary to show modelled tumors with genetic heterogeneity. Indeed, a growth constraint alone tends to reduce genetic variability, since saturation of the limited resources yields a system in which the relative frequencies of the subclones present at the time the maximum population size is reached can no longer change, thereby fixing the genetic composition at all subsequent times. The model should therefore explicitly incorporate mechanisms of differential exploitation of the limited resources (the type 4-Resource control functional effect), under which subpopulations able to recruit a larger share of the scarce resources can achieve greater growth than others. A similar attempt has been proposed in [42], where a double growth-constraint mechanism acting both on the total population size (global confinement) and on individual subclones (local confinement) is used to mimic competition for shared resources, thereby generating genetically heterogeneous tumors. However, we believe that the ability to evade growth constraints and the ability to differentially dispose of the shared resources have to be mapped into different parts of the model. Moreover we think that those mechanisms should be clone-specific instead of shared by all the population. Indeed, those capabilities can be biologically thought as emerging from the specific set of genetic alterations that have been accumulated by the single clone.
The two approaches to modelling here discussed, the spatially organized population and the well-mixed but subject to competition for limited resources population, lead to genetically heterogeneous tumors though diverging conceptually: we argue that it is not the geometry of space itself that governs tumor evolution, but rather the control and distribution of shared resources. In our framework, competition arises from differential access to these resources and can be modulated by new driver mutations, thus the creation of new spatial compartments is not required to explain clonal diversity. Still, these spatially explicit models remain valuable, and for tumor types where physical architecture plays a dominant role, their principles could be seamlessly integrated into our simulator, further demonstrating its flexibility and generality. It would also be interesting to explore models that are neither fully well-mixed nor fully spatially segregated, but instead display an intermediate degree of local-neighborhood interactions. In this setting, it would be important to determine which results are found to be robust.

6. Again, in figure 4 it's not really clear to me what the cards mean. What are the small numbers (0.01, 0.01, …) and the large numbers (0, 6, 5, 4, …) indicating? Are these simulations' parameters inferred from data? How is this procedure done?

We agree with the reviewer, the cards were not described adequately. The description of the cards, including the small numbers and large numbers meanings is now included in the caption of Fig. 6. Please note that now the figure of this comment is numbered as Fig. 6.

On the **central column**, the maximum likelihood estimates parameters of the model are represented as **cards** with a symbol corresponding to the type of the

primary mechanism associated to the functional event and the values of the parameters. For the sake of readability, the cards are grouped by primary mechanism and parameters values. The name of the primary mechanism is reported on the left and the frequency of association of each card to a newly acquired mutation is reported at the base of each group of cards. The full set of cards is a visualization of the functional event list F, see Eq. (1). The light gray shaded card is the phenotype of the initiating cell.

The parameters of the plotted simulations have been inferred from the real data (black crosses in the left column plots). The procedure for the parameters inference is described in the revised part of the main text, lines 658-668, and in the accompanying Fig. S3.13. We revised the description of the inference procedure in order to make it more clear.

Different configurations of primary mechanisms and of their parameters have been tried, running 100 simulations for each chosen configuration. Then, the (n,D) metrics have been calculated and each resulting group of 100 points (corresponding to the 100 simulations) derived from the same parameter set θ has been used to construct a 2-dimensional distribution via kernel estimation f_{θ}(n,D). The estimated density f_{θ}(n,D) is used to compute the likelihood function at the real experimental sample points (the black crosses on the (n,D) plane), which is equal to $L(\theta)=\prod_{i\in 1\ldots n} f_{\theta}(n_i,D_i)$. A visual representation of the procedure is depicted in Fig.S3.13. The likelihood value is a good estimation of the goodness of fit of the parameter set, hence the parameters realizing the maximum are chosen and represented in Fig.6.

7. Is figure 5 a result or a schematic? The axes are not labelled, so I'm not clear what's being shown here. I also don't know what the rows/columns of the table indicate, or what parameters were used here.

The reviewer is right, the old Fig. 5 is a description of the multiregional sampling procedure from synthetic tumors. Hence, it should be placed in the new section "Methods and Materials – Methods for downstream analysis of simulated tumors" where we collected all the methods developed for the downstream analysis of simulated tumors.

To improve the readability of the old Fig. 5, now numbered as Fig. 3, we labelled the axes, revised the caption and the text that describes the multiregional sequencing procedure, lines 477-490.

The revised caption:

Procedure of multiregional sequencing used for the classification of mutations based on the spatial pattern of occurrence. For each synthetic tumor, we extract the synthetic VCF, over 10 non-overlapping regions. Single mutations are classified as *Public*, *Private Unique*, *Public Regional* and *Private Regional* depending on the frequency of occurrence. The two-way contingency table of the joint distribution of the type of functional event associated to the mutation and the Public/Private spatial pattern is derived. The contingency tables from the single tumors are summed up to a single contingency table, which shows how different types of functional events (functional capabilities) are associated to distinct spatial patterns. The overall contingency table can be plotted as grouped barplot as well.

The revised description of the multiregional sequencing procedure:

> We developed a multiregional sequencing of the synthetic tumors as well. For each synthetic tumor, we repeated (10 times) the procedure described in Fig. 2 to extract a synthetic VCF, over non-overlapping regions made of 10^4 cells. Relying on the individual mutation IDs, mutations detected in all (or all but one) sequenced regions were classified as *Public*; mutations detected in a single sequenced region as *Private Unique*; and mutations shared by some but not all sequenced regions as *Regional*, further subdivided into *Public Regional* (present in $4-8$ regions) and *Private Regional* (present in $2-3$ regions). At this point, from each synthetic tumor, we have obtained a list of point mutations with associated functional event and classification into Public/Private spatial pattern. We group the point mutations by functional event and we get the two-way contingency table of the joint distribution of the functional event and the Public/Private spatial pattern. The contingency tables from the single tumors were finally summed up to a single contingency table, which shows how different functional events (functional capabilities) are associated to distinct spatial patterns.

—------

Reviewer #2:

The manuscript presents a new model of development of intra-tumor-heterogeneity. The work follows in the trajectory of extending stochastic process (population-based) models with phenotype-derived fitness properties.

As there is already a plethora of existing models, the main challenge for a model is to demonstrate this model is useful, (i) either by extending our understanding of the modelled system on a human-understandable level, or (ii) by being able to create testable predictions about the system. In both cases I feel the answer is potentially yes, with caveats.

We thank the referee for the careful reading of the manuscript and for the constructive comments and suggestions. We are submitting a revised version of the manuscript following the suggested recommendations. At a general level, we can say that we appreciate the comments and, in response, we substantially revised the section "Results – Exploration of Evolutionary Paradigms" by introducing a new set of experiments aimed at clarifying which mechanisms can generate synthetic tumors that reproduce key features of real tumors, such as heterogeneity and clonal diversification. Our proposed model points to the type 4-Resource control and type 3-Limit evasion functional events as the primary candidate mechanism for the emergence of ITH. Moreover, we believe that there is biological evidence that tumor evolution happens in a system that is able to overcome strong limitations both in space and shared resources. Hence we propose here a model that studies the mechanisms classically proposed in the literature, such as the "fitness increase" of the cancerous cells, in the context of a system with limited resources (limited carrying capacity). We try to explain that this condition makes the birth rates increase ineffective, as the limitation of space and resources cap the growth of the population which should acquire other capabilities such as the type 4 and type 3 described in our model and very well established in the literature as hallmarks of cancer. In order to better develop those concepts, we included the references suggested by the reviewer (see below) and revised the "Discussion" section to provide a more detailed presentation of our findings.

Here it follows the list of responses to the detailed comments. The reviewers' original comments are reproduced in *yellow italics*, the authors' responses are presented in plain black text, and excerpts from the manuscript are shown with gray background. All the reported line numbers, figure numberings and equation numberings are referred to the clean copy of the revised manuscript.

Major comments:
In terms of the model, the authors compare the results to [38, 39], both of which are simpler models, focused on demonstrating the origin of ITH from spatial structure of the environment. The model of Volpatto et. al. uses a more complex double-stochastic process with explicitly modelled mechanisms for 5 functional events. The choice of modelling framework and modelled phenotypes results in a, comparatively speaking, complex mathematical model. Explicit modelling of hallmarks in a stochastic process is by itself not a novelty and is present e.g. in tugHall model (Nagornov 2020). I think this work should be at least shortly compared.

We included the suggested reference and we added a comment on the tugHall model in the section "Discussion", lines 765-774.

In [76], the authors present a recent attempt to incorporate more complex aspects of cancer progression into the model. Their work is aligned with our perspective in adopting the hallmarks framework as a guiding principle, although their analysis is restricted to apoptosis, invasion (metastatic transformation), immortalization, and cell division. Moreover, their model is not fully stochastic, since cell growth is described only through mean trajectories, while stochasticity is confined to what the authors term the “trial” sequence. Furthermore, the model does not account for interactions among clones, which ,in our approach, constitute the key mechanism for describing hallmarks related to metabolic reprogramming and to the induction of, and access to, tumor-associated neovasculature.

Utility of the model therefore has to be demonstrated, since the model is quite complex and not that novel. The authors argue that spatial segregation is not needed to reconstruct various patterns of heterogeneity. This may be true, but this model utilizes carrying capacity and limit evasion, which principally falls into the same category. Generally, for heterogeneity to arise, the systems must have a growth limitation that is non-linearly dependent on the population size. The effects of population limitation on diversity are discussed in e.g. “Hu, 2017, A population genetics perspective on the determinants of intra-tumor heterogeneity”. If the authors feel that carrying capacity + evasion is significantly different than population limits imposed by others it should be discussed, but in my mind the papers the authors compare to are all examples of non-linearly size-dependent branching process and it’s not a distinguishing factor of this model. That does not present a problem, but it seems that authors felt their work differs significantly in this regard (79-81, 630-637).

We find this comment very insightful. We read the suggested reference together with “Normal tissue architecture determines the evolutionary course of cancer, West, 2021” (suggested below) and [39]. We develop our understanding of the differences between models with spatial segregation and models for well-mixed populations with carrying capacity and competition in the section “Discussion”, lines 775-819.

A major limitation of many tumor evolution models lies in their difficulty in generating heterogeneous cell populations that provide a realistic representation of intratumor heterogeneity (ITH). Two main modelling strategies may be considered to address this issue. The first consists in introducing a spatial structure for the environment in which cells proliferate, so as to promote the segregation of the tumor mass into distinct subpopulations, see for example [39-41]. Under assumptions ensuring unbounded growth, with no saturation of either space or available resources, each spatially segregated niche gives rise to a distinct evolutionary trajectory, thereby leading to increasing genetic diversity. The resulting tumor exhibits a controllable degree of ITH, determined by the extent of segregation among the different subpopulations. A second approach, on which we focus here inspired by [77], consists in considering a well-mixed population of cells with growth limitations and capable of exhibiting different mechanisms of resources exploitation. Both the growth limitation and the different resources exploitations are necessary to show modelled tumors with genetic heterogeneity. Indeed, a growth constraint alone tends to reduce genetic variability, since saturation of the limited resources yields a system in which the relative frequencies of the subclones present at the time the maximum population size is reached can no longer change, thereby fixing the genetic composition at all

subsequent times. The model should therefore explicitly incorporate mechanisms of differential exploitation of the limited resources (the type 4-Resource control functional effect), under which subpopulations able to recruit a larger share of the scarce resources can achieve greater growth than others. A similar attempt has been proposed in [42], where a double growth-constraint mechanism acting both on the total population size (global confinement) and on individual subclones (local confinement) is used to mimic competition for shared resources, thereby generating genetically heterogeneous tumors. However, we believe that the ability to evade growth constraints and the ability to differentially dispose of the shared resources have to be mapped into different parts of the model. Moreover we think that those mechanisms should be clone-specific instead of shared by all the population. Indeed, those capabilities can be biologically thought as emerging from the specific set of genetic alterations that have been accumulated by the single clone.
The two approaches to modelling here discussed, the spatially organized population and the well-mixed but subject to competition for limited resources population, lead to genetically heterogeneous tumors though diverging conceptually: we argue that it is not the geometry of space itself that governs tumor evolution, but rather the control and distribution of shared resources. In our framework, competition arises from differential access to these resources and can be modulated by new driver mutations, thus the creation of new spatial compartments is not required to explain clonal diversity. Still, these spatially explicit models remain valuable, and for tumor types where physical architecture plays a dominant role, their principles could be seamlessly integrated into our simulator, further demonstrating its flexibility and generality. It would also be interesting to explore models that are neither fully well-mixed nor fully spatially segregated, but instead display an intermediate degree of local-neighborhood interactions. In this setting, it would be important to determine which results are found to be robust.

I would also argue that an increase in a mutational rate is not terribly useful to model in general, since it mainly increases the number of small clones in a later portion of simulation, which are usually ignored in summary statistics anyway. As it's not listed in the phenotype of any fit in Fig.4., I guess the optimization process yields the same conclusion. Similarly, neutral mutations are modelled in other frameworks, but as they do not affect the behaviour, they are not part of the fit. This leaves only novelty in the Resource Control phenotype, which affects the context dependent competition between clones.The structure of the article makes it difficult to judge the benefit of the phenotype model, exacerbated by the fact that the mathematical formulation of the individual hallmarks is actually not in the main text. This I find to be an issue, since the result (Fig. 3, 4) fit the individual hallmarks and their numerical values, but from the main text e.g. the difference between Resource control (0.7, 1) and (0.1, 1) is not clear.

The reviewer is right about the mutational rate and the neutral mutations. We think that the comment on the structure of the paper is correct, as well. In order to better describe the mathematical model and the dependencies included in the parameters, we added the section “The cancer progression model – The phenotype as the model parameters manager”, lines 288-385. This section is a revised and summarized version of the old section S1.4 that has been deleted. It includes a detailed description of the quantities

included in Eq. 4 together with the involved parameters, which is actually essential for the understanding of the Results.

The phenotype as the model parameters manager At a high level, the stochastic process defined by the generator in Eq.(8) relies on three fundamental rates: those governing growth, $a_{ph(u)}(\tau,x)$ and $b_{ph(u)}(\tau,x)$, and the rate describing mutagenesis, $\mu_{ph(u)}(\tau,x)$. However, our framework establishes a deep structural link between these terms through the phenotype and the capabilities acquired by the associated clone.
In order to account for overall resource limitations and for different capabilities to control such resources, we rely on the competitive Lotka Volterra model for any number of species. A detailed analysis of this kind of models is presented in Section S1. In particular we decide to represent the global growth rate of the clone, which is given by $a_{ph(u)}(\tau,x) + \mu_{ph(u)}(\tau,x) - b_{ph(u)}(\tau,x)$, as described in Eq. (S1.3). As a result we have

$$a_{ph(u)}(\tau,x)+\mu_{ph(u)}(\tau,x)-b_{ph(u)}(\tau,x)=\lambda_{ph(u)}\left(1-\frac{x_u+\sum_{\substack{v\in\tau,\ v\neq u}}\alpha_{ph(u)ph(v)}x_v}{k_{ph(u)}}\right),$$

where $\lambda_{ph(u)}$, $\alpha_{ph(u)ph(v)}$, and $k_{ph(u)}$ are parameters depending solely on the phenotype of population u (and not on the population size) which represent respectively a global maximal growth rate λ, the carrying capacity k (as the maximum number of cells the system can sustain) and the interaction terms α's. The coefficients $\alpha_{ph(u)ph(v)}$ dictates the nature of the interplay between the clones with phenotypes ph(u) and ph(v).
To refine this system, we decompose the mutational rate $\mu_{ph(u)}(\tau,x)$ into two components: the expected number of mutations per cell division, $m_{ph(u)}$, and the frequency of divisions per unit of time. Notably, this frequency is exactly the birth rate $a_{ph(u)}(\tau,x)$, leading to the definition:

$$\mu_{ph(u)}(\tau,x) := m_{ph(u)} \cdot a_{ph(u)}(\tau,x).$$

Furthermore, we set the death rate $b_{ph(u)}$ to the constant value equal to 1, which is not only convenient, but also it gives a natural interpretation of the simulation's time unit: if the death rate is equal to one, then the time unit is the expected life time of a cell of the tissue under analysis, see [35]. The system of equations governing our parameters becomes:

$$b_{ph(u)}(\tau,x) = 1$$
$$a_{ph(u)}(\tau,x) + \mu_{ph(u)}(\tau,x) - b_{ph(u)}(\tau,x) = \lambda_{ph(u)} \left(1 - \frac{x_u + \sum_{v \neq u} \alpha_{ph(u)ph(v)} x_v}{k_{ph(u)}} \right)$$
$$\mu_{ph(u)}(\tau,x) = m_{ph(u)} \cdot a_{ph(u)}(\tau,x)$$

By solving this system, we can express the birth and mutation rates explicitly:
label{eq:adjusted_rates}

$$b_{ph(u)}(\tau,x) = 1$$
$$a_{ph(u)}(\tau,x) = 1/(m_{ph}(u) + 1) \left[\lambda_{ph(u)} \left(1 - \frac{x_u + \sum_{v \in \tau, v \neq u} \alpha_{ph(u)ph(v)} x_v}{k_{ph(u)}} \right) + 1 \right]$$
$$\mu_{ph(u)}(\tau,x) = \frac{m_{ph(u)}}{m_{ph(u)} + 1} \left[\lambda_{ph(u)} \left(1 - \frac{x_u + \sum_{v \in \tau, v \neq u} \alpha_{ph(u)ph(v)} x_v}{k_{ph(u)}} \right) + 1 \right]$$

This reduction leaves us with four actual parameters that drive the evolution of the tumor and that are independent from the state of the process: the proliferative advantage $\lambda_{ph(u)}$, the carrying capacity $k_{ph(u)}$, the interaction coefficients $\alpha_{ph(u)ph(v)}$, and

the mutational parameter $m_{ph(u)}$. As previously underlined, these rules depend solely on the phenotype. However, individual functional events do not affect all parameters equally. We now detail how specific categories of functional effects map onto these four parameters.
Proliferative Advantage. A functional event of type 1-Deregulation of the proliferation program is characterized by a single parameter s_i, denoting the proliferative advantage conferred by that specific mutation. In accordance with the principle of clonal evolution, these effects are assumed to be summative as they accumulate. Consequently, by isolating the growth-related events from the phenotype,

$$F_{growth} := \{f_i \in F : F_i = 1\},$$

we define the total proliferative advantage as:

$$\lambda_{ph(u)} := \sum_{f_i \in F_{growth} \cap ph(u)} s_i.$$

Crucially, if a cell has not acquired any mutations altering cell cycle regulation, its growth rate remains null. This accurately reflects the natural state of healthy tissue, where cell birth and death are in a perfect, homeostatic balance.
Mutation Rate. While healthy cells have a balanced growth rate, they do not have a null mutation rate; DNA replication is inherently prone to occasional errors. We therefore define a baseline mutational parameter $m_{\beta ase}$. Empirical data suggest the somatic mutation rate in normal human cells is approximately $1 \cdot 10^{-9}$ to $5 \cdot 10^{-9}$ per base pair per division [50,51]. Functional events of type 2-Mutation burden augmentation serve to amplify this baseline. For each such event f_i in

$$F_{mut} := \{f_i \in F : F_i = 2\},$$

we define a mutational amplificator factor m_i. The cumulative mutational parameter for the phenotype is then:

$$m_{ph(u)} := \left[\prod_{f_i \in F_{mut} \cap ph(u)} m_i \right] \cdot m_{\beta ase} \cdot N_{\beta p},$$

where $N_{\beta p}$ represents the number of base pairs under analysis. This term is highly adaptable; it can represent the entire genome or be scaled to match the coverage of a specific sequencing panel when comparing synthetic data to clinical results.
Ecological Interaction. Because tumor clones share the same physical microenvironment, they naturally compete for resources, establishing a baseline interaction of $\alpha_{ph(u)ph(v)} = \alpha_{ph(v)ph(u)} = 1$. The parameter $\alpha_{ph(u)ph(v)}$ represents the ``ecological weight'' that population v exerts on the growth limitation of population u. Managing independent interaction parameters for every possible pair of populations is computationally and biologically impractical. Instead, we derive these values from the ``ecological power'' of a population's mutations. Each type 4-Resource control functional effect f_i in

$$F_{res} := \{f_i \in F : F_i = 4\}$$

is characterized by two values: a susceptibility index v_i and an offensive score η_i:

- v_i (susceptibility index) measures how strongly a population is affected by interactions with another: it can be interpreted as a relative weight to be applied to the elements of the other population to get the effect on the reference population. Hence, if positive but smaller than one, it indicates that the elements of the other population subtract resources shared with the reference population only partially, while if larger than one, it accounts for a substantial removal of resources. If negative, it describes a beneficial interaction in which the other population supplies resources necessary for the focal population.

- η_i (offensive score) quantifies the reference population's impact on the other: it can be interpreted as a relative weight to be applied to the elements of the reference population to get the effect on the other population. Hence, if positive but smaller than one, it indicates that the elements of the reference population subtract resources shared with the other population only partially, while if larger than one, it accounts for a substantial removal of resources. If negative, it describes a beneficial interaction in which the reference population supplies resources necessary for the other population.

From these indices, we derive the interaction coefficient $\alpha_{ph(u)ph(v)}$ as:

$$\alpha_{ph(u)ph(v)} := \sum_{f_i \in F_{res} \cap ph(u) \setminus ph(v)} (v_i - 1) + \sum_{f_i \in F_{res} \cap ph(v) \setminus ph(u)} (\eta_i - 1) + 1.$$

This additive formula treats 1 as the neutral element of competition. It balances the ``offensive score'' of mutations unique to population v against the ``susceptibility index'' of mutations unique to u. If X_u has no vulnerability alterations ($v_i = 1$), the interaction depends entirely on the offensive score of X_v, and vice versa, ensuring the model remains consistent with common-sense ecological constraints.

Carrying capacity. Finally, the carrying capacity $k_{ph(u)}$ is governed by functional events of type 3-Limit evasion. These mutations allow a clone to expand beyond the initial constraints of the environment, whether by physically finding more space, gathering resources more aggressively, or utilizing them more efficiently. For each such event f_i in

$$F_{lim} := \{f_i \in F : F_i = 3\},$$

we define a parameter k_i representing the additional space provided. Starting from a baseline capacity $k_{\beta ase}$ for wild-type clones, the carrying capacity is:

$$k_{ph(u)} := k_{\beta ase} + \sum_{f_i \in F_{lim} \cap ph(u)} k_i.$$

It is important to distinguish between type 4-Resource control and type 3-Limit evasion functional effects. While both help a tumor overcome growth limitations related to space constraints and resources exhaustion, they operate through different mechanisms. The type 4 functional effects contribute in regulating how existing resources are internally allocated among clones through competition or cooperation. In contrast, type 3 functional events induce an expansion mechanism, allowing specific clones to access previously unavailable nutrients or niches, thereby providing an exclusive advantage that alters the tumor's global resource dynamics.

I find the resource control the most interesting part of the. Models like 38 or “Normal tissue architecture determines the evolutionary course of cancer, West, 2021” model a similar principle in the architecture of the tissue, while 39 uses abstract “local confinement”, however these are always symmetric between cell populations, whereas in this work a true heterogeneity arises. In general the effects of this per-clone specificity should be explained in more details. In summary of the model, I found it difficult to gain understanding of the text, which can be improved by restructuring. As far as I can tell, the model is a more complex version of 38, 39 (more free parameters), therefore it is to be expected that the same dataset can be fitted. It should be shown that the inclusion of the Resource Control can add a mechanistic understanding of the system. An ablation study where the hallmarks are removed or limited (e.g. shared RC values for all clones) could demonstrate it.

We designed a new set of experiments that have been included in the revision of the section “Results – Exploration of evolutionary paradigms” (lines 508-628) and the revision of the supplementary section “S3 Parameter Exploration” (lines 1571-1611). Those new experiments follow the reviewer’s suggestion of detailing the contribution of each type of functional effects separately and to improve the mechanistic understanding of the system. The reviewer’s suggestion has been leading the revision of the explanation of the other results included in section “Results – Exploration of evolutionary paradigms”. See the text, lines 508-628:

A systematic exploration of the model’s parameter space has first been conducted by isolating one type of functional event at a time, allowing the role of each functional mechanism to be disentangled across a broad range of configurations, see Section S3 Parameter exploration. That analysis revealed few robust findings: 1. a clear distinction between the behavior observed during the tumor’s initial expansive phase, when resources and space are still available, and the later stable phase, when those resources and space have been depleted; 2. the possibility that passenger mutations (type 0-Null effect functional events) give rise to clones that reach detectable sizes when they arise early in the expansive phase; 3. the fact that proliferative deregulation (type 1-Deregulation of the proliferation program functional events) give rise to non-neutral dynamics only during this early phase, showing larger survival probability and expansion potential. However, if the type 1 functional event is acquired later in the stable phase, where resources and space are no more available, the clone is not able to increase its size and become detectable. Taken separately, these observations do not yet establish to which extent each different mechanism is responsible for the emergence of sustained intratumor heterogeneity.

To address this, we turn to the integrated experiment summarized in Fig.4, which condenses many simulated tumors into a unified (n,D) representation. In this setting, each simulated tumor can accumulate mutations that are associated to six functional events maximum, all of the same type and with identical parameters. This design enables a controlled, differential comparison between functional mechanisms while still accounting for cumulative effects of repeated alterations of the same type. Each point in the figure therefore represents the outcome of a full stochastic trajectory under a specific mechanism and parameter choice. Simulations are stopped either upon reaching the maximum size M or the maximum time equal to 5 years. Three different values of M have been explored. A value smaller than the carrying capacity K, namely M=1/10K, which corresponds to an early stopping during the *expansive* phase of the tumor. Two values larger than the carrying capacity, M=2K and M=10K, corresponding to a stopping during an *early stable* phase of the tumor and a *late stable* phase of the tumor, respectively.

A first, clear separation emerges between the behaviors exhibited at the expansive phase and at the two stable phases. In the expansive regime, only the accumulation of mutations associated to type 1-Deregulation of the proliferation program is able to drive any appreciable departure from the baseline point (n=1, D=1). As predicted by [41], if the selective advantage s is sufficiently large, the evolution follows sequential selective sweeps and (dark) orange squares align on the light gray curves calculated in [41]. Diversity stays low and the number of

dominant clones never exceeds n ≈ 2, even when the system is observed across all replicates. In this regime, the framework naturally recovers *punctuated*-like dynamics: early proliferative events generate rapid expansions, after which the system quickly saturates and no further diversification occurs. This picture changes radically in the stable regime. Once resource saturation and ecological constraints become relevant, proliferative deregulation (type 1) loses explanatory power: its trajectories remain confined to the same low-diversity sweep structure observed in the early phase. In contrast, mechanisms based on resource control (type 4) and limit evasion (type 3) become the dominant drivers of evolution. They progressively occupy the entire (n,D) plane, generating both high-diversity configurations and complex branching patterns. The transition is not gradual in terms of qualitative behaviour: it is a regime shift in which the relevant explanatory mechanisms are different. Passenger-only configurations (type 0), by contrast, remain concentrated at the baseline point (n=1, D=1) across all phases, reflecting the fact that the accumulation of neutral mutations alone does not generate dominant clones at the population scale.

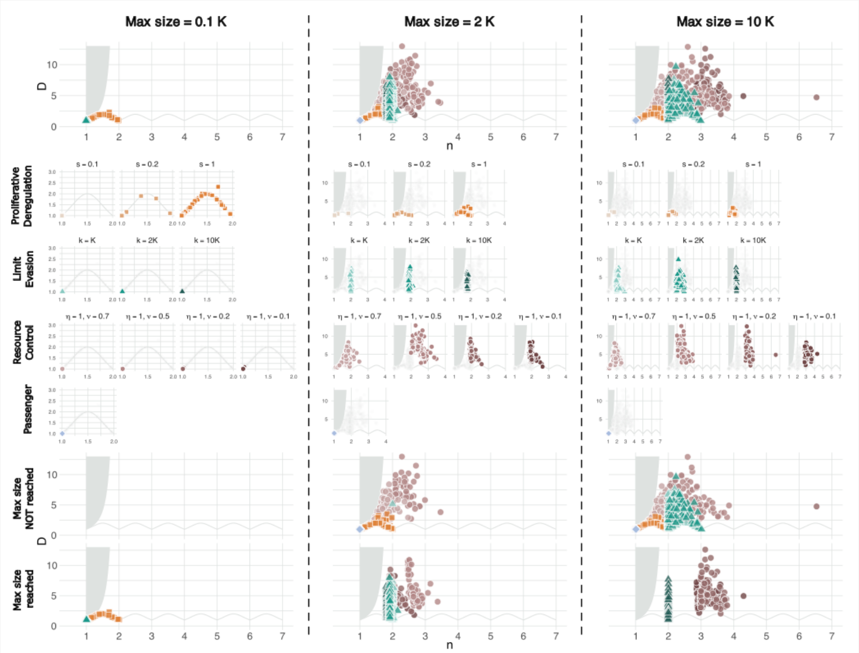

Fig 4. Simulated tumors on the (n,D) plane. Each point represents a single independent simulation run; the shape indicates the type of primary mechanism included, and the colour denotes the specific parameter configuration. Simulations start from a single cell already carrying a mutation associated to type 1-Deregulation of the proliferation program primary mechanism with parameter $s_{\beta ase} = 0.1$. The baseline mutation rate is equal to $m_{base} = 2.66 \cdot 10^{-9}$ (across N_{bp}=1000 bp), and carrying capacity is fixed at $K = 10^6$. In each run, the functional event list F, see Eq.1, consists of 6 identical events of the same type, chosen among type 1-Deregulation of the proliferation program (**squares**), type 3-Limit evasion (**triangles**), type 4-Resource control (**circles**), type 0-Null effect (**diamonds**). Different choices of the parameters have been explored: s ∈ {0.1, 0.2, 1.0} (**light**, **medium**} and **dark** orange), k ∈ {K, 2K, 10K} (**light**, **medium** and **dark** green) and η = 1, ν ∈ {0.7, 0.5, 0.2, 0.1} (from **lightest to darkest** mauve). For each configuration of the parameters, a total of 100 simulations have been run. Simulations are stopped either upon reaching the maximum size M or the maximum time equal to 5 years. In the **left column** M=1/10K, corresponding to an

early stopping during the expansive phase of the tumor. In the **central column** M=2K, corresponding to a stopping during an early stable phase of the tumor. In the **left column** M=10K, corresponding to a stopping during a late stable phase of the tumor. **Top row**: all runs are shown together in the same (n,D) plane, coloured by parameter values and shaped by mechanism type. **Middle row**: runs are stratified to isolate each mechanism and parameter configuration. **Bottom row**: runs are separated based on whether the maximum size M was reached before the maximum time.

The zoomed examples represented in Fig.5 illustrate the microscopic origin of these macroscopic patterns. Under pure passenger accumulation (type 0-Null effect functional events), most subclones remain extremely small, with only rare events producing detectable expansions, an example of this uncommon occurrence is visible in Fig.5 bottom-left panel. When such expansions occur, they are not driven by any intrinsic advantage of the clone, but by a purely stochastic fluctuation: it is only by chance that lineages in the surrounding population go extinct, allowing the neutral clone to drift to an unexpectedly large prevalence that is then preserved once carrying capacity is reached. This explains why these events are observed only for mutations that arise very early, during the expansive phase of the tumor evolution. By contrast, proliferative deregulation mutations (type 1-Deregulation of the proliferation program) exploit a genuine selective advantage during this same phase, which makes them consistently more likely to expand as visible in Fig. 5 top-left panel. Once the carrying capacity is reached, however, this advantage becomes ineffective: clonal sizes stabilize, and newly acquired proliferative mutations no longer produce significant expansions. As a result, both neutral-like and proliferative regimes converge to the same thin, sweep-like structures, explaining why they collapse onto the same low-n region in Fig. 4. In this sense, the framework also recovers signatures typically associated with *neutral evolution*: in [24], the authors already showed that, under neutrality, passenger mutations yield a VAF distribution heavily skewed toward very low frequencies, exactly as we observe in our simulations; however, their conclusion that such patterns imply predominantly neutral evolution has been widely criticized [55-57]. Indeed in our framework low-frequency variants arise not only from passenger mutations (type 0), but also from proliferative deregulation mutations (type 1) acquired in the stable phase, where their selective advantage cannot be expressed.

The key result of the systematic exploration of the tumor evolution under our model is that this degeneracy, caused by the resources exhaustion when the system reaches the carrying capacity, is broken only by ecological interaction terms. In particular, the susceptibility index ν, associated to type 4-Resource control functional effects, acts as the main control knob of the system. Recall that large positive values of the parameter indicate that the population is strongly impaired by competitors, whereas positive values below one indicate a weaker harming effect due to competition. Equivalently, large positive values of the parameter indicate that the population has weak resistance to competitors, whereas positive values below one indicate a stronger response to competition. The sensitivity of the model to this parameter is discussed in details in Section S3 Parameter exploration, yet it is worth noting that varying this parameter allows for spanning from complete clonal sweeps to fully branched architectures of evolution. When the tumor accumulate, at low rate, mutations associated to type 4-Resource

control functional effects with small positive values of v, the new populations have strong resistance to competition and, given the low rate of mutation, they have the time to increase their sizes by using a larger amount of the limited resources letting the system collapse into near-perfect selective sweeps, see Fig.5 bottom-right panel. Under these conditions, only a single lineage at a time can effectively persist, reproducing dynamics consistent with *linear evolution*. Conversely, for milder resistance (larger susceptibility parameters), multiple lineages can coexist and expand simultaneously, producing architectures such as that in Fig.5 top-right panel, and giving rise to *branching evolution*. These two regimes emerge from continuous parameter variations within the same mechanism, rather than from fundamentally distinct model assumptions.

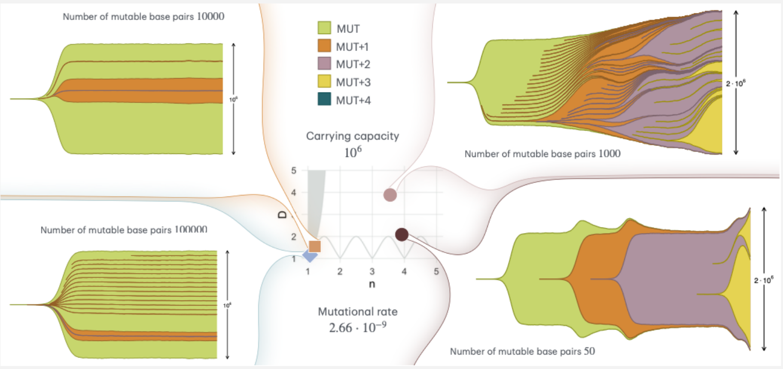

Fig.5 Examples of clonal architectures observed in the experiments. Four dots in the (n,D) plane (single simulated tumors) are illustrated through the Muller plots, coloured according to the number of mutational events associated with the clone. MUT represents the first oncogenic mutation originating the tumor. The initiating cell has a single mutation with associated functional effect of type 1-Deregulation of the proliferation program defined by the parameter sbase = 0.1, see Eq. (11). The **bottom-left panel** reports an experiment in which only type 0-Null effect (passenger) functional effects can be associated to mutations. The **top-left panel** reports an experiment in which only single type 1-Deregulation of the proliferation program functional effects can be associated to mutations (with parameter s= 0.1). The **top-right panel** reports an experiment in which six type 4-Resource control functional effects maximum can be associated to mutations, (with identical parameters (v = 0.7,η= 1), see Eq. (13)). The **lower-right panel** reports an analogous experiment with six type 4-Resource control functional effects maximum, but with parameters (v = 0.1,η= 1). Other parameters are: baseline mutational rate $m_{base} = 2.66 \cdot 10^{-9}$ per base pair, number of mutable base pairs Nbp = 10000 (upper-left panel), Nbp = 100000 (lower-left panel), Nbp = 50 (lower-right panel), Nbp = 1000 (upper-right panel), carrying capacity K = 10^6 cells.

Type 3-Limit evasion events display a complementary behaviour, strongly coupled to the stopping condition. When simulations terminate upon reaching the given maximum size, tumors tend to exhibit similar clonal nesting values and align vertically along the D axis. When simulations are instead time-limited, the points become more dispersed, although always above a minimum threshold in n. This reflects the interplay between the additional space introduced by mutations and the imposed size constraint. In the M=2K scenario, differences between parameter values are minimal: a single mutation providing an additional carrying capacity (or more) is sufficient to reach the stopping condition, leading to an accumulation of points along the line n=2. By contrast, in the M=10K scenario, only mutations

conferring large increases in available space remain localized, while weaker effects generate broader distributions. In principle, given sufficient time and repeated events, these trajectories would converge toward discrete vertical structures corresponding to the number of mutations required to reach the imposed size threshold.

Importantly, the high-diversity region of the plane (n,D) in Fig.4 cannot be populated by tumors with type 1-Deregulation of the proliferation program or type 0-Null functional capabilities alone: they are structurally confined to low-n and low-D configurations. The full experiment therefore isolates type 4-Resources control and type 3-Limit evasion as the only mechanisms capable of generating sustained intratumor heterogeneity in the stable regime, as proliferative deregulation (type 1) only governs early-time expansion dynamics.

On the results side I found the 433-436 surprising, to the level of being suspect. In Fig. 3 top left a neutral evolution is shown in a way I would not expect given the model. Principally speaking, if I have two clones, 1, 11, with p(11) = 1, and at a time t, population X_1(t) = 1000, X_11(t) = 1 and both have the same growth rate (only passenger mutations), then for t' such that X_11(t') = 1000, X_1(t') should be ~1000000, i.e. it should not be possible to retain linearly proportional population in a system with exponential growth as long as the growth rate is the shared. Unless the individual populations are log transformed before plotting, I don't understand how this is happening and it should be investigated/justified by the authors.

The reviewer's comment refers to the Muller plot that now is included in the revised Fig. 5 bottom-left panel.

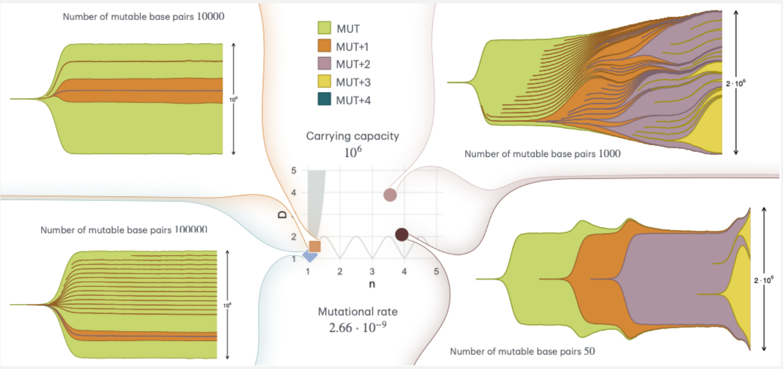

Fig.5 Examples of clonal architectures observed in the experiments. Four dots in the (n,D) plane (single simulated tumors) are illustrated through the Muller plots, coloured according to the number of mutational events associated with the clone. MUT represents the first oncogenic mutation originating the tumor. The initiating cell has a single mutation with associated functional effect of type 1-Deregulation of the proliferation program defined by the parameter sbase = 0.1, see Eq. (11). The **bottom-left panel** reports an experiment in which only type 0-Null effect (passenger) functional effects can be associated to mutations. The **top-left panel** reports an experiment in which only single type 1-Deregulation of the proliferation program functional effects can be associated to mutations (with parameter s= 0.1). The **top-right panel** reports an experiment in which six type 4-Resource control functional effects maximum can be associated to mutations, (with identical parameters (v =

0.7,η= 1), see Eq. (13)). The **lower-right panel** reports an analogous experiment with six type 4-Resource control functional effects maximum, but with parameters (v = 0.1,η= 1). Other parameters are: baseline mutational rate $m_{base} = 2.66 \cdot 10^{-9}$ per base pair, number of mutable base pairs Nbp = 10000 (upper-left panel), Nbp = 100000 (lower-left panel), Nbp = 50 (lower-right panel), Nbp = 1000 (upper-right panel), carrying capacity $K = 10^6$ cells.

In this experiment we plot the growth of a population with an overall growth rate (birth - death) equal to 0.1. The plot includes the daughter populations that can acquire only passenger type mutations (no proliferative advantage), hence all the clones share the same overall growth rate. The clones undergo exponential growth as long as carrying capacity is reached. After reaching carrying capacity, the growth is capped for all the populations and the system enters a “stable” regime in which the relative abundances are “fixed”. In a deterministic setting we could not observe a Muller plot similar to the one included in Fig. 5 bottom left panel. Indeed, as pointed out by the reviewer, the relative abundances are constantly maintained over time. But in the stochastic setting of our model, it may happen that a random fluctuation of the abundances changes significantly the relative proportions, that will be then preserved. This is an extreme and rare event, similar to the so called “black swan events”, nevertheless observable with positive probability. In section “S3 Parameter Exploration”, the reader may find a collection of experiments dedicated to understand the tumor clonal dynamics in the first expansive phase and in the later stable phase, the differences and similarities between the cases where only passenger mutations are acquired and the cases where proliferative advantages are gained. We tried to point to that section more explicitly, see lines 508-523, giving also a brief summary of the main results there reported.

A systematic exploration of the model’s parameter space has first been conducted by isolating one type of functional event at a time, allowing the role of each functional mechanism to be disentangled across a broad range of configurations, see Section S3 Parameter exploration. That analysis revealed few robust findings: 1. a clear distinction between the behavior observed during the tumor’s initial expansive phase, when resources and space are still available, and the later stable phase, when those resources and space have been depleted; 2. the possibility that passenger mutations (type 0-Null effect functional events) give rise to clones that reach detectable sizes when they arise early in the expansive phase; 3. the fact that proliferative deregulation (type 1-Deregulation of the proliferation program functional events) give rise to non-neutral dynamics only during this early phase, showing larger survival probability and expansion potential. However, if the type 1 functional event is acquired later in the stable phase, where resources and space are no more available, the clone is not able to increase its size and become detectable. Taken separately, these observations do not yet establish to which extent each different mechanism is responsible for the emergence of sustained intratumor heterogeneity.

Moreover, we revised both the Fig. 5 and the comment in the manuscript in order to better illustrate the phenomenon, see lines 560-568.

Under pure passenger accumulation (type 0-Null effect functional events), most subclones remain extremely small, with only rare events producing detectable expansions, an example of this uncommon occurrence is visible in Fig.5 bottom-left panel. When such expansions occur, they are not driven by any intrinsic advantage of the clone, but by a purely stochastic fluctuation: it is only by chance that lineages in the surrounding population go extinct, allowing the neutral

clone to drift to an unexpectedly large prevalence that is then preserved once carrying capacity is reached. This explains why these events are observed only for mutations that arise very early, during the expansive phase of the tumor evolution.

On the side of writing in general, I had the feeling the authors are mostly coming from mathematics and running the paper by a computational biologist in phylogenetics would be warranted. This is mainly evident in 128-140. As far as I can tell, LFORT is a multifurcating phylogenetic tree and does not need to be defined (potentially only in the supplement for the proof; since you in practice only consider finite depth trees, the potentially infinite depth of LFORT is in my opinion not a main matter of the paper). Similarly in 140-153 it seems that the text 4 times repeats that X_{node}(t) is a population of that node at time t, while stating that X_v is a symbol for both the ancestor population and the descendant population, based on u which is not part of the X_v symbol… Please once formalize $X_u(t)$ (which is rather unclear in 146 currently) and move the definitions of phylogenetic relationships between nodes elsewhere, or omit altogether.

The reviewer is right. We tried to follow the suggestion. We removed the parts of the text that could be considered redundant and we clarified the definition of $X_u(t)$, see lines 131-138.

The phylogenetic evolution of a tumor is described as a rooted tree τ. The generic element $u=(u_1,...,u_n)$ of the tree contains a collection of mutations and can be interpreted as a genotype, i. e. a *mutational profile*. We define $|u|:=n$ the generation of u, $p(u) = (u_1,...,u_{n-1})$ the parent of u and we will denote as $u|_j:=(u_1,...,u_j)$ the restriction up to generation j of the element u. A new genotype v branches from u at the (n+1)-th generation when an additional mutational event arises, with parent equal to u, i. e. $p(v) = v|_n = u$. We will denote as $X_u(t)$ the number of cells, with genotype u, at time t.

On the data side, as far as I can tell only the tool is available, but neither the simulated data, nor their analysis is made available. Either the results or a recipe for recreating the results using the tool should be made public.

The concern raised by the reviewer has now been addressed by extending the publicly available resources associated with the manuscript.

Specifically, we now provide:

- a Zenodo repository (https://doi.org/10.5281/zenodo.19821839) containing the complete raw outputs of all simulations used in both the main manuscript and the Supplementary Materials;
- a GitHub repository Insite (https://github.com/qBioTurin/Insite.git) containing:
    - the full simulation and analysis package;
    - example scripts illustrating how to run simulations and perform downstream analyses;
    - the processed datasets derived from the raw simulation outputs;
    - the complete code required to reproduce all figures in the manuscript from the processed data;
    - the code required to regenerate the processed datasets starting from the raw simulation outputs deposited on Zenodo;

    - a Docker image (distributed through DockerHub) with the package and all required dependencies pre-installed to facilitate reproducibility;
- the previously available GitHub repository Insite_Interface (https://github.com/qBioTurin/Insite_Interface.git) providing the graphical user interface, which builds on top of the Docker image of the simulator and remains available as a user-friendly exploratory tool for running and analysing example simulations.

The full simulation outputs, processing pipeline, and figure-generation workflow are therefore now publicly available.

Minor comments:
- Since the clonal nesting and clonal diversity are not developed here, they should go to methods, not results.

Correct, we moved the corresponding part of the text into a new section “Materials and Methods – Methods for downstream analysis of simulated tumors”. In this section we collected the description of the method for sequencing the synthetic tumor, both bulk and multiregional, as well. See lines 491-502.

- Cancer simulations tend to be computationally expensive, in particular when an interpreted language is used. A short summary of simulation and optimization compute costs should be included.

We added a dedicated analysis of computational costs based on the new set of experiments introduced in the section “Results/Exploration of Evolutionary Paradigms”. This analysis summarizes the simulation run times and optimization-related computational costs across parameter configurations. To avoid further increasing the length of the main manuscript, the analysis was included in the Supplementary Materials under the section “S3 Parameter Exploration – Experiment 3 – Run Times”, lines 1612-1625 and Fig. S3.12.

> **Run times.** We used this experiment to compile an indicative report of run times, as it represents the most complete and non-trivial experiment conducted under a controlled scenario. The experiment was run on an HPC infrastructure equipped with an OmniPath network and 2 × Intel® Xeon® CPU E5-2697 v4 processors and holding 125GB RAM. Simulations were executed in parallel (i.e., different simulations running concurrently), while run times were recorded on a per-simulation basis. On average, each simulated tumor mass requires 1.36 minutes (sd 1.04 min). However, run times are strongly influenced by the model parameters. In particular, the maximum size M has a clear impact, which is expected since it acts as a stopping criterion: the smaller M, the earlier the simulation terminates. Specifically, the average run time is 7.98 seconds (sd 2.78 s) for $M = 0.1K$, 1.72 minutes (sd 38.2 s) for $M = 2K$, and 2.24 minutes (sd 39.5 s) for $M = 10K$. This also explains the differences observed in Fig. S3.12 across functional effects: longer runs are typically associated with parameter configurations that are less likely to reach the stopping threshold (see Fig. 4).

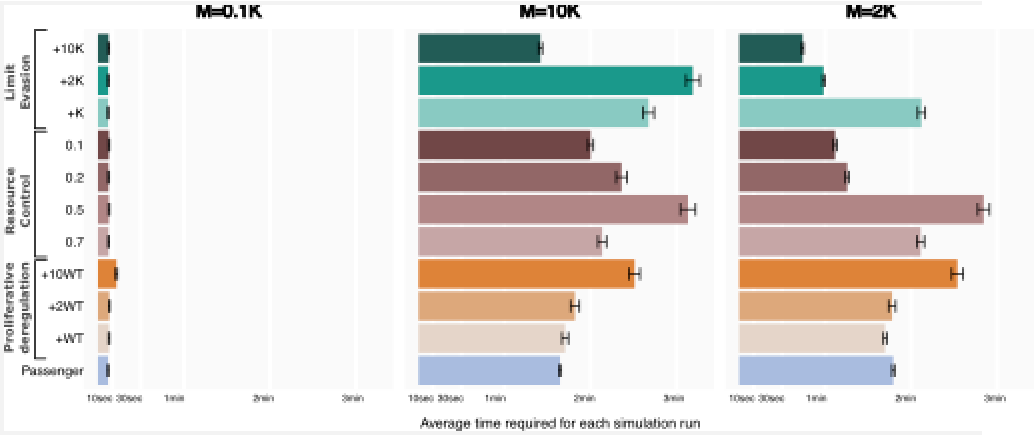

Fig. S3.12. Average running time (among the 100 independent runs) of each parameter configuration. The error bars correspond to 95% confidence intervals.

- The work presents a nice simulation web interface, however it is not publicly hosted and scientific clusters usually don't allow running custom web servers with outside access. A hosted version would be certainly an advantage of this simulator over others.

With the new Git repository for the Insite R package, users can now run large-scale experiments that are not easily managed through the simulation web interface. We are currently working on a project that will deliver: 1. a C++ reimplementation of the simulation code; 2. adaptation and optimization of the code for execution on HPC4AI, with particular attention to parallelization, job scheduling, and memory allocation; and 3. deployment of the new software on the HPC4AI servers: https://hpc4ai.unito.it/.

- I think for each node u, the ph(u) is constant? Therefore a_{ph(u)} does not have to explicitly include (ph) and you can keep a(u) and b(u) without the replacement for a, b at 328? Also I think you're referring to the application of a_{ph(u)} given X(t) in this line?

The reviewer is right. For each node u, the $ph(u)$ is constant over time, while $a_{ph(u)}$ and $b_{ph(u)}$ are size dependent hence they are calculated given $X(t)$. We decided to keep the notation $a_{ph(u)}$ and $b_{ph(u)}$ as the function ph is not injective. We deleted the replacement with a and b. We kept $a_{ph(u)}$ and $b_{ph(u)}$ in Eq. 16.

- 338 the M is I think related to m in 343 but it's not very clear, also I'm not sure if p_n at 336 is needed and you already use p at 137.

The reviewer is right. We corrected the text. Lines 414-416.

- 228-238 - it seems that most of this text comes from explicitly treating passenger mutation differently. Since you already have variable parameter effects, could you not just say that a neutral mutation is a parameterless one and a parent differs from a child exactly by one mutation?

The definition of the phenotype function, given in Eq. (3) is motivated by the necessity to include the functional redundancy of the gene network: if a new mutation is acquired in a different location but it concurs to damage a functional path that has been already damaged, the effect is neglected and hence the phenotype is unchanged.

- 286 \mu seems to be a mutational rate from context, but not specified.

We added the definition, line 274:

> where $\mu_{\mathcal{ph}(p(u))}$ is the mutation rate associated to the phenotype of the parent of u.

- 295 - what is \Omega? Also I think you are switching between a list of univariate functions and a multivariate function.

We deleted those lines. Following the previous comments, we decided they were confusing by adding unnecessary mathematical details.

- 910-914 unclosed brackets

We corrected the equations.

- In the default parameters of the simulation the mutation probability is set to 2.66×10-9, that seems rather low?

This value is given to the baseline mutational parameter m_{base} and has to be interpreted as the somatic mutation rate per base pair per division in normal human cells, lines 332-334. We refer to [50] and [51].

- Despite that, a simulation with 10 cores and otherwise default parameters has not terminated in an hour and there's no progress reporting or logging, so I was not able to tell if there was any issue or if I was supposed to wait longer. (Tested on a node with 32 4Ghz cores and 128GB RAM).

The issue was caused by dependency conflicts between R libraries occurring during Docker image construction on the user side. Importantly, these errors did not prevent the NextJS interface from launching correctly, making the problem appear as if the simulation itself was not terminating.

To avoid this issue, we reorganized the simulation framework into a dedicated R package ("Insite", available at the GitHub repository https://github.com/qBioTurin/Insite.git) and we now directly provide a pre-built Docker image containing the package together with all required dependencies already installed. The image has been built and tested by us and is distributed through DockerHub.

—-------

Reviewer #3:

This manuscript presents a stochastic tumor evolution framework that represents clonal lineages on a rooted ordered tree and maps genotypes to phenotypes via "functional events" (e.g., proliferative deregulation, mutation-rate increase, limit evasion, resource control), with resource-mediated competition and carrying-capacity constraints. The authors also provide a simulation algorithm and a GUI intended to facilitate exploration and reproducibility . They demonstrate that different evolutionary "paradigms" can arise under different parameter settings and validate against empirical tumor datasets using clonal diversity and clonal nesting metrics. Overall, the paper is well-written, the software and the problem are very interest, just unfortunate that I was unable to run it.

We thank the reviewer for the valuable comments. It is correct that the web interface had to be fixed. The problem was related to dependency conflicts between R libraries during Docker image construction. We fixed it and we worked to give the reader all the software and data to reproduce the results illustrated in the paper.

Here it follows the list of responses to the detailed comments. The reviewers' original comments are reproduced in *yellow italics*, the authors' responses are presented in plain black text, and excerpts from the manuscript are shown with gray background. All the reported line numbers, figure numberings and equation numberings are referred to the clean copy of the revised manuscript.

Major comments

1) The code seems interesting. However, I was unable to run it successfully on my machine.

My system
2.3 GHz Quad-Core Intel Core i7
Intel Iris Plus Graphics 1536 MB
16 GB 3733 MHz LPDDR4X
macOS 26.3 (25D125)

Docker
Docker version 29.2.1, build a5c7197
Docker Compose version v5.0.2

I followed the instructions from GitHub:
Clone the git with
git clone git@github.com:qBioTurin/CancerSimulationInterface.git
cd CancerSimulationInterface

Then run (MacOS/Linux):
docker compose up --build

After running docker compose up --build, my terminal showed:

Attaching to interface-1, simulator-1
*simulator-1 | * Serving Flask app 'main'*

*simulator-1 | * Debug mode: off*
simulator-1 | WARNING: This is a development server. Do not use it in a production deployment. Use a production WSGI server instead.
*simulator-1 | * Running on all addresses (0.0.0.0)*
*simulator-1 | * Running on http://127.0.0.1:5000*
*simulator-1 | * Running on http://172.18.0.3:5000*
simulator-1 | Press CTRL+C to quit
interface-1 |
interface-1 | > cancersimulator@0.1.0 start
interface-1 | > next start
interface-1 |
interface-1 | ▲ Next.js 15.3.3
interface-1 | - Local: http://localhost:3000
interface-1 | - Network: http://172.18.0.2:3000
interface-1 |
interface-1 | ✓ Starting…
interface-1 | ✓ Ready in 1021ms

I then opened http://localhost:3000 in my browser. The user interface loaded, but with the default parameters, when I clicked “Run Simulation,” the button showed a spinning circle and the app appeared to get stuck. I waited for almost two hours and nothing happened.

In the terminal, I repeatedly saw the following message:
simulator-1 | 172.18.0.2 - - [03/Mar/2026 22:27:38] "GET /check_percentage?checkpoints=50 HTTP/1.1" 500 -

I also tried reducing the simulated time span to 4 (in case the issue was the simulation length), but the same behavior occurred. Also, the progress bar in the interface did not move at all during this time. How long is a typical run expected to take with the default settings? Have you tested it on different machines, and could you provide approximate run-time metrics? (I did not change any parameters, including the advanced settings such as the number of threads.)

The behavior reported by the reviewer was caused by dependency conflicts between R libraries during Docker image construction on the user side, rather than by the simulation itself. Since these dependency issues did not stop the NextJS interface from launching, the application appeared to be running normally while the backend simulation workflow was effectively stalled. To address this issue, we substantially reorganized the software distribution pipeline. The simulation framework was packaged into a dedicated R package “Insite”, available in a newly created GitHub repository https://github.com/qBioTurin/Insite.git, and we now directly provide a pre-built Docker image containing the package together with all required dependencies already installed and tested. The image is distributed through DockerHub and is used directly by the graphical interface repository, avoiding local dependency-resolution problems during image construction. We also added the explicit testing information for multiple operating systems and architectures, reported in the GitHub README https://github.com/qBioTurin/Insite_Interface.git;

For reproducibility, I strongly encourage:

• a tagged release corresponding to the submitted manuscript;

We have created and published a tagged release of the repositories corresponding exactly to the version used in the submitted manuscript (v0.1.0), ensuring full versioned reproducibility.

• a minimal “reproduce figures” script in addition to the GUI;

We have extended the publicly available resources associated with the manuscript. Specifically, we now provide:

- a Zenodo repository (https://doi.org/10.5281/zenodo.19821839) containing the complete raw outputs of all simulations used in both the main manuscript and the Supplementary Materials;
- a GitHub repository Insite (https://github.com/qBioTurin/Insite.git) containing:
  - the full simulation and analysis package;
  - example scripts illustrating how to run simulations and perform downstream analyses;
  - the processed datasets derived from the raw simulation outputs;
  - the complete code required to reproduce all figures in the manuscript from the processed data;
  - the code required to regenerate the processed datasets starting from the raw simulation outputs deposited on Zenodo;
  - a Docker image (distributed through DockerHub) with the package and all required dependencies pre-installed to facilitate reproducibility;
- the previously available GitHub repository Insite_Interface (https://github.com/qBioTurin/Insite_Interface.git) providing the graphical user interface, which builds on top of the Docker image of the simulator and remains available as a user-friendly exploratory tool for running and analysing example simulations.

The full simulation outputs, processing pipeline, and figure-generation workflow are therefore now publicly available.

• documentation of compute requirements and run times for the main experiments.

A dedicated analysis of simulation run times has been included in the Supplementary Section “S3 Parameter Exploration – Experiment 3 – Run Times” (lines 1612-1625 and Fig. S3.12), where we report the computational time required for the main experiments under representative parameter settings.

> **Run times.** We used this experiment to compile an indicative report of run times, as it represents the most complete and non-trivial experiment conducted under a controlled scenario. The experiment was run on an HPC infrastructure equipped with an OmniPath network and 2 × Intel® Xeon® CPU E5-2697 v4 processors and holding 125GB RAM. Simulations were executed in parallel (i.e., different simulations running concurrently), while run times were recorded on a per-simulation basis. On average, each simulated tumor mass requires 1.36 minutes (sd 1.04 min). However, run times are strongly influenced by the model parameters. In particular, the maximum size M has a clear impact, which is expected since it acts as a stopping criterion: the smaller M, the earlier the simulation terminates. Specifically, the average run time is 7.98 seconds (sd 2.78 s) for $M = 0.1K$, 1.72 minutes (sd 38.2 s) for $M = 2K$, and 2.24 minutes (sd 39.5 s) for $M = 10K$. This also explains the differences observed in Fig. S3.12 across functional effects: longer runs are typically associated with

parameter configurations that are less likely to reach the stopping threshold (see Fig. 4).

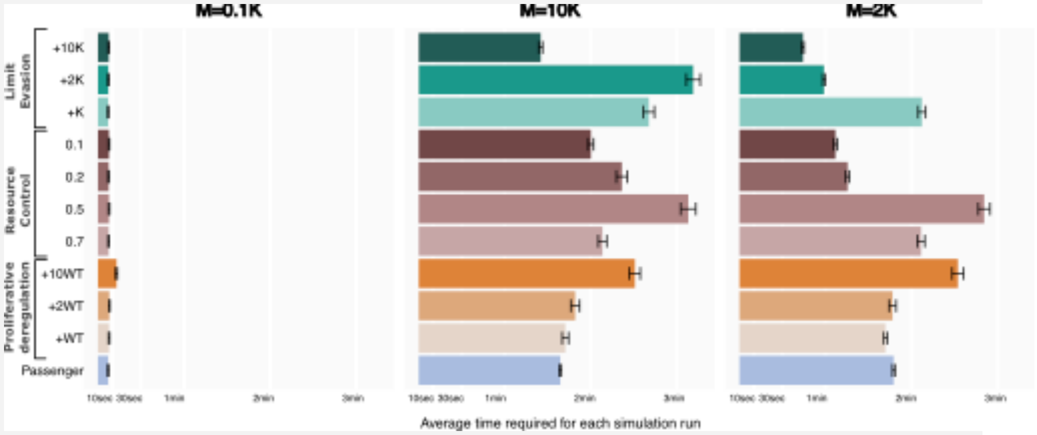

Fig. S3.12. Average running time (among the 100 independent runs) of each parameter configuration. The error bars correspond to 95% confidence intervals.

Regarding compute requirements in the GitHub repository Insite_Interface (https://github.com/qBioTurin/Insite_Interface.git), we now provide a summary of the tested execution environments, including operating systems and CPU architectures, to ensure reproducibility across common platforms. The tool was successfully tested under the following configurations: Windows 11 24H2 (x64), macOS Tahoe 26.0.1 (Apple Silicon, M1/M4), and Rocky Linux 9.7 (x64).

2) Clarify what is meant by “single-cell resolution”

The manuscript states that the “simulator tracks tumor evolution at single-cell resolution, recording for each cell its genotype and phenotype”. However, the described simulation step updates population sizes via multinomial sampling for each clone and then spawns new clones via Poisson sampling. That is a clone/population-level simulation (which is fine), but it is not obvious that individual-cell are explicitly tracked throughout time.

The reviewer is right. We track the size of each clone for which we have the identical genotype of the composing cells. We modified the text accordingly to this comment, line 463:

> The simulator tracks tumor evolution at single clone resolution, recording for each cell its genotype and phenotype.

3) Biological interpretability of “functional events” and mapping to hallmark-like mechanisms

The phenotype mapping is grounded in hallmark-style functional categories and collapses them into five mechanisms plus “null effect”. This is conceptually appealing, but for readers it will be important to understand:
• how parameter ranges for each mechanism were chosen (especially “resource control” susceptibility/offensiveness and “limit evasion” as added capacity);

The parameter ranges were exhaustively explored with a set of experiments whose results are summarized in the revised supplementary section “S3 Parameter exploration”. The section contains the results from 3 experiments. The first one was designed to explore the chances of survival of single subclones, characterized by different types of functional events in a wide range of the corresponding

parameters. In the second experiment the setting with multiple subclones of the same type is explored and the resulting synthetic tumors are explored from the point of view of the genetic characteristics of the clones. Finally the third experiment, which has been added in the main manuscript in the revised version of the section “Results – Exploration of evolutionary paradigms” aims at elucidating the kind of tumors that the different types of functional effects can grow.

In particular the type 4-Resource control functional events’ parameters have been chosen following both biological motivation (as the chosen configurations correspond to interpretable competitive strategies) and the evidences accumulated in previous experiments that showed the susceptibility index was the dominant parameter shaping resource based advantages.

In order to provide to the reader a better understanding of the parameters and the resulting clonal dynamics of the simulated tumor we revised both the supplementary section “S3 Parameter exploration” and the section of the main text “Results – Exploration of the evolutionary paradigms. We refer explicitely the reader to section “S3 Parameter exploration” providing also a summary of the findings there illustrated, we include new experiments (with an accompanying figure numbered now as Fig. 4) and we try to explain better the evolutionary patterns illustrated in Fig. 5.

> A systematic exploration of the model’s parameter space has first been conducted by isolating one type of functional event at a time, allowing the role of each functional mechanism to be disentangled across a broad range of configurations, see Section S3 Parameter exploration. That analysis revealed few robust findings: 1. a clear distinction between the behavior observed during the tumor’s initial expansive phase, when resources and space are still available, and the later stable phase, when those resources and space have been depleted; 2. the possibility that passenger mutations (type 0-Null effect functional events) give rise to clones that reach detectable sizes when they arise early in the expansive phase; 3. the fact that proliferative deregulation (type 1-Deregulation of the proliferation program functional events) give rise to non-neutral dynamics only during this early phase, showing larger survival probability and expansion potential. However, if the type 1 functional event is acquired later in the stable phase, where resources and space are no more available, the clone is not able to increase its size and become detectable. Taken separately, these observations do not yet establish to which extent each different mechanism is responsible for the emergence of sustained intratumor heterogeneity.
>
> To address this, we turn to the integrated experiment summarized in Fig.4, which condenses many simulated tumors into a unified (n,D) representation. In this setting, each simulated tumor can accumulate mutations that are associated to six functional events maximum, all of the same type and with identical parameters. This design enables a controlled, differential comparison between functional mechanisms while still accounting for cumulative effects of repeated alterations of the same type. Each point in the figure therefore represents the outcome of a full stochastic trajectory under a specific mechanism and parameter choice. Simulations are stopped either upon reaching the maximum size M or the maximum time equal to 5 years. Three different values of M have been explored. A value smaller than the carrying capacity K, namely M=1/10K, which corresponds to an early stopping during the *expansive* phase of the tumor. Two values larger than the carrying capacity, M=2K and M=10K, corresponding to a stopping during

an *early stable* phase of the tumor and a *late stable* phase of the tumor, respectively.

A first, clear separation emerges between the behaviors exhibited at the expansive phase and at the two stable phases. In the expansive regime, only the accumulation of mutations associated to type 1-Deregulation of the proliferation program is able to drive any appreciable departure from the baseline point (n=1, D=1). As predicted by [41], if the selective advantage s is sufficiently large, the evolution follows sequential selective sweeps and (dark) orange squares align on the light gray curves calculated in [41]. Diversity stays low and the number of dominant clones never exceeds n ≈ 2, even when the system is observed across all replicates. In this regime, the framework naturally recovers *punctuated*-like dynamics: early proliferative events generate rapid expansions, after which the system quickly saturates and no further diversification occurs. This picture changes radically in the stable regime. Once resource saturation and ecological constraints become relevant, proliferative deregulation (type 1) loses explanatory power: its trajectories remain confined to the same low-diversity sweep structure observed in the early phase. In contrast, mechanisms based on resource control (type 4) and limit evasion (type 3) become the dominant drivers of evolution. They progressively occupy the entire (n,D) plane, generating both high-diversity configurations and complex branching patterns. The transition is not gradual in terms of qualitative behaviour: it is a regime shift in which the relevant explanatory mechanisms are different. Passenger-only configurations (type 0), by contrast, remain concentrated at the baseline point (n=1, D=1) across all phases, reflecting the fact that the accumulation of neutral mutations alone does not generate dominant clones at the population scale.

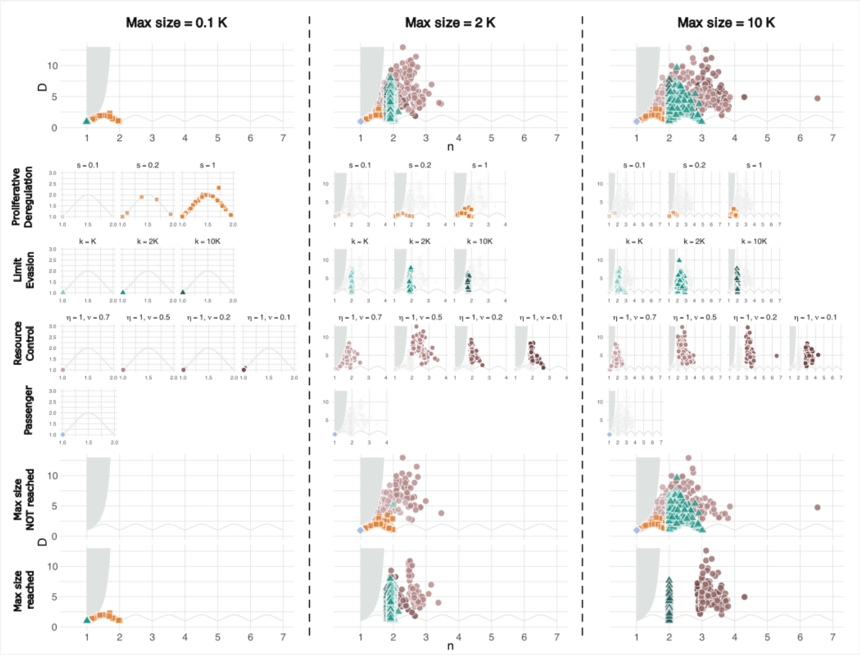

Fig 4. Simulated tumors on the (n,D) plane. Each point represents a single independent simulation run; the shape indicates the type of primary mechanism included, and the colour denotes the specific parameter configuration. Simulations start from a single cell already

carrying a mutation associated to type 1-Deregulation of the proliferation program primary mechanism with parameter $s_{\beta ase}$ = 0.1. The baseline mutation rate is equal to m_{base} = 2.66 · 10^{-9} (across N_{bp}=1000 bp), and carrying capacity is fixed at K = 10^6. In each run, the functional event list F, see Eq.1, consists of 6 identical events of the same type, chosen among type 1-Deregulation of the proliferation program (**squares**), type 3-Limit evasion (**triangles**), type 4-Resource control (**circles**), type 0-Null effect (**diamonds**). Different choices of the parameters have been explored: s ∈ {0.1, 0.2, 1.0} (**light**, **medium**} and **dark** orange), k ∈ {K, 2K, 10K} (**light**, **medium** and **dark** green) and η = 1, ν ∈ {0.7, 0.5, 0.2, 0.1} (from **lightest to darkest** mauve). For each configuration of the parameters, a total of 100 simulations have been run. Simulations are stopped either upon reaching the maximum size M or the maximum time equal to 5 years. In the **left column** M=1/10K, corresponding to an early stopping during the expansive phase of the tumor. In the **central column** M=2K, corresponding to a stopping during an early stable phase of the tumor. In the **left column** M=10K, corresponding to a stopping during a late stable phase of the tumor. **Top row**: all runs are shown together in the same (n,D) plane, coloured by parameter values and shaped by mechanism type. **Middle row**: runs are stratified to isolate each mechanism and parameter configuration. **Bottom row**: runs are separated based on whether the maximum size M was reached before the maximum time.

The zoomed examples represented in Fig.5 illustrate the microscopic origin of these macroscopic patterns. Under pure passenger accumulation (type 0-Null effect functional events), most subclones remain extremely small, with only rare events producing detectable expansions, an example of this uncommon occurrence is visible in Fig.5 bottom-left panel. When such expansions occur, they are not driven by any intrinsic advantage of the clone, but by a purely stochastic fluctuation: it is only by chance that lineages in the surrounding population go extinct, allowing the neutral clone to drift to an unexpectedly large prevalence that is then preserved once carrying capacity is reached. This explains why these events are observed only for mutations that arise very early, during the expansive phase of the tumor evolution. By contrast, proliferative deregulation mutations (type 1-Deregulation of the proliferation program) exploit a genuine selective advantage during this same phase, which makes them consistently more likely to expand as visible in Fig. 5 top-left panel. Once the carrying capacity is reached, however, this advantage becomes ineffective: clonal sizes stabilize, and newly acquired proliferative mutations no longer produce significant expansions. As a result, both neutral-like and proliferative regimes converge to the same thin, sweep-like structures, explaining why they collapse onto the same low-n region in Fig. 4. In this sense, the framework also recovers signatures typically associated with *neutral evolution*: in [24], the authors already showed that, under neutrality, passenger mutations yield a VAF distribution heavily skewed toward very low frequencies, exactly as we observe in our simulations; however, their conclusion that such patterns imply predominantly neutral evolution has been widely criticized [55-57]. Indeed in our framework low-frequency variants arise not only from passenger mutations (type 0), but also from proliferative deregulation mutations (type 1) acquired in the stable phase, where their selective advantage cannot be expressed.

The key result of the systematic exploration of the tumor evolution under our model is that this degeneracy, caused by the resources exhaustion when the system reaches the carrying capacity, is broken only by ecological interaction terms. In particular, the susceptibility index ν, associated to type 4-Resource

control functional effects, acts as the main control knob of the system. Recall that large positive values of the parameter indicate that the population is strongly impaired by competitors, whereas positive values below one indicate a weaker harming effect due to competition. Equivalently, large positive values of the parameter indicate that the population has weak resistance to competitors, whereas positive values below one indicate a stronger response to competition. The sensitivity of the model to this parameter is discussed in details in Section S3 Parameter exploration, yet it is worth noting that varying this parameter allows for spanning from complete clonal sweeps to fully branched architectures of evolution. When the tumor accumulate, at low rate, mutations associated to type 4-Resource control functional effects with small positive values of v, the new populations have strong resistance to competition and, given the low rate of mutation, they have the time to increase their sizes by using a larger amount of the limited resources letting the system collapse into near-perfect selective sweeps, see Fig.5 bottom-right panel. Under these conditions, only a single lineage at a time can effectively persist, reproducing dynamics consistent with *linear evolution*. Conversely, for milder resistance (larger susceptibility parameters), multiple lineages can coexist and expand simultaneously, producing architectures such as that in Fig.5 top-right panel, and giving rise to *branching evolution*. These two regimes emerge from continuous parameter variations within the same mechanism, rather than from fundamentally distinct model assumptions.

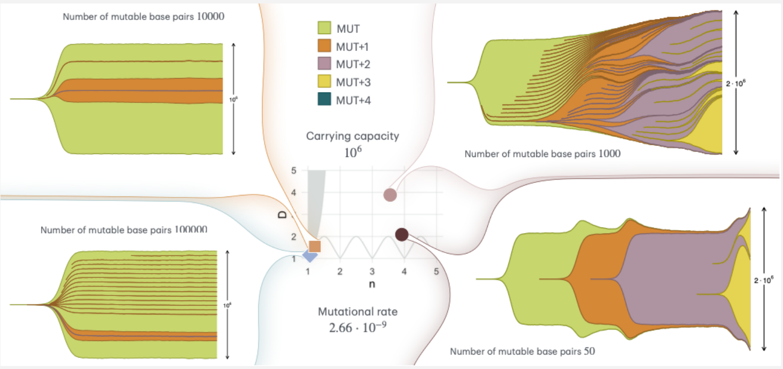

Fig.5 Examples of clonal architectures observed in the experiments. Four dots in the (n,D) plane (single simulated tumors) are illustrated through the Muller plots, coloured according to the number of mutational events associated with the clone. MUT represents the first oncogenic mutation originating the tumor. The initiating cell has a single mutation with associated functional effect of type 1-Deregulation of the proliferation program defined by the parameter sbase = 0.1, see Eq. (11). The **bottom-left panel** reports an experiment in which only type 0-Null effect (passenger) functional effects can be associated to mutations. The **top-left panel** reports an experiment in which only single type 1-Deregulation of the proliferation program functional effects can be associated to mutations (with parameter s= 0.1). The **top-right panel** reports an experiment in which six type 4-Resource control functional effects maximum can be associated to mutations, (with identical parameters (v = 0.7,η= 1), see Eq. (13)). The **lower-right panel** reports an analogous experiment with six type 4-Resource control functional effects maximum, but with parameters (v = 0.1,η= 1). Other parameters are: baseline mutational rate m_{base} = $2.66{\cdot}10^{-9}$ per base pair, number of mutable base pairs Nbp = 10000 (upper-left panel), Nbp = 100000 (lower-left panel), Nbp = 50 (lower-right panel), Nbp = 1000 (upper-right panel), carrying capacity K = 10^6 cells.

Type 3-Limit evasion events display a complementary behaviour, strongly coupled to the stopping condition. When simulations terminate upon reaching the given maximum size, tumors tend to exhibit similar clonal nesting values and align vertically along the D axis. When simulations are instead time-limited, the points become more dispersed, although always above a minimum threshold in n. This reflects the interplay between the additional space introduced by mutations and the imposed size constraint. In the M=2K scenario, differences between parameter values are minimal: a single mutation providing an additional carrying capacity (or more) is sufficient to reach the stopping condition, leading to an accumulation of points along the line n=2. By contrast, in the M=10K scenario, only mutations conferring large increases in available space remain localized, while weaker effects generate broader distributions. In principle, given sufficient time and repeated events, these trajectories would converge toward discrete vertical structures corresponding to the number of mutations required to reach the imposed size threshold.

Importantly, the high-diversity region of the plane (n,D) in Fig.4 cannot be populated by tumors with type 1-Deregulation of the proliferation program or type 0-Null functional capabilities alone: they are structurally confined to low-n and low-D configurations. The full experiment therefore isolates type 4-Resources control and type 3-Limit evasion as the only mechanisms capable of generating sustained intratumor heterogeneity in the stable regime, as proliferative deregulation (type 1) only governs early-time expansion dynamics.

Differently, the parameters used in the experiments illustrated in Fig. 6 have been estimated through the procedure described in lines 657-667 and illustrated in Fig. S3.13.

• whether the "relative frequencies" of events (r) are intended to reflect biology, sequencing panels, or are simply scenario knobs.

The relative frequencies of functional events in Fig. 6 have been estimated through the procedure described in lines 657-667. They can be interpreted as the sizes of the genomic regions that can harbor mutations associated with each functional effect. However, if sequencing was performed using a panel, these frequencies must be rescaled according to the size of the panel itself.

4) Spatial structure and interaction assumptions should be foregrounded in the main text

A key simplifying assumption is that "heterogeneity is always present," meaning every cell competes/interacts with all others (global mixing). This is a major modeling choice with direct implications for clonal interference, coexistence, and the interpretation of "spatial" patterns inferred from simulated multi-region sequencing.

This limitation should be stated explicitly in the main paper (not only the supplement), and the authors should discuss which results are expected to be robust vs. which might change under

local-neighborhood interactions. The discussion already contrasts spatial geometry vs resource control; it would help to connect that argument more directly to this assumption.

We agree with the reviewer that the fact that the model is for a well-mixed population of cells should be stated clearly. We added the information at the very beginning of the description of the model, see lines 126-129.

> Note that the model is for a well-mixed population of cells, hence it is not including a spatial component that accounts for segregation or separation of different cellular populations.

Moreover, we included more references on spatially segregated models [39, 40] and added a discussion on the differences between well-mixed population models and models with explicit spatial geometry but no limitations in resources and space, see lines 774-818. Our proposed model points to the type 4-Resource control and type 3-Limit evasion functional events as the primary candidate mechanism for the emergence of ITH. Indeed, we believe that there is biological evidence that tumor evolution happens in a system that is able to overcome strong limitations both in space and shared resources. Hence we propose here a model that studies the mechanisms classically proposed in the literature, such as the “fitness increase” of the cancerous cells, in the context of a system with limited resources (limited carrying capacity). We try to explain that this condition makes the birth rates increase ineffective, as the limitation of space and resources cap the growth of the population which should acquire other capabilities such as the type 4 and type 3 described in our model and very well established in the literature as hallmarks of cancer. Lines 774-818:

> A major limitation of many tumor evolution models lies in their difficulty in generating heterogeneous cell populations that provide a realistic representation of intratumor heterogeneity (ITH). Two main modelling strategies may be considered to address this issue. The first consists in introducing a spatial structure for the environment in which cells proliferate, so as to promote the segregation of the tumor mass into distinct subpopulations, see for example [39-41]. Under assumptions ensuring unbounded growth, with no saturation of either space or available resources, each spatially segregated niche gives rise to a distinct evolutionary trajectory, thereby leading to increasing genetic diversity. The resulting tumor exhibits a controllable degree of ITH, determined by the extent of segregation among the different subpopulations. A second approach, on which we focus here inspired by [77], consists in considering a well-mixed population of cells with growth limitations and capable of exhibiting different mechanisms of resources exploitation. Both the growth limitation and the different resources exploitations are necessary to show modelled tumors with genetic heterogeneity. Indeed, a growth constraint alone tends to reduce genetic variability, since saturation of the limited resources yields a system in which the relative frequencies of the subclones present at the time the maximum population size is reached can no longer change, thereby fixing the genetic composition at all subsequent times. The model should therefore explicitly incorporate mechanisms of differential exploitation of the limited resources (the type 4-Resource control functional effect), under which subpopulations able to recruit a larger share of the scarce resources can achieve greater growth than others. A similar attempt has been proposed in [42], where a double growth-constraint mechanism acting both on the total population size (global confinement) and on individual subclones (local

confinement) is used to mimic competition for shared resources, thereby generating genetically heterogeneous tumors. However, we believe that the ability to evade growth constraints and the ability to differentially dispose of the shared resources have to be mapped into different parts of the model. Moreover we think that those mechanisms should be clone-specific instead of shared by all the population. Indeed, those capabilities can be biologically thought as emerging from the specific set of genetic alterations that have been accumulated by the single clone.

The two approaches to modelling here discussed, the spatially organized population and the well-mixed but subject to competition for limited resources population, lead to genetically heterogeneous tumors though diverging conceptually: we argue that it is not the geometry of space itself that governs tumor evolution, but rather the control and distribution of shared resources. In our framework, competition arises from differential access to these resources and can be modulated by new driver mutations, thus the creation of new spatial compartments is not required to explain clonal diversity. Still, these spatially explicit models remain valuable, and for tumor types where physical architecture plays a dominant role, their principles could be seamlessly integrated into our simulator, further demonstrating its flexibility and generality. It would also be interesting to explore models that are neither fully well-mixed nor fully spatially segregated, but instead display an intermediate degree of local-neighborhood interactions. In this setting, it would be important to determine which results are found to be robust.

Minor comments / edits

“proliferative” appears corrupted in figure 1 text; please check figure label

Thank you for spotting this, we corrected it.

The stopping criterion uses “detectable size” assumptions (e.g., 1 cm³ ~ 10^9 cells) and then adjusts because the model tracks only alive cells. Please state the implied alive fraction (even approximately) or cite justification.

We consider that 1 cm^3 corresponds to 10^9 cells are to be included, of those we track 10^8, hence approximately 1/10 of the mass is considered alive. We added this comment on lines 644-650:

Though our model only traces alive cells, which are a fraction of the overall tumor mass. Hence stopping size has been set to 10^8 for kidney, mesothelioma, breast, and lung, and to 10^7 for uveal, corresponding to the assumption that 10$\%$ of tumor cells are alive.
