# Supplementary material for: A new cancer progression model: From synthetic tumors to real data and back
Source: PLoS Comput Biol. 2026 Jun 24;22(6):e1013991. doi: 10.1371/journal.pcbi.1013991 (PMC13327523; doi:10.1371/journal.pcbi.1013991)
Supplement: Peer review history

# Peer review: Decision Letter 0

**DOI:** 10.1371/journal.pcbi.1013991.r001

**Authors:** Pedro Mendes, Guillermo Lorenzo

11 Mar 2026

PCOMPBIOL-D-26-00268

A new cancer progression model: from synthetic tumors to real data and back

PLOS Computational Biology

Dear Dr. Sirovich,

Thank you for submitting your manuscript to PLOS Computational Biology. After careful consideration, we feel that it has merit but does not fully meet PLOS Computational Biology's publication criteria as it currently stands. Therefore, we invite you to submit a revised version of the manuscript that addresses the points raised during the review process.

Please submit your revised manuscript by May 11 2026 11:59PM. If you will need more time than this to complete your revisions, please reply to this message or contact the journal office at ploscompbiol@plos.org. When you're ready to submit your revision, log on to https://www.editorialmanager.com/pcompbiol/ and select the 'Submissions Needing Revision' folder to locate your manuscript file.

Please include the following items when submitting your revised manuscript:

* A letter that responds to each point raised by the editor and reviewer(s). You should upload this letter as a separate file labeled 'Response to Reviewers'. This file does not need to include responses to formatting updates and technical items listed in the 'Journal Requirements' section below.

* A marked-up copy of your manuscript that highlights changes made to the original version. You should upload this as a separate file labeled 'Revised Manuscript with Track Changes'.

* An unmarked version of your revised paper without tracked changes. You should upload this as a separate file labeled 'Manuscript'.

If you would like to make changes to your financial disclosure, competing interests statement, or data availability statement, please make these updates within the submission form at the time of resubmission. Guidelines for resubmitting your figure files are available below the reviewer comments at the end of this letter

We look forward to receiving your revised manuscript.

Kind regards,

Guillermo Lorenzo

Academic Editor

PLOS Computational Biology

Pedro Mendes

Section Editor

PLOS Computational Biology

**Additional Editor Comments:**

Dear authors,

I would like to thank you for submitting your work to PLOS Computational Biology. Your manuscript has been reviewed by three independent referees. While all of them have seen the value in your work, they have also suggested several revisions that are required before considering the manuscript for publication.

Special attention should be given to the organization of the manuscript, the clarity of language describing novel concepts and the mathematical formulation in the proposed modeling framework, the completeness in the explanation of central assumptions and biological interpretations of the results, the contextualization of the presented work with respect to the literature in the field, the description of the figures in their accompanying captions, and the reproducibility of the work.

We look forward to receiving your revised manuscript.

Sincerely,

GL

**Journal Requirements:**

1) We ask that a manuscript source file is provided at Revision. Please upload your manuscript file as a .doc, .docx, .rtf or .tex. If you are providing a .tex file, please upload it under the item type u2018LaTeX Source Fileu2019 and leave your .pdf version as the item type u2018Manuscriptu2019.

2) We have noticed that you have uploaded Supporting Information files, but you have not included a list of legends. Please add a full list of legends for your Supporting Information files after the references list.

**Reviewers' comments:**

Reviewer's Responses to Questions

**Comments to the Authors:**

**Please note here if the review is uploaded as an attachment.**

Reviewer #1: Review for A new cancer progression model: from synthetic tumors to real data and back

Authors introduce a very interesting evolutionary and ecological modeling framwork which consolidates many ideas from previous agent-based modeling papers in cancer, as well as conceptual ideas from the cancer hallmarks manuscripts. The figures are very nice, and the methods are very nice, however the results only show a small and incomplete picture that is unlikely to be reproducible. A new version of the manuscript should work harder to explain the figures, show how the results are generalizable beyond a single simulation, and to report the parameters used in each simulations.

Comments

1. Line 115: “A single phenotype can be determined by different genotypes” – is “determined” the right word here?

2. Line 184: “a” repeated twice, “a a boost”

3. Equation 4 isn’t sufficiently described – it’s not clear to me if this is a size-dependent branching process, or a variation with included phenotypic evolution. Secondly, please define SDBD in equation 4. Should this be SDBP?

4. Line 383: define VCF at first use.

5. I think it would be helpful to expand the figure captions to explain the cards which indicate the type of functional advantage considered, to ensure there is no misunderstandings. I’d like to further understand the difference between Fig 3 top right and Fig 3 top left – I believe it’s a functional advantage (multiplier??) for proliferation for each subsequent mutation – perhaps capped at N=2 total mutations? If so, the result that similar patterns as neutral evolution can be observed in non-neutral is also shown in a similar model in previous research (DOI: 10.1038/s41467-021-22123-1), related to lines 433-448 in the manuscript. The mechanism in that research is spatial competition, which I don’t think is considered in Figure 3. Can the authors clarify this, and why their model isn’t similar to non-neutral non-spatial patterns of diversity?

6. Again, in figure 4 it’s not really clear to me what the cards mean. What are the small numbers (0.01, 0.01, …) and the large numbers (0, 6, 5, 4, …) indicating? Are these simulations’ parameters inferred from data? How is this procedure done?

7. Is figure 5 a result or a schematic? The axes are not labelled, so I’m not clear what’s being shown here. I also don’t know what the rows/columns of the table indicate, or what parameters were used here.

Reviewer #2: The manuscript presents a new model of development of intra-tumor-heterogeneity. The work follows in the trajectory of extending stochastic process (population-based) models with phenotype-derived fitness properties.

As there is already a plethora of existing models, the main challenge for a model is to demonstrate this model is useful, (i) either by extending our understanding of the modelled system on a human-understandable level, or (ii) by being able to create testable predictions about the system. In both cases I feel the answer is potentially yes, with caveats.

Major comments:

In terms of the model, the authors compare the results to [38, 39], both of which are simpler models, focused on demonstrating the origin of ITH from spatial structure of the environment. The model of Volpatto et. al. uses a more complex double-stochastic process with explicitly modelled mechanisms for 5 functional events. The choice of modelling framework and modelled phenotypes results in a, comparatively speaking, complex mathematical model. Explicit modelling of hallmarks in a stochastic process is by itself not a novelty and is present e.g. in tugHall model (Nagornov 2020). I think this work should be at least shortly compared.

Utility of the model therefore has to be demonstrated, since the model is quite complex and not that novel. The authors argue that spatial segregation is not needed to reconstruct various patterns of heterogeneity. This may be true, but this model utilizes carrying capacity and limit evasion, which principally falls into the same category. Generally, for heterogeneity to arise, the systems must have a growth limitation that is non-linearly dependent on the population size. The effects of population limitation on diversity are discussed in e.g. “Hu, 2017, A population genetics perspective on the determinants of intra-tumor heterogeneity”. If the authors feel that carrying capacity + evasion is significantly different than population limits imposed by others it should be discussed, but in my mind the papers the authors compare to are all examples of non-linearly size-dependent branching process and it’s not a distinguishing factor of this model. That does not present a problem, but it seems that authors felt their work differs significantly in this regard (79-81, 630-637).

I would also argue that an increase in a mutational rate is not terribly useful to model in general, since it mainly increases the number of small clones in a later portion of simulation, which are usually ignored in summary statistics anyway. As it’s not listed in the phenotype of any fit in Fig.4., I guess the optimization process yields the same conclusion. Similarly, neutral mutations are modelled in other frameworks, but as they do not affect the behaviour, they are not part of the fit. This leaves only novelty in the Resource Control phenotype, which affects the context dependent competition between clones.The structure of the article makes it difficult to judge the benefit of the phenotype model, exacerbated by the fact that the mathematical formulation of the individual hallmarks is actually not in the main text. This I find to be an issue, since the result (Fig. 3, 4) fit the individual hallmarks and their numerical values, but from the main text e.g. the difference between Resource control (0.7, 1) and (0.1, 1) is not clear.

I find the resource control the most interesting part of the. Models like 38 or “Normal tissue architecture determines the evolutionary course of cancer, West, 2021” model a similar principle in the architecture of the tissue, while 39 uses abstract “local confinement”, however these are always symmetric between cell populations, whereas in this work a true heterogeneity arises. In general the effects of this per-clone specificity should be explained in more details.

In summary of the model, I found it difficult to gain understanding of the text, which can be improved by restructuring. As far as I can tell, the model is a more complex version of 38, 39 (more free parameters), therefore it is to be expected that the same dataset can be fitted. It should be shown that the inclusion of the Resource Control can add a mechanistic understanding of the system. An ablation study where the hallmarks are removed or limited (e.g. shared RC values for all clones) could demonstrate it.

On the results side I found the 433-436 surprising, to the level of being suspect. In Fig. 3 top left a neutral evolution is shown in a way I would not expect given the model. Principally speaking, if I have two clones, 1, 11, with p(11) = 1, and at a time t, population X_1(t) = 1000, X_11(t) = 1 and both have the same growth rate (only passenger mutations), then for t’ such that X_11(t’) = 1000, X_1(t’) should be ~1000000, i.e. it should not be possible to retain linearly proportional population in a system with exponential growth as long as the growth rate is the shared. Unless the individual populations are log transformed before plotting, I don’t understand how this is happening and it should be investigated/justified by the authors.

On the side of writing in general, I had the feeling the authors are mostly coming from mathematics and running the paper by a computational biologist in phylogenetics would be warranted. This is mainly evident in 128-140. As far as I can tell, LFORT is a multifurcating phylogenetic tree and does not need to be defined (potentially only in the supplement for the proof; since you in practice only consider finite depth trees, the potentially infinite depth of LFORT is in my opinion not a main matter of the paper). Similarly in 140-153 it seems that the text 4 times repeats that X_{node}(t) is a population of that node at time $t$, while stating that X_v is a symbol for both the ancestor population and the descendant population, based on $u$ which is not part of the X_v symbol… Please once formalize $X_u(t)$ (which is rather unclear in 146 currently) and move the definitions of phylogenetic relationships between nodes elsewhere, or omit altogether.

On the data side, as far as I can tell only the tool is available, but neither the simulated data, nor their analysis is made available. Either the results or a recipe for recreating the results using the tool should be made public.

Minor comments:

- Since the clonal nesting and clonal diversity are not developed here, they should go to methods, not results.

Cancer simulations tend to be computationally expensive, in particular when an interpreted language is used. A short summary of simulation and optimization compute costs should be included.

- The work presents a nice simulation web interface, however it is not publicly hosted and scientific clusters usually don’t allow running custom web servers with outside access. A hosted version would be certainly an advantage of this simulator over others.

- I think for each node u, the ph(u) is constant? Therefore a_{ph(u)} does not have to explicitly include (ph) and you can keep a(u) and b(u) without the replacement for a, b at 328? Also I think you’re referring to the application of a_{ph(u)} given X(t) in this line?

- 338 the M is I think related to m in 343 but it’s not very clear, also I’m not sure if p_n at 336 is needed and you already use p at 137.

- 228-238 - it seems that most of this text comes from explicitly treating passenger mutation differently. Since you already have variable parameter effects, could you not just say that a neutral mutation is a parameterless one and a parent differs from a child exactly by one mutation?

- 286 \mu seems to be a mutational rate from context, but not specified.

- 295 - what is \Omega? Also I think you are switching between a list of univariate functions and a multivariate function.

- 910-914 unclosed brackets

- In the default parameters of the simulation the mutation probability is set to 2.66×10-9, that seems rather low?

- Despite that, a simulation with 10 cores and otherwise default parameters has not terminated in an hour and there’s no progress reporting or logging, so I was not able to tell if there was any issue or if I was supposed to wait longer. (Tested on a node with 32 4Ghz cores and 128GB RAM).

Reviewer #3: This manuscript presents a stochastic tumor evolution framework that represents clonal lineages on a rooted ordered tree and maps genotypes to phenotypes via “functional events” (e.g., proliferative deregulation, mutation-rate increase, limit evasion, resource control), with resource-mediated competition and carrying-capacity constraints. The authors also provide a simulation algorithm and a GUI intended to facilitate exploration and reproducibility . They demonstrate that different evolutionary “paradigms” can arise under different parameter settings and validate against empirical tumor datasets using clonal diversity and clonal nesting metrics. Overall, the paper is well-written, the software and the problem are very interest, just unfortunate that I was unable to run it.

Major comments

1) The code seems interesting. However, I was unable to run it successfully on my machine.

My system

2.3 GHz Quad-Core Intel Core i7

Intel Iris Plus Graphics 1536 MB

16 GB 3733 MHz LPDDR4X

macOS 26.3 (25D125)

Docker

Docker version 29.2.1, build a5c7197

Docker Compose version v5.0.2

I followed the instructions from GitHub:

Clone the git with

git clone git@github.com:qBioTurin/CancerSimulationInterface.git

cd CancerSimulationInterface

Then run (MacOS/Linux):

docker compose up --build

After running docker compose up --build, my terminal showed:

Attaching to interface-1, simulator-1

simulator-1 | * Serving Flask app 'main'

simulator-1 | * Debug mode: off

simulator-1 | WARNING: This is a development server. Do not use it in a production deployment. Use a production WSGI server instead.

simulator-1 | * Running on all addresses (0.0.0.0)

simulator-1 | * Running on http://127.0.0.1:5000

simulator-1 | * Running on http://172.18.0.3:5000

simulator-1 | Press CTRL+C to quit

interface-1 |

interface-1 | > cancersimulator@0.1.0 start

interface-1 | > next start

interface-1 |

interface-1 | ▲ Next.js 15.3.3

interface-1 | - Local: http://localhost:3000

interface-1 | - Network: http://172.18.0.2:3000

interface-1 |

interface-1 | ✓ Starting...

interface-1 | ✓ Ready in 1021ms

I then opened http://localhost:3000 in my browser. The user interface loaded, but with the default parameters, when I clicked “Run Simulation,” the button showed a spinning circle and the app appeared to get stuck. I waited for almost two hours and nothing happened.

In the terminal, I repeatedly saw the following message:

simulator-1 | 172.18.0.2 - - [03/Mar/2026 22:27:38] "GET /check_percentage?checkpoints=50 HTTP/1.1" 500 -

I also tried reducing the simulated time span to 4 (in case the issue was the simulation length), but the same behavior occurred. Also, the progress bar in the interface did not move at all during this time. How long is a typical run expected to take with the default settings? Have you tested it on different machines, and could you provide approximate run-time metrics? (I did not change any parameters, including the advanced settings such as the number of threads.)

For reproducibility, I strongly encourage:

• a tagged release corresponding to the submitted manuscript;

• a minimal “reproduce figures” script in addition to the GUI;

• documentation of compute requirements and run times for the main experiments.

2) Clarify what is meant by “single-cell resolution”

The manuscript states that the “simulator tracks tumor evolution at single-cell resolution, recording for each cell its genotype and phenotype”. However, the described simulation step updates population sizes via multinomial sampling for each clone and then spawns new clones via Poisson sampling. That is a clone/population-level simulation (which is fine), but it is not obvious that individual-cell are explicitly tracked throughout time.

3) Biological interpretability of “functional events” and mapping to hallmark-like mechanisms

The phenotype mapping is grounded in hallmark-style functional categories and collapses them into five mechanisms plus “null effect”. This is conceptually appealing, but for readers it will be important to understand:

• how parameter ranges for each mechanism were chosen (especially “resource control” susceptibility/offensiveness and “limit evasion” as added capacity);

• whether the “relative frequencies” of events (r) are intended to reflect biology, sequencing panels, or are simply scenario knobs.

4) Spatial structure and interaction assumptions should be foregrounded in the main text

A key simplifying assumption is that “heterogeneity is always present,” meaning every cell competes/interacts with all others (global mixing). This is a major modeling choice with direct implications for clonal interference, coexistence, and the interpretation of “spatial” patterns inferred from simulated multi-region sequencing.

This limitation should be stated explicitly in the main paper (not only the supplement), and the authors should discuss which results are expected to be robust vs. which might change under local-neighborhood interactions. The discussion already contrasts spatial geometry vs resource control; it would help to connect that argument more directly to this assumption.

Minor comments / edits

“proliferative” appears corrupted in figure 1 text; please check figure label

The stopping criterion uses “detectable size” assumptions (e.g., 1 cm³ ~ 10^9 cells) and then adjusts because the model tracks only alive cells. Please state the implied alive fraction (even approximately) or cite justification.

**********

**Have the authors made all data and (if applicable) computational code underlying the findings in their manuscript fully available?**

The PLOS Data policy requires authors to make all data and code underlying the findings described in their manuscript fully available without restriction, with rare exception (please refer to the Data Availability Statement in the manuscript PDF file). The data and code should be provided as part of the manuscript or its supporting information, or deposited to a public repository. For example, in addition to summary statistics, the data points behind means, medians and variance measures should be available. If there are restrictions on publicly sharing data or code —e.g. participant privacy or use of data from a third party—those must be specified.

Reviewer #1: None

Reviewer #2: **No:**  The source data are declared; The software is Available; The simulation results and their analysis, or configuration to recreate these are missing.

Reviewer #3: None

**********

PLOS authors have the option to publish the peer review history of their article (what does this mean?). If published, this will include your full peer review and any attached files.

If you choose “no”, your identity will remain anonymous but your review may still be made public.

**Do you want your identity to be public for this peer review?**  For information about this choice, including consent withdrawal, please see our Privacy Policy.

Reviewer #1: No

Reviewer #2: No

Reviewer #3: No

[NOTE: If reviewer comments were submitted as an attachment file, they will be attached to this email and accessible via the submission site. Please log into your account, locate the manuscript record, and check for the action link "View Attachments". If this link does not appear, there are no attachment files.]

**Figure resubmission:**

While revising your submission, we strongly recommend that you use PLOS’s NAAS tool (https://ngplosjournals.pagemajik.ai/artanalysis) to test your figure files. NAAS can convert your figure files to the TIFF file type and meet basic requirements (such as print size, resolution), or provide you with a report on issues that do not meet our requirements and that NAAS cannot fix.

After uploading your figures to PLOS’s NAAS tool - https://ngplosjournals.pagemajik.ai/artanalysis, NAAS will process the files provided and display the results in the "Uploaded Files" section of the page as the processing is complete. If the uploaded figures meet our requirements (or NAAS is able to fix the files to meet our requirements), the figure will be marked as "fixed" above. If NAAS is unable to fix the files, a red "failed" label will appear above. When NAAS has confirmed that the figure files meet our requirements, please download the file via the download option, and include these NAAS processed figure files when submitting your revised manuscript.

**Reproducibility:**

To enhance the reproducibility of your results, we recommend that authors of applicable studies deposit laboratory protocols in protocols.io, where a protocol can be assigned its own identifier (DOI) such that it can be cited independently in the future. Additionally, PLOS ONE offers an option to publish peer-reviewed clinical study protocols. Read more information on sharing protocols at https://plos.org/protocols?utm_medium=editorial-email&utm_source=authorletters&utm_campaign=protocols

# Author comment: Author response to Decision Letter 1

**DOI:** 10.1371/journal.pcbi.1013991.r002

18 May 2026

Attachment Submitted filename: Response to reviewers.pdf

# Peer review: Decision Letter 1

**DOI:** 10.1371/journal.pcbi.1013991.r003

**Authors:** Pedro Mendes, Guillermo Lorenzo

3 Jun 2026

Dear Sirovich,

We are pleased to inform you that your manuscript 'A new cancer progression model: from synthetic tumors to real data and back' has been provisionally accepted for publication in PLOS Computational Biology.

Before your manuscript can be formally accepted you will need to complete some formatting changes, which you will receive in a follow up email. A member of our team will be in touch with a set of requests.

Please note that your manuscript will not be scheduled for publication until you have made the required changes, so a swift response is appreciated.

IMPORTANT: The editorial review process is now complete. PLOS will only permit corrections to spelling, formatting or significant scientific errors from this point onwards. Requests for major changes, or any which affect the scientific understanding of your work, will cause delays to the publication date of your manuscript.

Should you, your institution's press office or the journal office choose to press release your paper, you will automatically be opted out of early publication. We ask that you notify us now if you or your institution is planning to press release the article. All press must be co-ordinated with PLOS.

Thank you again for supporting Open Access publishing; we are looking forward to publishing your work in PLOS Computational Biology.

Best regards,

Pedro Mendes

Section Editor

PLOS Computational Biology

***********************************************************

Reviewer's Responses to Questions

**Comments to the Authors:**

**Please note here if the review is uploaded as an attachment.**

Reviewer #1: Thank you to the authors for addressing my previous comments. In particular, the updated Methods and Results sections are much more clear now, and distinguish the work from previous work in the field. This is a quite nice modeling framework, which I think should be published as a nice, unique addition to the field.

**********

**Have the authors made all data and (if applicable) computational code underlying the findings in their manuscript fully available?**

The PLOS Data policy requires authors to make all data and code underlying the findings described in their manuscript fully available without restriction, with rare exception (please refer to the Data Availability Statement in the manuscript PDF file). The data and code should be provided as part of the manuscript or its supporting information, or deposited to a public repository. For example, in addition to summary statistics, the data points behind means, medians and variance measures should be available. If there are restrictions on publicly sharing data or code —e.g. participant privacy or use of data from a third party—those must be specified.

Reviewer #1: Yes

**********

PLOS authors have the option to publish the peer review history of their article (what does this mean?). If published, this will include your full peer review and any attached files.

If you choose “no”, your identity will remain anonymous but your review may still be made public.

**Do you want your identity to be public for this peer review?**  For information about this choice, including consent withdrawal, please see our Privacy Policy.

Reviewer #1: No

# Editor report: Acceptance letter

**DOI:** 10.1371/journal.pcbi.1013991.r004

**Authors:** Pedro Mendes, Guillermo Lorenzo

PCOMPBIOL-D-26-00268R1

A new cancer progression model: from synthetic tumors to real data and back

Dear Dr Sirovich,

I am pleased to inform you that your manuscript has been formally accepted for publication in PLOS Computational Biology. Your manuscript is now with our production department and you will be notified of the publication date in due course.

The corresponding author will soon be receiving a typeset proof for review, to ensure errors have not been introduced during production. Please review the PDF proof of your manuscript carefully, as this is the last chance to correct any errors. Please note that major changes, or those which affect the scientific understanding of the work, will likely cause delays to the publication date of your manuscript.

Soon after your final files are uploaded, unless you have opted out, the early version of your manuscript will be published online. The date of the early version will be your article's publication date. The final article will be published to the same URL, and all versions of the paper will be accessible to readers.

For Research, Software, and Methods articles, you will receive an invoice from PLOS for your publication fee after your manuscript has reached the completed accept phase. If you receive an email requesting payment before acceptance or for any other service, this may be a phishing scheme. Learn how to identify phishing emails and protect your accounts at https://explore.plos.org/phishing.

Thank you again for supporting PLOS Computational Biology and open-access publishing. We are looking forward to publishing your work!

With kind regards,

Lilla Horvath

PLOS Computational Biology | Carlyle House, Carlyle Road, Cambridge CB4 3DN | United Kingdom ploscompbiol@plos.org | Phone +44 (0) 1223-442824 | ploscompbiol.org | @PLOSCompBiol
